# Origin and Evolution of Very Large Extracellular Proteins in Fructophilic Lactic Acid Bacteria

**DOI:** 10.1093/gbe/evag011

**Published:** 2026-01-22

**Authors:** Julia E Pedersen, Marina Mota-Merlo, Andrea Garcia-Montaner, Maria Selmer, Siv G E Andersson

**Affiliations:** Molecular Evolution, Department of Cell and Molecular Biology, Biomedical Centre, Science for Life Laboratory, Uppsala University, Uppsala 751 24, Sweden; Molecular Evolution, Department of Cell and Molecular Biology, Biomedical Centre, Science for Life Laboratory, Uppsala University, Uppsala 751 24, Sweden; Molecular Evolution, Department of Cell and Molecular Biology, Biomedical Centre, Science for Life Laboratory, Uppsala University, Uppsala 751 24, Sweden; Structural Biology, Department of Cell and Molecular Biology, Biomedical Centre, Uppsala University, Uppsala 751 24, Sweden; Molecular Evolution, Department of Cell and Molecular Biology, Biomedical Centre, Science for Life Laboratory, Uppsala University, Uppsala 751 24, Sweden

**Keywords:** fructophilic lactic acid bacteria, large extracellular proteins, substitution frequencies, recombination

## Abstract

Large surface proteins in bacteria serve important functions in aggregation, biofilm formation, and cell interaction processes. In *Apilactobacillus kunkeei*, a defensive symbiont of the honeybee *Apis mellifera*, as much as 6% of the 1.5 Mb genome consists of 5 consecutive genes for extracellular surface proteins of 3,000 to 8,000 amino acids, named Giant1-5. Here, we predict the structures of these proteins and provide a study of their origin and evolution. The structure predictions suggest that the Giant1-4 proteins contain a β-solenoid domain at their N-terminal ends with similarity to the β-solenoid domain in serine-rich repeat proteins, which mediates binding to glycoproteins, polysaccharides, and epithelial cells. Phylogenetic analyses based on the β-solenoid domains of the Giant1-3 proteins indicate sequence exchange between 2 genera of otherwise distantly related obligate fructophilic lactic acid bacteria, while the diversification of the positional homologs of the *giant*1-3 genes in the *A. kunkeei* population is mostly due to short, intra-genic recombination events. Genes for the Giant4-5 proteins were only identified in *A. kunkeei* and 2 closely related bacterial species, suggesting that they were added to the giant gene cluster more recently. The phylogenetic analyses indicate co-evolution of the *giant*4-5 genes in *A. kunkeei*, and the near sequence identity of one of the 2 *giant*4-5 subtypes correlates with predicted recombination events that span across both genes. Our findings provide new insights into the evolution of very large surface proteins in the bacterial ecosystem adapted to the carbohydrate-rich growth niches provided by bees, their food sources, and food products.

Significance Although the functions and molecular targets of extracellular surface proteins have been studied in many pathogens and model bacterial species, the molecular processes whereby they evolve have not been studied in depth due to rapid rates of sequence evolution and a lack of a sufficiently large number of genomes from the same species that encode such proteins. By analyzing a cluster of genes for very large surface proteins in a defensive symbiont of honeybees, we show that homologs to the first 3 genes in the cluster are broadly present in obligate fructophilic lactic acid bacteria, while the last 2 genes show a more restricted phyletic distribution pattern. This study provides insights into the origin and evolution of very large surface proteins in a bacterium that is of scientific as well as of commercial interest.

## Introduction

Honeybees are essential as pollinators. Therefore, understanding the nature of honeybee behavior and health is important. The honeybee gut microbiome influences the developmental pathways and is thought to protect the bees against pathogens (Engel et al. 2012; Kwong and Moran 2016; Lang et al. 2023; Motta and Moran 2024). The dominant bacterium in the honey stomach of the Western honeybee (*Apis mellifera*) is the fructophilic lactic acid bacterium *Apilactobacillus kunkeei* (Vásquez et al. 2012; Olofsson et al. 2014). This bacterium has also been identified in flowers, honey, bee bread, pollen, larvae, queens, and the hive apiary (Anderson et al. 2013; Anderson and Ricigliano 2017; Parish et al. 2022). Several isolates of *A. kunkeei* contain plasmids for the synthesis of antimicrobial peptides that specifically target bacterial pathogens of the larvae (Zendo et al. 2020; Dyrhage et al. 2022), which suggests that this bacterium plays a major role in honeybee health.

The *A. kunkeei* genome is small (1.5 Mb), despite of which it contains a 90 kb segment of 5 to 6 very long genes, referred to as the giant genes, which in total cover 6% of the genome (Djukic et al. 2015; Tamarit et al. 2015; Dyrhage et al. 2022). The encoded proteins are 3,000 to 8,000 amino acids long and contain an N-terminal signal peptide, a long, internal repeated region, and a conserved C-terminal segment (Tamarit et al. 2015). The giant proteins were identified in protein fractions obtained from the cell-free supernatant of *A. kunkeei* strains A0901 and A1401 after cultivation in fructose-supplemented media in the laboratory, suggesting an extracellular localization (Seeger et al. 2023). The findings that such a large fraction of a small bacterial genome contains multiple, consecutive genes for proteins of more than 3,000 amino acids that are present in all *A. kunkeei* genomes sequenced to date suggest that they serve a very important function.

Extracellular bacterial proteins may either be attached to the cell surface or secreted into the environment. These proteins play important roles in bacterial defense systems, host cell interactions, virulence, cell motility, and metabolism of carbohydrates and amino acids (Zhou et al. 2010; Roy et al. 2017). Extracellular proteins tend to be encoded by the accessory genome and show high rates of gene gain and loss (Rankin et al. 2011). Cell envelope proteins involved in cell-to-cell interactions evolve rapidly in sequence at nonsynonymous sites due to weak purifying selection, positive selection, or diversifying selection (Nogueira et al. 2009, 2012). Moreover, since extracellular proteins cannot be recycled, they tend to accumulate mutations that decrease the cost of synthesizing amino acids, resulting in high abundances of amino acids such as aspartate, asparagine, serine, and threonine (Reva and Tümmler 2008; Nogueira et al. 2009, 2012; Rankin et al. 2011).

Serine-rich repeat proteins (SRRPs) represent a distinct family of large surface proteins in gram-positive bacteria (Latousakis et al. 2020; Palomino et al. 2023; Cinar et al. 2024). They show a conserved domain organization with a unique domain at the N-terminus involved in the binding to bacterial or host cell surfaces, lengthy serine-rich repeat regions, and a cell wall anchor domain at the C-terminal end. The serine-rich repeat regions in these proteins are glycosylated, which adds stability to the stalk-like structure of the proteins to support adhesion, aggregation, and biofilm formation (Latousakis et al. 2019). Genes for SRRPs have for example been identified in strains of *Limosilactobacillus reuteri* strains, which colonize the proximal region of the gastrointestinal tract in rodents and pigs, but they are not present in environmental isolates of the same species (Frese et al. 2013). The SRRPs in *L. reuteri* contain 2 serine-rich repeat regions that flank a unique β-solenoid domain, which mediates the interactions with host epithelial cells and oligosaccharides in a pH-dependent manner (Sequeira et al. 2018). Likewise, the SRRPs of *Lactiplantibacillus plantarum* isolated from the gut of *Drosophila melanogaster* mediate binding to the host cell foregut (Gutiérrez-García et al. 2024). Interestingly, it was found that the genes for the SRRPs and their secretion system in *L. plantarum* were located on a plasmid, referred to as a colonization island, and thus belong to the accessory genome of the species (Gutiérrez-García et al. 2024).

Most studies on surface-exposed extracellular proteins performed so far have focused on their amino acid composition patterns, their functions and binding targets, and their association with mobile genetic elements. To understand the interplay between the underlying mechanisms and the selective forces that drive amino acid usage and gene content patterns, studies of multiple genomes from very closely related strains within a species are needed. However, there is still a scarcity of genomic data from multiple strains of a single species that contain genes for extracellular proteins of several thousand amino acids.

Since the first discovery of the giant gene cluster in *A. kunkeei* (Tamarit et al. 2015), more than 100 closed *A. kunkeei* genomes have become available (Dyrhage et al. 2022), enabling detailed investigations into their origins and the mechanisms whereby the giant genes diverge within the population. In addition, the development of AlphaFold (Jumper et al. 2021; Abramson et al. 2024) offers a new opportunity to infer the structural features of proteins with unknown functions and few homologs in other species. However, protein structure predictions can still not be done routinely for very large proteins. This study presents the first predicted structure models of the full-length giant proteins in *A. kunkeei* and investigates the processes that have driven their evolution. We suggest that the acquisition, multiplication, and diversification of the giant genes represent key events in the evolutionary history of the *Apilactobacillus* species, which laid the foundation for their colonization of the gut and carbohydrate-rich food sources of social insects.

## Results

### Predictions of the Structures of the Giant Proteins in *A. kunkeei* Strain A0901

**Fig. 1.**
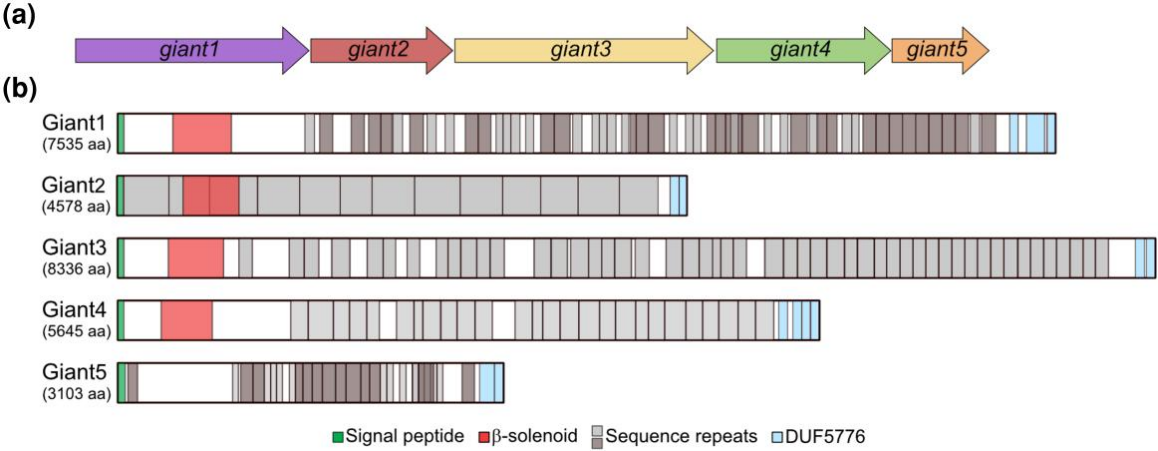
Giant gene order and domain architecture. Schematic representation of a) the *giant*1-5 gene order structure and b) the domain architecture and repeat organizations of each protein in *A. kunkeei* strain A0901. The N-terminal β-solenoid domain was predicted with the aid of AlphaFold3, the internal repeats with RADAR, and the C-terminal DUF5776 domain was predicted by searches with InterProScan.

We started out by comparing the amino acid contents and the domain architectures of the giant proteins in *A. kunkeei* strain A0901, naming them Giant1-5 based on the order of their genes (Fig. 1). All 5 proteins share a similar domain organization consisting of a signal peptide (Sec/SP1) at the N-terminal end, a long, internal segment of repeated sequences, and 2 to 4 predicted Pfam domains of unknown functions (DUF5776) at the C-terminal end (Table S1). The internal segments were composed of highly imperfect repeats in all proteins, including 23 repeats of 62 amino acids and 28 repeats of 103 amino acids in Giant1, 13 repeats of 366 amino acids in Giant2, 51 repeats of 103 amino acids in Giant3, 27 repeats of 136 amino acids in Giant 4 and finally 13 repeats of 126 amino acids and 13 repeats 50 amino acids in Giant5. All proteins have highly atypical amino acid contents with a high content of asparagine (9.7% to 11.6%, compared to 6% across all A0901 proteins), 4 proteins are also alanine-rich (14.4% to 18.6%, compared to 6.4%), and the Giant5 protein is serine-rich (17.5%, compared to 6.4%) (Table S2).

**Fig. 2.**
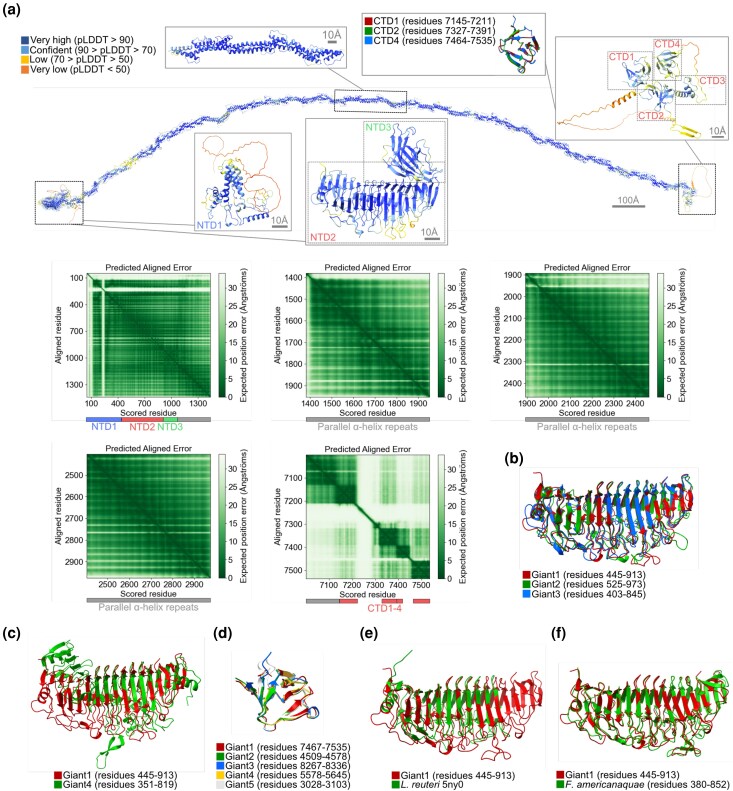
AlphaFold3 predictions and Foldseek homology searches. a) The AlphaFold3 model of Giant1 (scale bar 100 Å, colored according to pLDDT), with close-ups of the N-terminal domains (NTD1-3, scale bar 10 Å), the parallel α-helical repeats (scale bar 10 Å), and the C-terminal domains (CTD1-4, scale bar 10 Å), together with a structural alignment of CTD1-4 to display their similarity. PAE plots for N-terminal residues 50 to 2880 and C-terminal residues 6601 to 7535 are displayed, with the expected position error displayed in Ångströms. Structural alignment of the β-solenoid domain in b) the Giant1-3 proteins, c) the Giant1 and Giant4 proteins, d) the absolute C-terminal domain in the Giant1-5 proteins, e) the β-solenoid domains in the Giant1 protein and the SRRP protein in *L. reuteri*, and f) the Giant1 protein and its homolog in *F. americanaquae*.

AlphaFold3 was used to predict the 3D structures of the 5 giant proteins in *A. kunkeei* strain A0901 (Fig. 2; Figs. S1 to S5). The major parts of the Giant1-3 models consisted of parallel α-helical repeats, corresponding to the internal repeat segments (Fig. 1). The Giant1 protein (Fig. 2a) was further predicted to contain a disordered N-terminal end, a segment of α-helical structure (NTD1), a β-solenoid domain (NTD2), and a β-sheet domain (NTD3). At the C-terminal end, the prediction showed 3 β-sheet domains (CTD1-2, CTD4), overlapping with the predicted Pfam domains of unknown function (DUF5776), and a shorter domain (CTD3). The overall domain architectures of the Giant2 and Giant3 proteins (Figs. S2a and S3a) were similar to the Giant1 protein (Fig. 2a), including a similar β-solenoid domain (NTD2) (Fig. 2b). However, the prediction of the N-terminal end of the Giant2 protein showed additional α-helices and a higher confidence than that of Giant1 and Giant3 (Fig. S3c, Table S3b).

The predictions of the Giant1-3 proteins were mostly confident or of very high confidence, for example, most of the Giant2 protein domains showed average pLDDT >84 (Table S3b). Only a small part of the predictions had low confidence, often lacking secondary structure, indicating potential disorder. The predicted aligned error (PAE) plots for Giant1 (Fig. 2a) indicated that NTD1-3 pack into one stable structural unit, where only the low-confidence regions in NTD1 (pLDDT <50) showed high positional error, whereas CTD1-4 displayed a beads-on-a-string pattern with low confidence in the relative domain arrangement. The PAE plots of the helical repeats showed relatively low positional errors on the scale of +/− 150 amino acids or more, in line with that the parallel arrangement of helices leads to relatively stiff segments of structure with limited internal flexibility (up to 10% to 20% deviation per length). To investigate this further, the 5,680 residue Giant1 helical repeats segment was divided into 3, 5, 7, 9, and 11 sequence fragments for structure prediction in AlphaFold3 (Table S3a). With the exception of the longest (∼1,900 residue) fragments, all predictions resulted in elongated structures (Fig. S1a to e). A similar approach was applied to prediction of Giant2-3 structures, although only the shortest and longest sequence fragments were used as inputs (Table S3a), resulting in similarly elongated structures for shorter input fragments, and hinge regions of higher flexibility appearing for longer fragments (Figs. S2b and S3b). For Giant1, the first such hinge region was predicted around position 2,250 (Fig. S1e), whereas the PAE plots of the shorter input Giant1 predictions show no signs of hinges or higher-flexibility regions (Fig. S1a–d). A region of higher flexibility was consistently predicted around position 5,100 in all Giant1 models, except from the longest input where this coincides with a break between input segments. Thus, for clarity, the parallel α-helical repeats are visualized in extended conformations for the full-length Giant1-3 proteins (Fig. 2a; Figs. S2a and S3a). In this conformation, the Giant3 protein would reach an estimated total length of 0.2 µm (Fig. S3a). The PAE plots of the Giant2-3 proteins (Figs. S2a and S3a) displayed similar patterns as for the Giant1 protein in the N-terminal and α-helical repeat regions. For the C-terminal domains, the PAE plots support the packing of CTD1-2 into one structural unit together with the last section of α-helical repeats, forming a 300-residue unit with a flexible linker to the rest of the structure.

For the Giant4 protein, the prediction of the N-terminal end showed a disordered region, α-helices, and a β-solenoid domain, similar to the Giant1 protein (Fig. 2c; Fig. S4a to d). However, apart from a short α-helical structure, most of the protein was predicted to consist of a long array of β-sheet domains. Overall, the PAE plots show low confidence in the inter-domain packing and arrangement of the Giant4 domains, suggesting higher flexibility of the array of β-sheet domains compared to the elongated α-helical repeats in Giant1-3. A prediction of longer input sequence fragments of the internal repeats segment was also tested for Giant4, resulting in a lower confidence model (Fig. S4e). The prediction of the Giant5 protein structure (Fig. S5a, b) showed a disordered N-terminal segment followed by 9 β-sheet domains, appearing as beads on a string in the PAE plots, followed by a 2,000 amino acid long, single α-helix until the C-terminal domains. This structure was predicted with lower overall confidence. The PAE plots suggest that some positions along the helix are more flexible, and in models from different lengths of input sequences, the helix folds back on itself (Fig. S5c and d). Like the Giant1-3 proteins, the Giant4-5 proteins also contained short β-sheet domains at the C-terminal end (Fig. 2d). Analysis of the electrostatic surface potential (ESP) displayed a clear positive charge of the C-terminal end in all Giant1-5 proteins (Fig. S6a to e).

To infer the putative function of the 5 giant proteins in *A. kunkeei*, we performed structural homology searches to proteins for which the functions and structures have been experimentally determined or predicted. We used each domain in each protein as the query in searches with the aid of Foldseek, using the Foldseek web server (van Kempen et al. 2024), and set the *E*-value cutoff to <0.01. Hits to experimentally determined structures were found for all giant proteins. Interestingly, the results showed that the predicted β-solenoid domains of the Giants1-3 proteins aligned well with the β-solenoid domain of the SRRP from *L. reuteri* (Fig. 2e). The β-solenoid domain in the Giant4 protein also aligned with this domain, although it contained multiple short insertions in loop regions on one side of the β-solenoid, that were not present in any of the β-solenoid domains in the other giant proteins, nor in the SRRPs (Fig. S7, Table S4a). However, the amino acid sequence identities between the β-solenoid domains in the SRRP in *L. reuteri* and the giant proteins in *A. kunkeei* were only about 20%, and the amino acids demonstrated to be involved in ligand binding were not conserved (Fig. S7). Nor was the AST domain of 30 to 40 amino acids, which is used for targeting the SRRPs to the accessory Sec2 pathway, present in any giant protein. Additionally, the short β-sheet domain following the β-solenoid domain in the Giant1-3 proteins showed structural similarity to immunoglobulin-like domains (Figs. S3d and S8a and b). Finally, the C-terminal domain common to all 5 giant proteins shared structural similarity to the cell-wall binding SH3b domain (Fig. S8c–d) (Mitkowski et al. 2019; Cifuente et al. 2023), although the inter-domain arrangements in Giant2 and Giant5 were not found in any other proteins (Fig. S8e–g).

### Analyses of the Phyletic Distribution Profiles of the Giant Proteins

To identify homologs with β-solenoid domains in more closely related species for which the structures have not yet been determined or predicted, we searched the NR database with the aid of BlastP using the amino acid sequences of the β-solenoid domains in *A. kunkeei* strains A0901, A1001, and A1404 as the queries, under the assumption that this domain is more conserved than the rest of the protein. Strong hits were obtained in this search to proteins encoded by genes in most *Apilactobacillus* and *Fructobacillus* genomes (*E*-value < 1e^−30^) (Table S4b). Weaker hits with less coverage over the β-solenoid domain were also obtained to proteins in a few species of *Lactobacillus* and *Nicoliella* (1e^−20^ < *E*-value < 1e^−10^).

**Fig. 3.**
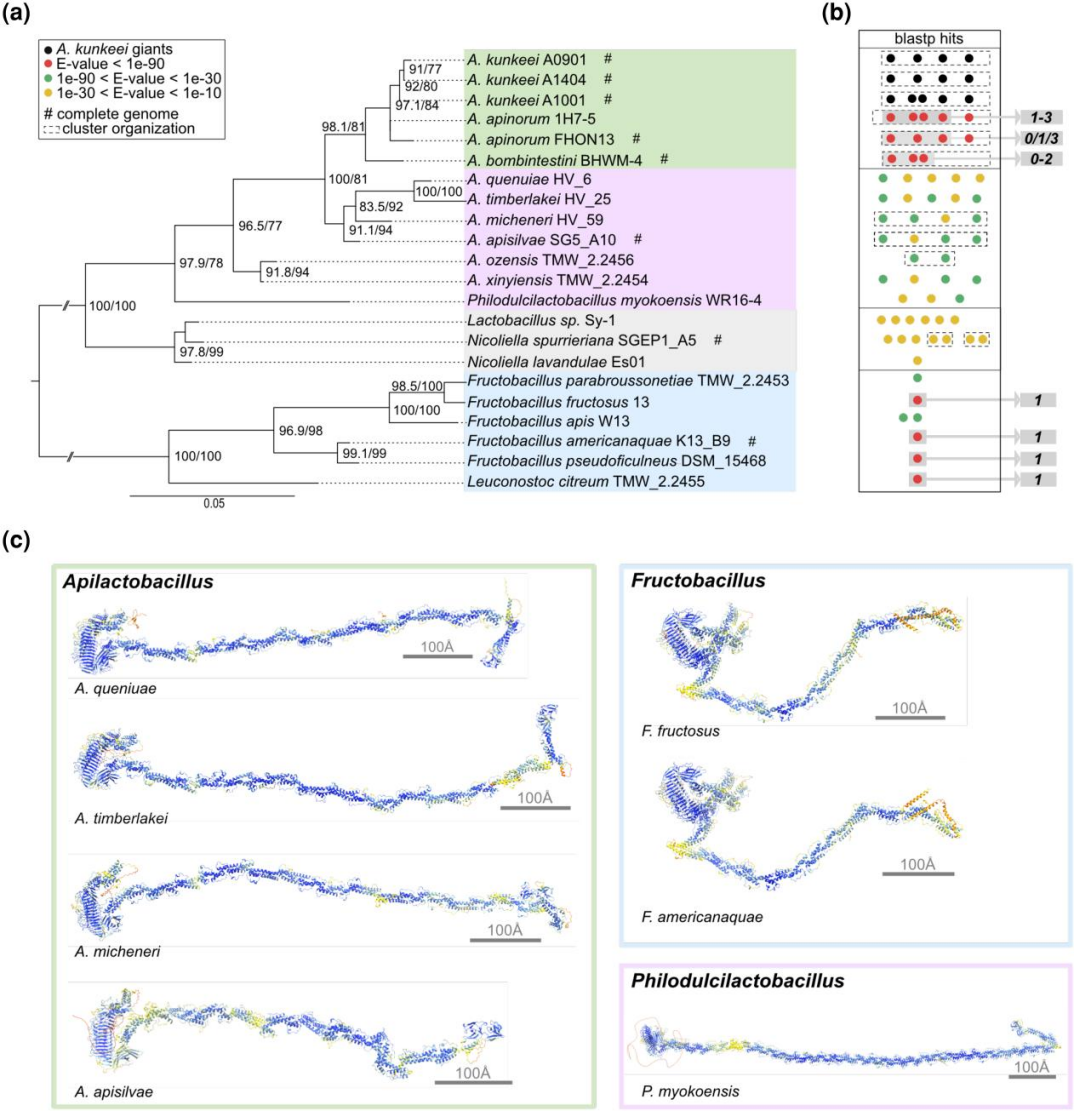
Giant protein homologs in fructophilic lactic acid bacteria. a) The 16S rRNA tree was inferred for species for which hits were obtained to the β-solenoid domains of the *A. kunkeei* Giant1-3 proteins in BlastP searches. The tree was midpoint rooted, and the numbers at the nodes show the statistical support as indicated by 1,000 ultrafast bootstraps and 1,000 SH-like pseudoreplicates. Only values above 75% are shown. b) The number of proteins in each species for which BlastP hits were obtained is displayed as circles, which have been color-coded according to the *E*-value interval of the hit. The Giant proteins in *A. kunkeei* with β-solenoid domains are displayed as black circles. The numbers 0 to 3 indicate the number of the giant gene in *A. kunkeei* for which a hit was obtained with an *E*-value < 1e^−90^. A dashed rectangle surrounding the circles indicates that the genes are organized into a gene cluster. c) AlphaFold3 models of the most complete and highly supported hits (scale bar 100 Å or 10 Å, color coded according to pLDDT).

To place the BLAST hits in a phylogenetic framework, we inferred a 16S rRNA tree for the species to which the best hits were obtained. The phylogeny confirmed that all *Apilactobacillus* species formed a clade with 96% to 97% bootstrap support (Fig. 3a; Fig. S9; Table S4c and d). The clade encompassing the *Fructobacillus* species showed a rather distant relationship to the *Apilactobacillus* species, with 16S rRNA sequence identity values in the range of 83% to 85% (Table S4c).

We then mapped the BLAST hits onto the 16S rRNA phylogeny (Fig. 3b). The BLAST hits included proteins with β-solenoid encoded by a cluster of 3 to 5 consecutive genes in *A. kunkeei*, *A. apinorum*, and *A. bombintestini* (Fig. 3b; Fig. S10). The gene clusters in these species were flanked by upstream genes for purine (*purB*) and pyrimidine (*pyrB*) biosynthesis and by downstream genes for glycosyl hydrolases (*tarM* and *gtfA*), followed by a conserved stretch of genes including *rlmD* and *rsmG* (Fig. S11a and b). Likewise, hits were obtained to the β-solenoid domains in proteins of 2,500 to 5,500 amino acids encoded by a cluster of 4 consecutive genes in the closed genome of *A. apisilvae* (*E*-value <1e^−30^). Interestingly, the gene clusters in *A. apisilvae* as well as in *A. quenuiae*, *A. timberlakei*, and *A. micheneri* were located upstream of genes that included *rlmD* and *rsmG* (Fig. S11a), adding further indirect support to the blast-based inferences of homology between the giant gene clusters in these species.

Remarkably, a stronger hit was obtained to the β-solenoid domain of a protein of 3,500 amino acids encoded by a single gene in the closed genome of *Fructobacillus americanaquae* (*E*-value <1e^−150^) than the hits obtained to proteins in *A. apsilvae* (*E*-value <1e^−30^) (Fig. 3b; Table S4d). Very strong hits were also obtained to the β-solenoid domains of proteins encoded by genes located on single contigs in *Fructobacillus fructosus*, *Fructobacillus pseudoficulneus*, and *Leuconostoc citreum* (*E*-value <1e^−140^) (Fig. S10). Finally, significant hits were obtained to genes in a few additional *Apilactobacillus* and *Fructobacillus* species. However, the number, order, and length of genes in the assembled contigs of these additional species are less reliable due to their incomplete status.

The structures of the homologs identified in the *Apilactobacillus* and *Fructobacillus* species were predicted with AlphaFold3, and the results confirmed the presence of a β-solenoid domain in the N-terminal end, followed by a long rod-like structure consisting of similar α-helical repeats (Fig. 3c). Interestingly, the structure of the predicted β-solenoid domain of *F. americanaquae* was similar to that of the Giant1 protein (Fig. 2f). The β-solenoid domains in *A. kunkeei* strain A1001 and *F. americanaquae*/*F. fructosus* showed 67% sequence identity, but the sequence similarity extended over the β-solenoid domain and another 150 amino acids (Fig. S11c). Furthermore, all proteins identified in the *Apilactobacillus* species contained an SH3b-like domain at the C-terminal end, whereas the homologs encoded by the *F. americanaquae* and *F. fructosus* genomes were shorter and lacked about 1,000 amino acids at the C-terminal end (Fig. S11c).

The Giant5 protein differed from the other giant proteins in that it contained no β-solenoid domain, and no structural similarity to proteins with an experimentally determined or predicted structure was found. However, searches for homologs with the aid of BlastP using the entire Giant5 protein in *A. kunkeei* as the query yielded very strong hits to proteins in *A. apinorum* and *A. bombintestini* (*E*-value <1e^−130^) (Table S4e), which were encoded by genes located downstream of the cluster of genes for the Giant1-4 proteins, as in *A. kunkeei* (Fig. 1; Fig. S10). Weaker hits were also obtained to proteins in other species (1e^−20^ < *E*-value < 1e^−10^), but the sequence similarity encompassed only the SH3b-like domain (Table S4e).

### Phylogenetic Analyses of the Giant Proteins in *Apilactobacillus* and *Fructobacillus*

**Fig. 4.**
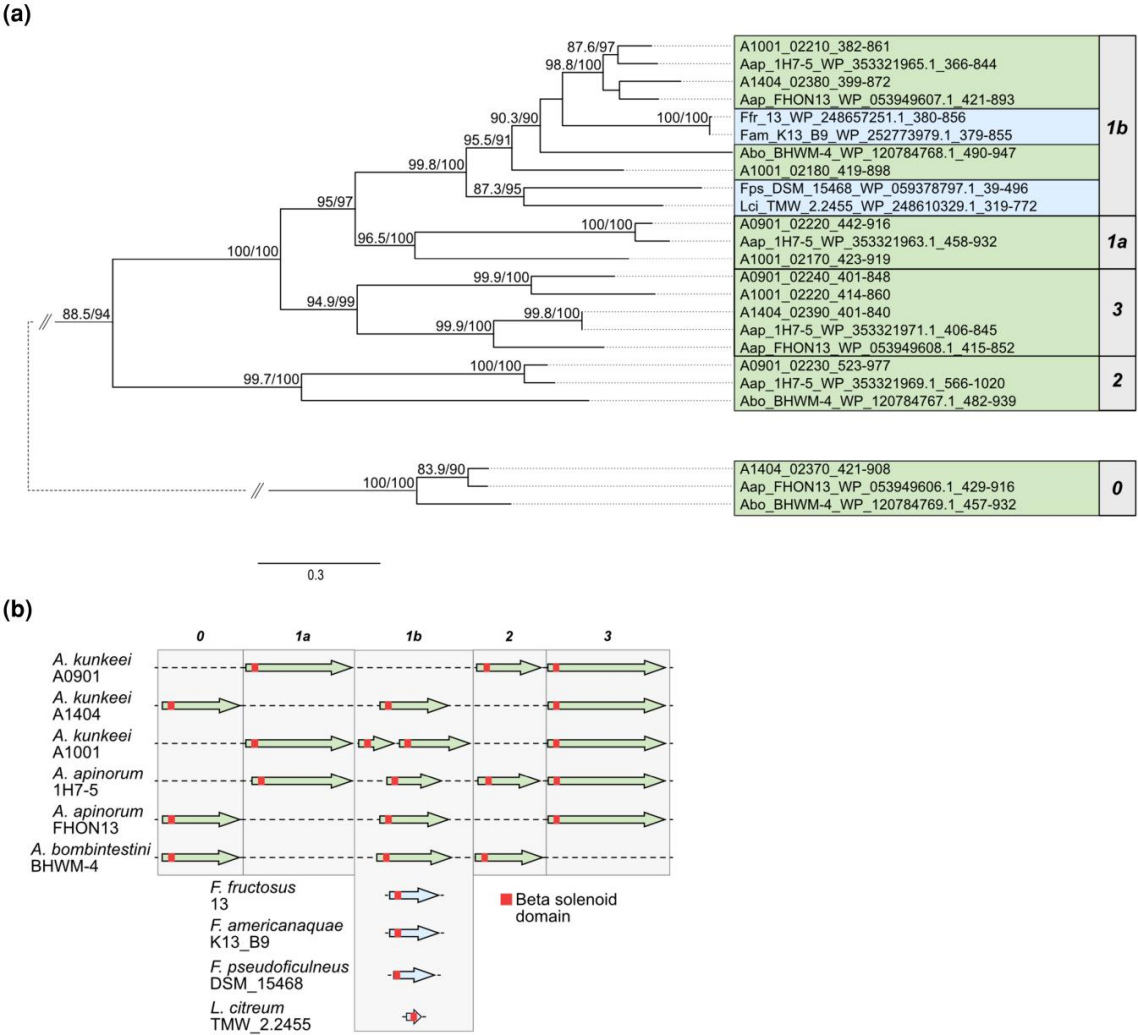
Phylogeny inferred from the β-solenoid domain sequences. a) The tree shows a phylogeny inferred from β-solenoid domains in proteins for which a hit was obtained to the β-solenoid domains in the *A. kunkeei* Giant1-3 proteins in BlastP searches. The tree has been extracted from Fig. S13 and includes a subset of taxa that yielded hits with *E*-value <1e^−90^. *A. kunkeei*, *A. apinorum* (Aap), and *A. bombintestini* (Abo) are marked in light green, and *Fructobacillus* and *Leuconostoc* species (Ffr, Fam, Fps, and Lci) are marked in light blue. The numbers 0 to 3 refer to the Giant0-3 proteins in *A. kunkeei*. The 2 sister clades of the β-solenoid domains in the Giant1 clade have been named 1a and 1b. The taxa descriptions include species abbreviations or *A. kunkeei* strain designations, protein accession numbers or *A. kunkeei* locus tags, and the position of the β-solenoid domain sequence in the protein used for the phylogeny. The numbers at the nodes of the tree show the statistical support as indicated by 1,000 ultrafast bootstraps and 1,000 SH-like pseudoreplicates. Only values above 75% are shown. b) The order and lengths of genes for the Giant0-3 proteins in *A. kunkeei*, *A. apinorum*, *A. bombintestini*, *F. fructosus*, *F. americanaquae*, *F. pseudoficulneus*, and *L. citreum* are shown in relation to each other. The location of the sequence encoding the β-solenoid domain is highlighted in red.

The high sequence similarities of the β-solenoid domains in the otherwise rather distantly related species of *Apilactobacillus* and *Fructobacillus* merited further investigations. To begin, we inferred a phylogeny based on the aligned amino acid sequences of the β-solenoid domains in all taxa that showed a hit with *E* < 1e^−10^ (Fig. S12; Table S4b). The tree confirmed the deep divergence of the β-solenoid domain in the Giant4 proteins in relation to the clades encompassing the Giant1-3 proteins. Next, we filtered out all sequences that clustered with or basal to the Giant4 clade as well as β-solenoid domains in proteins of < 1,000 amino acids and we also excluded taxa with more than 99.5% 16S rRNA sequence similarity to an already selected taxon. A new phylogenetic tree was inferred based on this smaller subset of sequences, which showed that the β-solenoid domains in the Giant1, Giant2 and Giant3 proteins in *A. kunkeei* strain A0901 formed 3 distinct clades, of which the Giant1 and Giant3 clade clustered with 100% bootstrap support to the exclusion of the Giant2 clade (Fig. 4a; Fig. S13, Table S4d).

The Giant1 clade was further subdivided into 2 smaller sister clades, which we refer to as Giant1a and Giant1b. The Giant1a protein was encoded by the first gene in the giant gene cluster in *A. kunkeei* A0901, A1001 and *A. apinorum* 1H7-5, whereas the Giant1b protein was encoded by the second gene in the cluster in *A. kunkeei* A1404, A1001 (which contained 2 copies), *A. apinorum* 1H7-5, FHON13 and *A. bombintestini* (Fig. 4b). In genomes that contained both variants, the gene for the Giant1a protein was located immediately upstream of the gene for the Giant1b protein. Interestingly, the single giant proteins identified in *F. americanaquae*, *F. fructosus*, *F. pseudoficulneus*, and *L. citreum* belonged to the Giant1b subclade, and clustered with *A. kunkeei* A1001, *A. apinorum*, and *A. bombitestini* with 99% to 100% bootstrap support (Fig. 4a; Fig. S13). It was also noted that the β-solenoid domains in the giant proteins of *F. americanaquae* and *F. fructosus* were 99.6% identical in sequence (Fig. 4a; Fig. S13), although the 2 species belong to different clades in the 16S rRNA tree (Fig. 3a).

The gene for the Giant1a protein was missing in *A. kunkeei* A1404, *A. apinorum* FHON13, and *A. bombintestini*, which instead contained a deeply diverging homolog of more than 5,000 amino acids, here called Giant0, which was located immediately upstream of the gene for the Giant1b protein (Fig. 4b). The species examined here contained either genes for Giant0-Giant1b or for Giant1a-Giant1b. Several other deeply diverging giant protein variants were also identified in most of the other *Apilactobacillus* species as well as in *Fructobacillus apis* W13 and *Fructobacillus parabroussonetiae* TMW_2_2453 (Figs. S10 and S13).

### Phylogenetic Analyses of the Giant Proteins in the *A. kunkeei* Population

To study the patterns whereby the giant proteins have diversified within a species, we selected one strain from each of the 34 *A. kunkeei* lineages previously shown to represent the genetic diversity of the 104 isolates for which closed genomes are available (Dyrhage et al. 2022). The large majority, 32 lineages in total, have been assigned to phylogroups A to C, while the remaining 2 lineages represent more distantly related strains that have been assigned to phylogroups E and F, according to the classification by Dyrhage et al. (2022).

We noted that 3 of the 34 reference strains contained giant genes with internal termination codons, one of which was found to be a previous sequencing error, and the sequence was edited accordingly (Fig. S14a–d; Tables S5 and S6). However, the *giant*5 gene in *A. kunkeei* strain H4B5-04J contained a confirmed termination codon (Fig. S15a–d) and this gene was therefore excluded from the analyses. Furthermore, strain H4B4-06M contained possible pseudogenization events in 2 giant genes, and this strain was excluded from the analyses, resulting in a total of 33 *A. kunkeei* genomes (Fig. S16).

The median amino acid sequence identity values of all pairwise comparisons of the full-length protein sequences in strains assigned to phylogroups A, B, and C ranged from 93% for the Giant1 and Giant3 proteins to 98% for the Giant4 and Giant5 proteins, whereas the identities between them were only about 20% (Tables 1 and 2, Table S7). Furthermore, the giant protein encoded by the first gene in strain H1B1-04J was an outlier with an amino acid sequence identity of only 20% to 22% to the Giant1 proteins in the other phylogroup A strains. A phylogeny inferred from the β-solenoid domains confirmed that the giant proteins in all of the examined *A. kunkeei* strains clustered within either of the Giant1, Giant2, Giant3, and Giant4 clades, with the exception of the protein encoded by the first gene in strain H1B1-04J, which clustered with the Giant0 protein in strain A1404 (Fig. S12).

**Table 1:** Mean and median protein sequence identities of the Giant1-5 proteins in *A. kunkeei* phylogroup A to C strains

Identity (%)	Giant1^a^	Giant2^b^	Giant3^c^	Giant4^d^	Giant5^e^
Mean	90.5	90.0	95.2	78.9	88.1
Median	92.9	92.9	95.7	98.1	97.6

Number of ^a^Giant1 and ^e^Giant5 strains = 30. Number of ^b^Giant2, ^c^Giant3 and ^d^Giant4 strains = 31.

**Table 2:** Range of protein sequence identity values (including minimum and maximum values) of the giant proteins in pairwise comparisons of *A. kunkeei* strains of phylogroups A to C

Identity range (%)	Giant1^a^	Giant2^a^	Giant3^a^	Giant4^a^	Giant5^a^
Giant1	**80.8 to 100**	17.3 to 22.3	30.2 to 31.4	18.6 to 20.8	9.9 to 12.2
Giant2	–	**63.4 to 100**	15.4 to 21.2	19.1 to 21.2	16.5 to 21.9
Giant3	…	–	**82.2 to 100**	14.5 to 19.6	9.1 to 10.8
Giant4	…	…	–	**45.8 to 100**	11.9 to 14.2
Giant5	…	…	…	–	**68.5 to 100**

^a^Comparisons of proteins encoded by genes that are positional homologs are shown in bold.

**Fig. 5.**
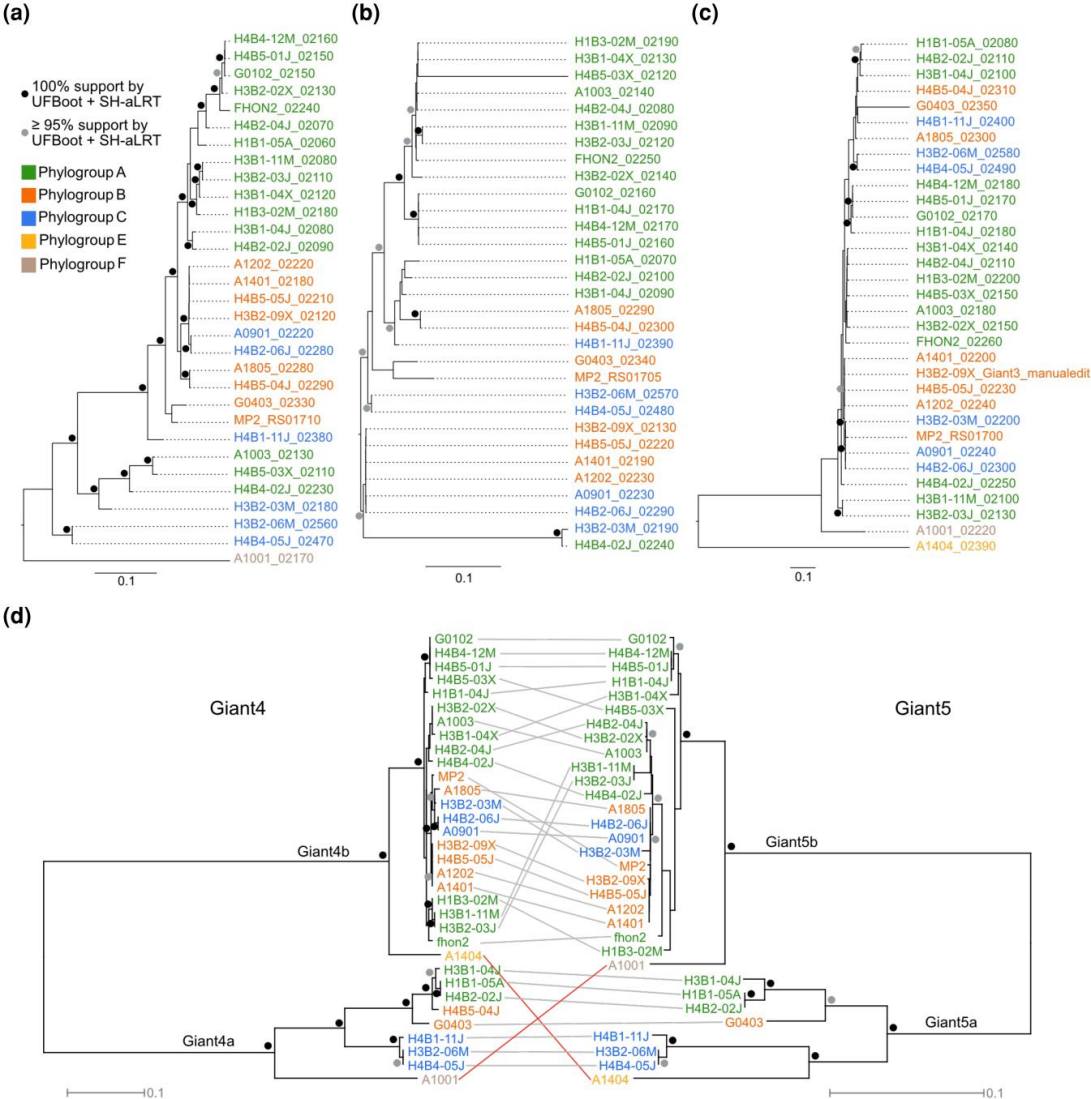
Phylogenies inferred from the Giant protein sequences in *A. kunkeei*. Phylogenies inferred from the a) Giant1, b) Giant2, and c) Giant3 protein sequences in *A. kunkeei*. d) A comparison of the Giant4 and the Giant5 tree topologies. The taxa descriptions include *A. kunkeei* strain designations (for Giant4-5) and locus tags (for Giant1-3). Circles at nodes refer to the statistical support inferred from 1,000 ultrafast bootstraps and 1,000 SH-like pseudoreplicates. Incongruencies in the divergence patterns between nodes with 100% support are displayed as red lines.

Based on this result, we inferred maximum likelihood trees for each protein family separately using the full-length protein sequences (Fig. S17). In all of the individual subtrees, several highly supported clades contained a mixture of strains from different phylogroups (Fig. 5; Fig. S17). Thus, none of the protein trees inferred from the Giant proteins were consistent with the phylogroup designations. However, unlike the Giant1-3 tree topologies which presented incongruent tree topologies (Fig. S18), the diversification patterns of the Giant4 and Giant5 protein trees were mostly congruent (Fig. 5d). The Giant4 and Giant5 protein trees consisted of 2 subclades separated by 100% bootstrap support, of which subclade *b* contained highly similar sequences from 2 thirds of the strains, while subclade *a* contained highly divergent sequences from the remaining one third of the strains. The only significant difference between the Giant4 and Giant5 protein trees concerned the placement of the phylogroup E strain A1404 and the phylogroup F strain A1001, which clustered differently with either of the 2 subclades.

### Predictions of Recombination Tracts in the Giant Gene Cluster in *A. kunkeei*

Next, we estimated the pairwise nucleotide substitution frequencies at nonsynonymous (d*N*) and synonymous (d*S*) sites (Table S8), while being aware that the observed sequence divergences may be an effect of both nucleotide substitutions and recombination events. The median d*N* values ranged from 0.01 to 0.04 substitutions per nonsynonymous site for the giant genes, while the median d*S* values of the giant genes ranged from 0.04 to 0.31 substitutions per site. The median d*S* values of the giant genes were consistently 4- to 8-fold higher than the median d*N* values, confirming that the giant genes evolve under purifying selection.

**Fig. 6.**
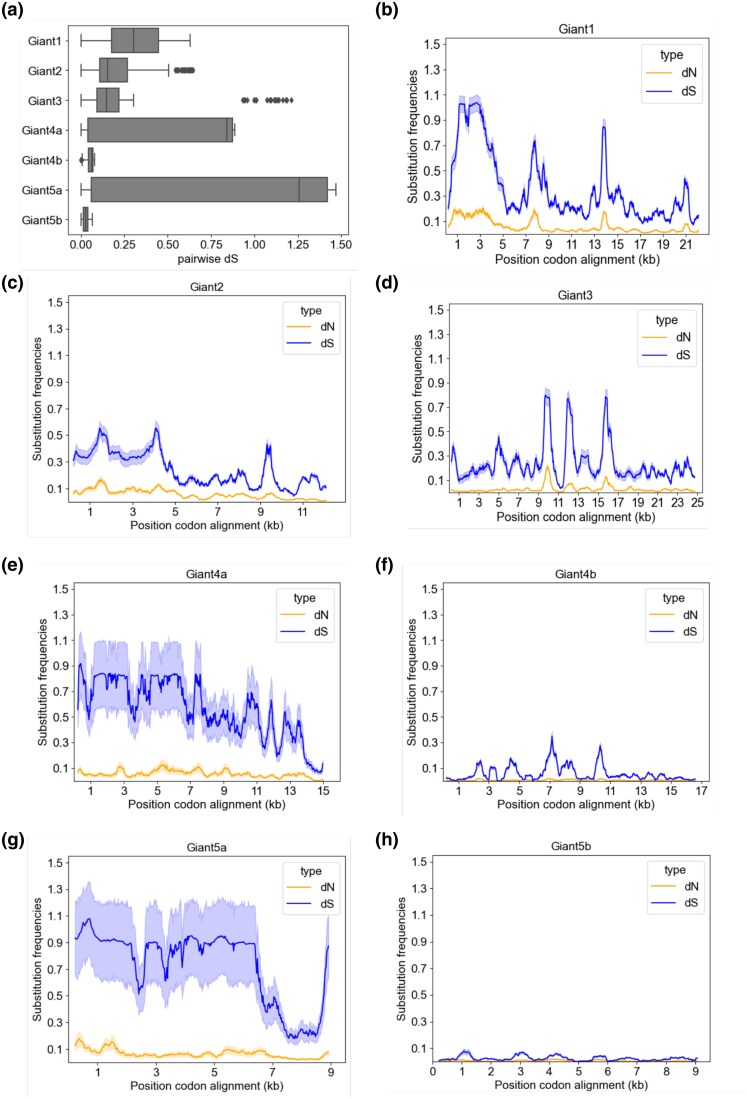
Substitution frequencies in the giant genes at synonymous and nonsynonymous sites. Estimates of synonymous (d*S*) and nonsynonymous (d*N*) substitutions in the giant genes in pairwise comparisons of *A. kunkeei* strains of phylogroups A to C. a) The mean d*S* value for the whole gene is displayed in a boxplot. b–h) The mean d*S* and d*N* values are displayed in a sliding window analysis using a 450 bp window with a step size 21 bp. All pairwise d*S* values ≥ 1.5 were considered to be saturated and set to 1.5.

However, there was a dramatic variability in sequence divergence levels that depended both on the genes and on the pair of strains being compared. For example, the d*S* values for the 2 subtypes of the *giant*4 and *giant*5 genes varied by a factor of more than 30, ranging from a median value of only 0.03 to 0.06 substitutions per synonymous site for the *giant*4b and *giant*5b subtypes to as much as 0.84 to 1.26 substitutions per synonymous site for the *giant*4a and *giant*5a subtypes (Fig. 6a; Table S8). A sliding window analysis of synonymous and nonsynonymous substitutions along the lengths of the *giant*1-3 genes revealed a few peaks with d*S* values up to 0.8 to 1.0 substitutions per synonymous site (Fig. 6b to d), whereas the high d*S* values of the *giants*4a/5a genes extended across the entire lengths of the genes, albeit with a drastic reduction at the C-terminal ends (Fig. 6e and g). The analyses confirmed that the d*S* values of *giants*4b/5b were close to zero throughout most of sequences with a few peaks of up to at the most 0.2 substitutions per synonymous site in the *giant*4b gene sequences (Fig. 6f and h).

**Fig. 7.**
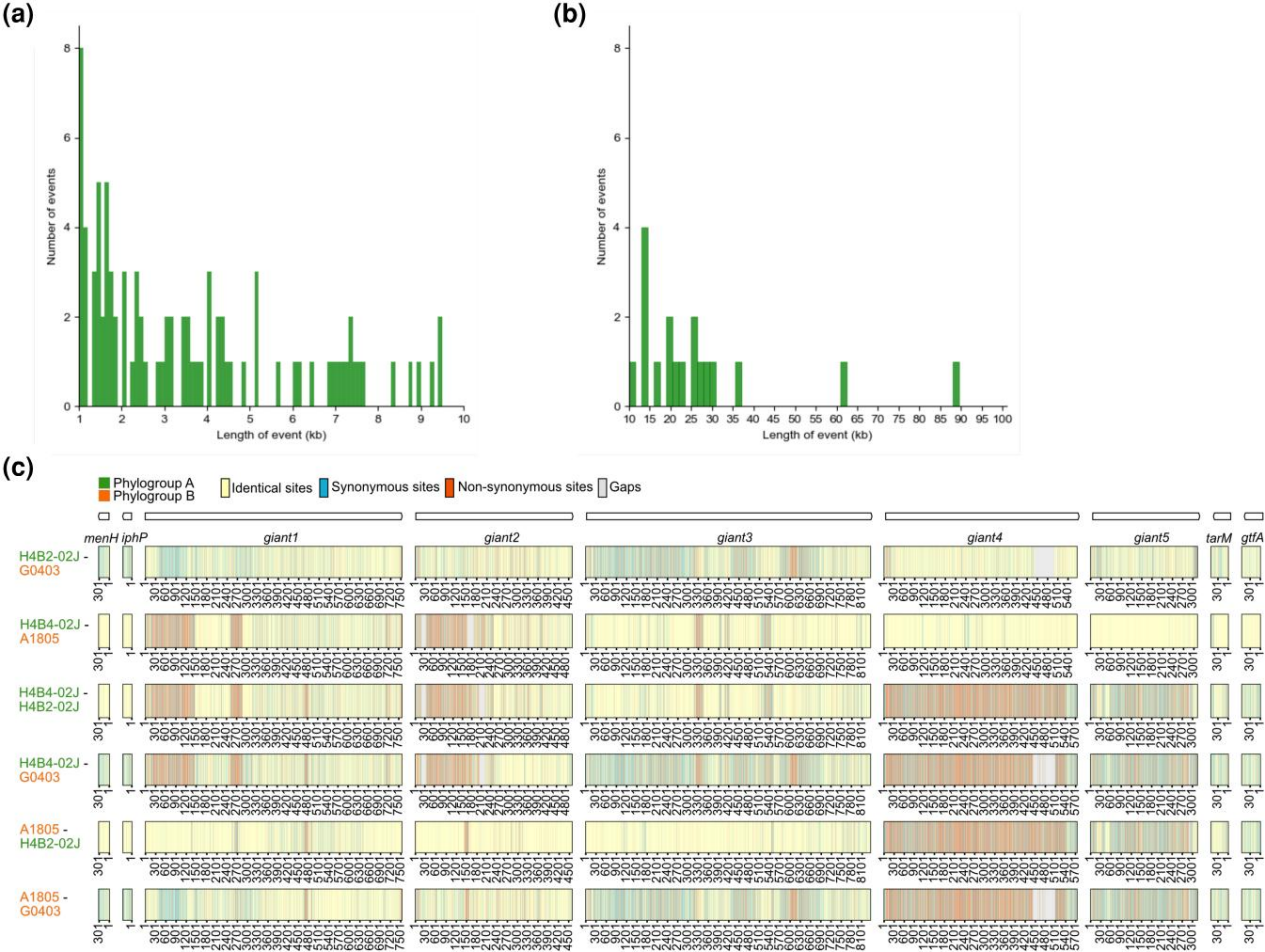
Predicted recombination events. Histogram showing the length distribution of the 106 predicted recombination tracts with determined breakpoint positions and supported by 3 or more methods in the spans a) 1 to 10 kb and b) 10 to 100 kb across a 200 kb genomic region that included the *giant*1-5 gene cluster. c) Example of the variability in SNPs at synonymous and nonsynonymous sites in *giant*1-5 gene cluster and its flanking genes in all pairwise comparisons of 3 strains from phylogroups A and B.

To examine the possible contribution of recombination events to the highly complex substitution patterns, we predicted putative recombination events in the phylogroup A-C strains across a 200 kb region that included the giant gene cluster. Due to the high sequence divergence of the first gene in the phylogroup A strain H1B1-04J, this strain was excluded from the analyses. In total, 405 recombination events were predicted in this genomic region by more than one of the methods RDP, Geneconv, and Maxchi (Table S9a). Of these, 106 events were further examined, which were longer than 1 kb, contained determined breakpoint positions, and were supported by more than 3 methods, also including Bootscan, Chimaera, and Seq3 (Table S9b). The median and mean length of tracts > 1 kb were 3.7 and 7.6 kb, respectively, but several recombination tracts of more than 25 kb that spanned across several giant genes were also predicted (Fig. 7a and b).

To visualize the positions of the inferred recombination tracts in each genome, they were mapped onto the 200 kb aligned region (Fig. S19). A plot of the breakpoint positions indicated a putative hotspot, which was located downstream of the giant gene cluster at position 171,631 to 171,883 in the alignment and reached above the 99% upper confidence interval (Fig. S20a; Table S10). Two and three additional hotspots were also indicated in the regions of parallel α-helical repeats in the *giant*1 and *giant*3 genes, respectively (Fig. S20b and d). However, no hotspots were identified immediately upstream of the *giant*1 gene or immediately downstream of the *giant*5 gene, although a single, long recombination tract of about 90 kb was predicted that extended across the whole giant gene cluster.

The extreme variability in sequence divergences across the giant genes, as shown by phylogenetic analyses, measures of observed substitution frequencies and predictions of recombination tracts, can be illustrated by an example taken from the comparison of 2 phylogroup A strains (H4B2-02J and H4B4-02J) with 2 phylogroup B strains (A1805 and G0403) (Fig. 7c). Despite being classified into different phylogroups, a comparison of strain H4B2-02J with strain A1805 revealed only a few SNPs at synonymous sites in the *giant*4b and *giant*5b genes. Likewise, relatively few SNPs were found in the *giant*4a and *giant*5a genes in the comparison of strain H4B2-02J with strain G0403. In contrast, a comparison of the phylogroup A strains H4B2-02J and H4B4-02J showed saturation of both synonymous substitutions and nonsynonymous substitutions over the *giant*4 gene, as did also a comparison of the phylogroup B strains A1805 and G0403. The dramatic difference in the *giant*4 and *giant*5 gene sequences between the two subtypes extended into the intergenic region located immediately upstream of the *giant*4 gene (Fig. S21). The example also revealed an increase in nonsynonymous substitutions at the 5′-end of the *giant*1 and *giant*2 genes in pairwise comparisons that included strain H4B4-02J.

## Discussion

The honeybee gut community is thought to serve many beneficial functions, such as for example in the defense against pathogens (Motta and Moran 2024). Host-beneficial functions often depend on traits that are strain-specific, such as the ability to synthesize and secrete antimicrobial peptides (Zendo et al. 2020; Dyrhage et al. 2022; Lang et al. 2023). Furthermore, many host-interaction processes in bacteria are mediated by large surface proteins, which are costly to make for the individual cell and gained and lost at high rates (Nogueira et al. 2009; Rankin et al. 2011). The allocation of as much as 6% of the genome in more than 100 *A. kunkeei* strains to multiple, consecutive genes for surface-exposed proteins of several thousand amino acids (Tamarit et al. 2015; Dyrhage et al. 2022) imply that they serve a very important function for the bacterial cell. However, our understanding of the rates and patterns at which very large surface proteins evolve is still fragmentary due to the lack of genomes from a sufficiently large number of closely related strains of the same species. In this study, we aimed to understand the phyletic distribution pattern of the very large surface proteins in *A. kunkeei* and determine the mechanisms and forces that drive their evolution. We also attempted to predict the structures of the giant proteins, thereby contributing to a broader understanding of surface-exposed large proteins in lactic acid bacteria.

### The Giant Proteins in *A. kunkeei* Have Long Elongated Structures with β-Solenoid Domains at Their N-Terminal Ends

The structural models for the Giant1, 2, and 3 proteins showed elongated, rod-like structures formed by parallel α-helical repeat domains that, to our knowledge, have not been described before. The estimated length of the Giant3 protein in extended conformation would be 0.2 µm, which is one seventh of the total length of 1.5 µm of the bacterial cell. In contrast to Giants1-3, the repeated region in the Giant4 protein contained an array of β-sheet domains, whereas Giant5 contained a very long, single α-helix.

The PAE plots showed confidence in the extended arrangement of the long α-helical repeat region of the Giant1-3 proteins, although the predictions did not provide information regarding the overall flexibility and relative positions of all protein domains. The elongated structures were confirmed when using sequences of about 600 to 1200 residues in length as inputs for the Giant1 predictions, although the longest input sequences of ca 1,900 residues resulted in several predicted hinge regions in the structure. However, the hinge regions were not consistently predicted when comparing different AlphaFold3 models, suggesting that these may be prediction artifacts. This tendency of AlphaFold to predict globular versions of less common structures, novel folds or elongated structures was previously observed for several proteins (Perrakis and Sixma 2021; Azzaz et al. 2022).

In contrast, the array of β-sheet domains constituting the major part of the Giant4 protein only showed confident predictions of the inter-domain arrangement for certain blocks of domains, suggesting a more flexible structure. The high PAE values in certain regions indicate the absence of co-evolution information between different parts of the structure. Similarly, the breaks in the single α-helix predicted in Giant5 may be a consequence of its elongated shape and absent co-evolution information for this type of structure. The long, single α-helix suggests that Giant5, in contrast to the other giant proteins, may not be monomeric, but form a di- or trimer stabilized through a coiled-coil structure. However, sequence-based coiled-coil prediction and multimeric AlphaFold prediction did not provide any consistent support for such models.

None of the 5 giant proteins in *A. kunkeei* were predicted to contain transmembrane domains, but all showed structural similarity to the SH3b-like domain at their C-terminal ends, which may mediate attachment to the bacterial cell wall. The positive ESP of the C-terminal end of all Giant1-5 proteins further supports attachment by this region to the negatively charged cell wall. Indeed, all 5 proteins were detected in the whole bacterial cell lysate as well as in the cell-free supernatant of the solution in which the cells of *A. kunkeei* strain A0901 had been cultivated (Seeger et al. 2023), indicating that the giant proteins may be loosely attached to the cell envelope.

Importantly, the predicted β-solenoid domain structures at the N-terminal end of the Giant1-4 proteins showed similarity to the experimentally determined structure of the β-solenoid domain in the SRRPs of *L. reuteri*, which are essential for biofilm formation and mediate binding to epithelial cells at neutral pH and to glycoproteins and plant-derived polysaccharides at low pH (Sequeira et al. 2018). The beta solenoid domains of Giant1-4 may have a similar function as in *L. reuteri*, but the low level of sequence identity, absent conservation of ligand-binding residues, and insertions in the Giant4 protein indicate that they recognize other ligands. Beyond the β-solenoid domains, the Giant proteins in *A. kunkeei* and the SRRPs in *L. reuteri* showed no significant similarity. Furthermore, the repeated sequences in the giant proteins in *A. kunkeei* were rich in alanine, whereas the repeats in the SRRPs were serine-rich. We also found no genes for a putative transport system near the giant gene cluster in any of the *A. kunkeei* strains, in contrast to the close proximity of genes for the SecA2-SecY2 transport system to the genes for SRRPs in other bacteria (Latousakis et al. 2020). Thus, none of the giant proteins contained all the typical characteristics of SRRPs, suggesting that they are not orthologs. Rather, we hypothesize that the Giant proteins and the SRRPs belong to two different families of adhesins that share some structural features, but serve different functions and bind different target molecules.

### The Giant1-3 Protein Family Appeared Early in the Evolution of Obligate Fructophilic Lactic Acid Bacteria

Interestingly, a gene for a member of the Giant1-3 protein family was identified in the genomes of 3 *Fructobacillus* species, which are only distantly related to *A. kunkeei* according to 16S rRNA phylogenies, as shown here and also in several previous publications (Konno et al. 2024; Pontes et al. 2024). A phylogenetic analysis based on the β-solenoid domains, on the other hand, showed that the homologs in *F. americanaquae* and *F. fructosus* clustered within the Giant1b clade, which contained proteins from *A. kunkeei* strains A1001 and A1404, *A. apinorum*, and *A. bombintestini*, whereas the homologs in all other *Apilactobacillus* species diverged outside the Giant1-3 clade. We consider it unlikely that the genome assemblies of the three *Fructobacillus* species contain contaminating contigs from *A. kunkeei* since the similarity extended only over the β-solenoid domain. Rather, we believe that the close relationship of the β-solenoid domains in very large surface proteins in some *Apilactobacillus* and *Fructobacillus* species is of biological significance.

*Fructobacillus* are environmental bacteria, but have also been cultured from honeybee food products (Takatani and Endo 2021) and detected in the adult honeybee gut (Moran et al. 2012). Indeed, most species of the genera *Apilactobacillus* and *Fructobacillus* are obligate fructophilic and grow well in the presence of fructose, but need electron acceptors such as oxygen for growth on glucose (Endo et al. 2009; Maeno et al. 2016). Their unique phenotypes are suggested to have been driven by convergent losses of more than 100 genes, of which the partial or complete loss of the *adhE* gene, which codes for a bifunctional alcohol/acetaldehyde dehydrogenase, was a key event in the transition to the obligate fructophilic lifestyle (Endo et al. 2014; Maeno et al. 2016, 2019). The acquisition of genes for very large surface proteins with β-solenoid domains may have been another such key event during the adaptation to the fructophilic lifestyle. Thus, we suggest that the giant proteins in *A. kunkeei* and their putative homologs in other species belong to a large family of adhesins with many paralogs that arose by duplication and divergence during adaptation to fructose-rich growth habitats.

The overlapping growth niches may have enabled exchanges of the β-solenoid domains both within and between *Apilactobacillus* and *Fructobacillus* species. For example, the 99.6% sequence identity of the β-solenoid domains in *F. fructosus* and *F. americanaquae*, 2 species that belong to 2 different phylogenetic groups within the *Fructobacillus* clade in the 16S rRNA tree, suggests that horizontal gene transfers are ongoing events within the genus *Fructobacillus*. Likewise, the clustering of some *Apilactobacillus* and *Fructobacillus* species in the Giant1b clade in the tree inferred from the β-solenoid domains might be due to horizontal gene transfer, but would imply that the rest of the genes have diverged extensively. If a transfer event did occur, the direction was most likely from *Apilactobacillus* to *Fructobacillus*, since the Giant1b subclade was embedded within a larger clade containing the Giant1-3 proteins from *A. kunkeei* and its closest relatives.

To our knowledge, gene transfers between species in *Apilactobacillus* and *Fructobacillus* have not yet been reported, and no plasmids have been found in *Fructobacillus* species (Mohamed et al. 2023). However, some *Fructobacillus* species show antagonistic activities against bee pathogens, such as *Paenibacillus* (Zeid et al. 2022), and it is tempting to speculate that these functions are plasmid-encoded, as in *A. kunkeei* (Dyrhage et al. 2022; Konno et al. 2024). Future studies of the mobilome of these 2 genera of fructophilic bacteria may shed light on possible routes for gene transfers. The identification of genes carried by the mobile elements in these species would also provide valuable information about traits of particular importance for the adaptation to fructose-rich growth niches.

### The Giant4-5 Protein Appeared Late in the Evolution of *Apilactobacillus*

In striking contrast to the Giant1-3 proteins, homologs to the Giant5 protein could only be found in *A. kunkeei*, *A. apinorum*, and *A. bombintestini*, and the Giant4 proteins contained multiple insertions in the β-solenoid domain not found in the Giant1-3 proteins, nor in any of the homologs in other species. This suggests that the last 2 genes were added to the cluster of giant genes at a later stage in evolution, perhaps during the transition to a host-associated lifestyle. Future studies of the binding targets of the atypical β-solenoid domain in the Giant4 protein and of the function of the Giant5 protein may help explain why these proteins show a more restricted phyletic distribution pattern.

### The Giant4-5 Proteins Have Co-Diverged in *A. kunkeei*

Another focus area of this study was to examine the mechanisms and selective forces that have driven the evolution of the giant proteins within the *A. kunkeei* population. To this end, we compared the genes for the Giant1-5 proteins in 33 *A. kunkeei* strains that represent the genetic diversity of strains sampled thus far. The phylogeny inferred from the full-length giant proteins in the *A. kunkeei* population showed that each of the Giant1-5 proteins were more similar to its positional homolog in the other strains (60% to 100% sequence identity) than to the proteins encoded by genes in the same cluster of a given strain (< 20% sequence identity), as also noted previously (Tamarit et al. 2015). Thus, although each genome contained multiple giant genes that may once have originated by a series of gene duplication events, there were no indications of recent gene conversion events between them.

The topologies of trees inferred from each of the individual Giant1-3 proteins were incongruent with each other, suggesting that the observed diversity does not simply reflect a gradual accumulation of nucleotide substitutions. On top of this, a few *A. kunkeei* strains contained highly diverged paralogs encoded by genes located at the upstream end of the gene cluster, and several more such genes are likely to be discovered in future sampling expeditions. In addition, multiple and diverse recombination events were predicted within the *giant*1-3 genes, consistent with a strong correlation between the observed synonymous and nonsynonymous rate variation within the genes.

The Giant4 and Giant5 proteins, on the other hand, showed a remarkably different diversification pattern. Unlike the Giant1-3 proteins, the single protein trees inferred from Giant4 and Giant5 proteins indicated that they have co-diverged, despite the lack of sequence similarity between them. The trees revealed the presence of 2 subtypes, here referred to as *a* and *b*, both of which contained *A. kunkeei* strains from phylogroups A to C (Dyrhage et al. 2022). Furthermore, the analyses showed that genes within subtype *b* were highly similar to each other with extremely low d*N* and d*S* values, indicative of very recent sequence homogenization by homologous recombination events that span across both genes. In contrast, a comparison of genes assigned subtype *a* showed that the d*N* and d*S* values were an order of magnitude higher, even in comparison of strains of the same phylogroup. We hypothesize that the 2 subtypes originated by duplication and divergence, although no single genome encodes both subtypes. The co-diversification pattern and the exceptionally low d*N* and d*S* values of the *giant*4b and *giant*5b genes might be due to ongoing sequence homogenization. We further hypothesize that the *giant*4a and *giant*5a gene variants have escaped the homogenizing effects of recombination and that the high d*S* values may approach the true frequencies of nucleotide substitutions, while strong purifying selection on the amino acid sequences explains the low d*N* values.

It may further be hypothesized that the 2 subtypes emerged under selection for sub-functionalization and that they serve different functions in the community and/or are expressed under different growth conditions. The sequence divergence of the 2 subtypes extends into the intergenic region upstream of the *giant*4 gene, consistent with the finding that the 5′-end of the long recombination events are located at the C-terminal end of the *giant*3 gene, which raises the possibility that the 2 subtypes are expressed from different promoters. In addition, the *A. kunkeei* strains A1202 and H1B3-02M contain IS3 transposons in the intergenic region between the *giant*3 and the *giant*4 genes, which might also affect the expression patterns of the downstream *giant*4 and *giant*5 genes. The previous study of the *A. kunkeei* extracellular proteome (Seeger et al. 2023) was performed in strains A0901 and A1401, both of which contained highly similar *giant*4-5b genes. A more systematic analysis of the functions and expression patterns of the 2 subtypes of the *giant*4 and *giant*5 genes will show whether the co-divergence pattern is due to selection for sub-functionalization or if it is attributed to neutral processes alone.

## Concluding Remarks

This study provides insights into the structures and evolution of very large surface proteins in obligate fructophilic lactic acid bacteria. Homologs to the first 3 genes in the gene cluster, which code for very large proteins with β-solenoid domains in *A. kunkeei*, were identified in both closely and distantly related fructophilic bacteria, whereas homologs to proteins encoded by the last 2 genes in the gene cluster were only detected in *A. kunkeei* and 2 of its closest relatives. While our work is consistent with previous studies showing that genes for extracellular surface proteins evolve rapidly (Nogueira et al. 2009), the results have also provided insights into the very complex interplay between recombination and single-nucleotide mutations that generate diversity of surface proteins within a bacterial population. Future studies on the regulation, function, and binding targets of this fascinating family of very large surface proteins could shed light on the steps involved in the bacterial adaptation to and colonization of honeybees and other fructophilic growth habitats.

## Materials and Methods

### PCR and Sequencing

Primers for PCR were designed using the Primer3Plus tool (Koressaar and Remm 2007; Untergasser et al. 2012). The Phusion Hot Start II DNA Polymerase (2 U/µL) (cat no: F549S, ThermoFisher) was used. Annealing temperature gradients were tested for each primer pair. The PCR products were visualized on 1% Agarose gel before Sanger Sequencing (Mix2Seq kit, Eurofins Genomics), in both forward and reverse directions using the PCR primers.

### Retrieval and Selection of *A. kunkeei* Genomes

The *A. kunkeei* genome sequences were downloaded from NCBI on 2022-12-08 and 2022-12-12. The genome sequence file of strain MP2 was changed into its reverse complement prior to the analyses. For groups of strains with an average nucleotide sequence identity value (ANI) of 99.9% (Dyrhage et al. 2022), only one representative strain was selected.

### Predictions of Protein Architectures and 3D Structures

The giant proteins in *A. kunkeei* strain A0901 (A0901_02220 to A0901_02260) were selected for studies of protein features and for protein structure predictions. SignalP 6.0 was used for prediction of signal peptides (mode: slow) (Nielsen et al. 2019), DeepTMHMM 1.0.24 for predictions of transmembrane regions (Hallgren et al. 2022), and InterPro 97 for domain searches (Paysan-Lafosse et al. 2023). The RADAR 1.1.1 tool was used to characterize sequence repeats, of which the most highly supported internal segment repeats were considered for analysis (Madeira et al. 2022). For the analyses of amino acid utilization, the ProtParam tool was used with a custom script (Gasteiger et al. 2005). Pairwise protein identity values (using flag -sprotein TRUE) were calculated using the EMBOSS Needle program in a custom script (Rice et al. 2000; Cock et al. 2009).

AlphaFold3.0.0 was used for structure predictions, running 5 seeds per model (Abramson et al. 2024). Due to the lengths of the internal repeat segments in Giant1-4, the N- and C-terminal structures were predicted separately, and different input lengths of the repeat regions were tested. For Giant1, the repeat region was divided into 3, 5, 7, 9, and 11 input fragments, reaching between approximately 600 to 2000 residues in length, with about 60 amino acids overlap. For Giant2-5, 2 different input lengths were tested. The best model was selected and visualized in UCSF ChimeraX 1.10.1 (Meng et al. 2023). A script for renumbering amino acid residues in the PDB files was obtained from http://www.canoz.com/sdh/renumberpdbchain.pl. Each individual fragment was loaded and merged into a full-length model by specifically aligning the overlapping residues using the “Matchmaker” function (with options “Best-aligning pair of chains between reference and match structure” and “Also restrict to selection”). The structures were colored according to pLDDT. The Coulombic electrostatic potential was calculated for Giant1-5 (with command “coulombic protein key true”). PAE plots were generated using a modified version of a script obtained from https://davinci.icm.uu.se/notes/alphafold.html.

### Structure and Sequence-Based Homology Searches

Putative domains predicted by AlphaFold3 of proteins in *A. kunkeei* strain A0901 (A0901_02220-A0901_02260) were used for structural homology searches in Foldseek (van Kempen et al. 2024). For some of the hits, structural alignments were downloaded including the TM-Score, RMSD, and *E*-value as provided by Foldseek. The amino acid sequence of the β-solenoid domains of proteins in *A. kunkeei* strains A0901, A1001, and A1401 (A0901_02220-A0901_02250; A1001_02170-A1001_02180; A1001_02210-A1001_02220 and A1404_02370-A1404_02390) was used as a query in homology searches against the NR database using BLASTP (Altschul et al. 1990). The full-length protein encoded by the *giant*5 gene in *A. kunkeei* strain A0901 (A0901_02260) was used as the query in homology searches using BLASTP against each of the individual proteomes of the species included in the 16S rRNA phylogeny. Hits to experimentally determined structures with *E*-values < 0.01 in the structural homology search and hits with *E*-values < 1e^−10^ in the sequence-based homology search were selected for further analyses, excluding hits to *A. kunkeei*.

### Alignment and Phylogenies

Protein and 16S rRNA sequences were aligned using MAFFT L-INS-i (v7.490) (with option “–localpair –maxiterate 1000”) (Katoh et al. 2002; Katoh and Standley 2013). Genomic regions and whole genomes were aligned in a pairwise manner using BLASTN from the blast-2.13.0 package using a custom script (with parameters: -perc_identity 0, -evalue 1e^−3^, -max_target_seqs 1,000, and -word_size 7 for the genomes and contig alignments of species other than *A. kunkeei*). The *A. kunkeei* genome alignments and the alignments of the genomes and contigs of all other species were visualized using the pyGenomeViz packages v0.3.2 and v1.4.2, respectively (Shimoyama 2022, 2024).

Maximum likelihood phylogenetic analyses inferred from the β-solenoid domains of the Giant1-4 proteins in *A. kunkeei* and their homologs in other species were carried out using IQ TREE 1.6.12 (Nguyen et al. 2015) and WAG + F + R5 as the optimal substitution model according to BIC (Kalyaanamoorthy et al. 2017). For the tree inferred from the β-solenoid domains in the Giant1-3 proteins in *A. kunkeei*, LG + F + R5 was the optimal substitution model according to BIC. For the single giant protein trees, ModelFinder suggested WAG + F + R3, WAG + F + I + G4, JTT + F + R6, WAG + F + R3, and JTTDCMut + F + G4 according to BIC for Giant1, 2, 3, 4, and 5, respectively (Kalyaanamoorthy et al. 2017). For the 16S rRNA tree, ModelFinder suggested TIM3e + R3 as the substitution model according to BIC (Kalyaanamoorthy et al. 2017). The nonparametric UltraFast Bootstrap method was used to calculate node supports (Hoang et al. 2018), and the SH-aLRT branch test was performed with 1,000 replicates. The phylogenies were visualized using Figtree v1.4.4 from http://tree.bio.ed.ac.uk/software/figtree/ and displayed as midpoint rooted. For topology analyses, the giant protein phylogenies were compared in Dendroscope, version 3.8.4 (Huson and Scornavacca 2012). Tanglegrams were displayed as rectangular phylograms.

### Analysis of Nucleotide Substitution Frequencies

The giant protein alignments were back-converted to nucleotide codon alignments with the PAL2NAL program v14.1 (Suyama et al. 2006). All gaps were removed (with option “-output paml -nogap”). Pairwise substitution frequencies were estimated per gene and strain pair using CodeML from the PAML package v4.9 (with options: CodonFreq = 2 (the F3X4 model), model = 1 (which allows for one omega ratio per branch) (Yang 2007). A script for parsing the substitution data was obtained from https://github.com/faylward/dnds/blob/master/parse_codeml_output.py and manually modified. For the sliding window analysis, CodeML was used with the same parameters as above, and the input sub-codon alignments were created using custom scripts generated with aid of ChatGPT at https://openai.com/index/chatgpt/(with parameters: window size = 450 and step size = 21). The d*N* and d*S* values at the window midpoint position were plotted using a lineplot in Seaborn (Waskom 2021). The pairwise d*S* values and SNPs were plotted using boxplot and displayed using the heatmap function, respectively, in Seaborn (Waskom 2021).

### Predictions of Recombination Tracts

The RDP5 program (version 5.23) was used for analysis of recombination (Martin et al. 2021). The genomic region was aligned using MAFFT-FFT-NS-i (v7.490) (with options “–retree 2 –maxiterate 1000”) (Katoh et al. 2002; Katoh and Standley 2013), and the *giant*1-5 gene regions were manually exchanged with back-translated, codon-correct alignments using the PAL2NAL option “-output fasta” (Suyama et al. 2006). In the RDP5 settings, linear sequences were selected; all other settings were default. In the secondary scan, only recombination events supported by >3 methods (including RDP, Geneconv, BootScan, MaxChi, Chimaera, and 3Seq) that were longer than 1,000 nucleotides and with determined breakpoint positions were accepted (Smith 1992; Padidam et al. 1999; Martin and Rybicki 2000; Posada and Crandall 2001; Martin et al. 2005; Lam et al. 2018). The number and length of accepted events were plotted using a custom script for displot in Seaborn (Waskom 2021). Breakpoint distribution plots were generated for the accepted events also from a custom script (Heath et al. 2006).

## Supplementary Material

evag011_Supplementary_Data

## Data Availability

Additional datasets and custom scripts used in this project are available at the SciLifeLab Data Repository, https://doi.org/10.17044/scilifelab.c.7706651.
