# Supplementary material for: Origin and Evolution of Very Large Extracellular Proteins in Fructophilic Lactic Acid Bacteria
Source: Genome Biol Evol. 2026 Jan 22;18(2):evag011. doi: 10.1093/gbe/evag011 (PMC12863079; doi:10.1093/gbe/evag011)

SUPPLEMENTARY FIGURE AND TABLE LEGENDS

Origin and Evolution of Very Large Extracellular Proteins in Fructophilic Lactic Acid Bacteria

Julia E. Pedersen, Marina Mota-Merlo, Andrea Garcia-Montaner, Maria Selmer, and Siv G. E. Andersson

SUPPLEMENTARY FIGURE LEGENDS

Figure S1. AlphaFold3 model (scale bar 100 Å, color coded according to pLDDT) and predicted aligned error (PAE) plots of the Giant1 α-helical repeat region divided into (A) eleven input fragments, (B) nine input fragments, (C) seven input fragments, (D) five input fragments and (E) three input fragments.

Figure S2. AlphaFold3 model (scale bar 100 Å, color coded according to pLDDT) and PAE plots of (A) the complete Giant2 protein, with the α-helical repeat region divided into five input fragments, and of (B) the α-helical repeat region, predicted from one complete input fragment.

Figure S3. AlphaFold3 model (scale bar 100 Å, color coded according to pLDDT) and PAE plots of (A) the complete Giant3 protein, with the α-helical repeat region divided into 13 input fragments, and of (B) the α-helical repeat region, predicted from three input fragments. Structural alignments of (C) Giant1 NTD1 (red) and the corresponding domains in Giant2 (green) and Giant3 (blue), and (D) Giant1 NTD3 (red) and the corresponding domains in Giant2 (green) and Giant3 (blue).

Figure S4. AlphaFold3 model (scale bar 10 Å, color coded according to pLDDT) and PAE-plots of Giant4, divided into shorter fragments spanning (A) the N-terminal domains NTD1-4, (B-C) across the β-sheet array and (D) the C-terminal domains including CTD1-4 and a structural alignment of the Giant4 CTD1-4 (red, green, blue and yellow, respectively) and the Giant1 CTD4 domain (white). (E) AlphaFold3 model of the β-sheet array divided into two input fragments (scale bar 100 Å, color coded according to pLDDT).

Figure S5. AlphaFold3 model (scale bar 10 Å, color coded according to pLDDT) and PAE plots of Giant5, displaying (A) the N-terminal domains NTD1-9, followed by a single α-helix reaching until (B) the C-terminal domains CTD1-3, including a structural alignment of the Giant5 CTD1 and CTD3 (in red and green, respectively) and the Giant1 CTD4 (blue) domain. (C) The single α-helix model predicted between the N- and C-terminal regions displayed in A-B (scale bar 100 Å, color coded according to pLDDT), and (D) the complete Giant5 protein predicted from two input fragments (scale bar 100 Å, color coded according to pLDDT).

Figure S6. (A-E) Displaying the Coulombic electrostatic potential (ESP) of Giant1-5, respectively (scale bar 100 Å). The protein surfaces are colored according to potential, where red displays negative potential and blue displays positive potential.

Figure S7. MAFFT L-INS-i alignment of the β-solenoid domain in Giant1-4 in strain A0901, and the 5ny0 and 5nxk *L. reuteri* sequences from strains 100-23 and 53608, respectively. Surface exposed residues previously characterized to be involved in TGA binding by Sequeira et al. (2018) are marked by asterisk and red column in alignment (residues from strain 100-23 in parenthesis): W375 (377), K377, Y425 (428), W450 (453), Y482, K485 (488), D487 (490), R512 (515) and R543 (546).

Figure S8. Structural alignment of giant protein domains and Foldseek search hits, (A) the Giant3 NTD3 domain and PDB ID: 5ze8, (B) the Giant3 NTD3 domain and PDB ID: 5ze8, (C) the Giant1 CTD2 domain and PDB ID: 7qeh, (D) the Giant5 CTD1 domain and PDB ID: 8a0m (E) the Giant2 CTD1-2 domains and PDB ID: 8a0m, (F) the Giant2 CTD1-2 domains and PDB ID: 7qeh and (G) the Giant5 CTD1-3 domains and PDB ID: 7qeh, displaying region matches as well as full query and target sequences.

Figure S9. A 16S rRNA phylogeny inferred from a selected set of species that yielded BlastP hits to the β-solenoid domains in the Giant proteins of *A. kunkeei*, with nodes supported by 1000 ultrafast bootstraps and SH-like pseudoreplicates. Only values above 75% are shown. The tree was midpoint rooted.

Figure S10. Gene synteny plot of genomic regions that contain genes encoding proteins that yielded BlastP hits to the β-solenoid domains of the Giant1-3 proteins (colored in light blue) and the Giant5 protein (colored in light green). The giant genes in *A. kunkeei* are shown in black color and transposons in red color. Sequence numbers (0-3) above the genes indicate the number of the giant gene in *A. kunkeei* (giant0-3) that yielded a hit to the gene with an E-value of < 1e-90.

Figure S11. (A and B) Comparison of the genomic region that contain the giant genes in *A. kunkeei* with genomic regions in a selected set of genomes that contain one or more genes for putative homologs. The *A. kunkeei* giants are colored in black and transposons in red. Genes encoding proteins that yielded BlastP hits to the β-solenoid domains of the Giant1-3 proteins are colored in light blue and to the Giant5 protein are colored in light green. Pairwise nucleotide blastn similarities are displayed as grey vertical lines and the color intensity represents the similarity, with the minimum identity level set to 50%. Inversions are marked

in red. The order of genomes from top to bottom follows the 16S rRNA tree, but is changed in (B) to show the similarity between *F. americanaquae* to the *A. bombintestini*. (C) Alignment of the giant protein encoded by *A. kunkeei* A1001_02210 with proteins from *Fructobacillus* species that contain β-solenoid domains. The region of the protein alignment that contain the β-solenoid domain is marked with a red box.

Figure S12. Phylogeny inferred from the β-solenoid domain sequences of all proteins that yielded BlastP hits to the β-solenoid domains of the Giant proteins in *A. kunkeei* with E-value <1e-10. The numbers at the nodes show the statistical support as indicated by 1000 ultrafast bootstraps and 1000 SH-like pseudoreplicates. The clades containing the Giant1-4 proteins in *A. kunkeei* are color coded. The tree has been midpoint rooted.

Figure S13. Phylogeny inferred from the β-solenoid domain sequences of proteins for which a hit was obtained to the β-solenoid domains in the *A. kunkeei* Giant1-3 proteins in BlastP searches, after filtering out sequences that clustered with or basal to the Giant4 clade, were <1000 amino acids long or were more than 99.5% 16S rRNA identical to an already selected taxon *A. kunkeei*, *A. apinorum* (Aap) and *A. bombintestini* (Abo) are marked in light green; *Fructobacillus* and *Leuconostoc* species (Ffr, Fam, Fps and Lci) are marked in light blue and all other species in violet. The numbers 0-3 refer to the Giant0-3 proteins in *A. kunkeei*. The two sister clades of the β-solenoid domains in the Giant1 clade have been named 1a and 1b. The taxa descriptions include species abbreviations or *A. kunkeei* strain designations, protein accession numbers or *A. kunkeei* locus tags and the position of the β-solenoid domain sequence in the protein used for the phylogeny. The numbers at the nodes of the tree show the statistical support as indicated by 1000 ultrafast bootstraps and 1000 SH-like pseudoreplicates. Only values above 75% are shown. The tree has been midpoint rooted.

Figure S14. (A) Gel image of PCR products in the region of the third giant gene in strain H3B2-09X. Sanger sequencing results for (B) forward and (C) reverse primer, region of interest marked with red square. (D) Schematic representation of Pacbio genomic sequence and verified Sanger sequenced PCR product.

Figure S15. (A) Gel image of PCR products in the region of the third giant gene in strain H4B5-04J. Sanger sequencing results for (B) forward and (C) reverse primer, region of interest marked with red square. (D) Schematic representation of Pacbio genomic sequence and verified Sanger sequenced PCR product.

Figure S16. The genomic region of the giant gene cluster (blue), including up- and downstream regions (non-giant genes in white, transposons in yellow), is visualized in phylogenetic order. Strain names are color-coded according to phylogroup. Pairwise nucleotide blastn results are displayed as grey vertical lines, where color intensity represents similarity with the minimum identity set to 50% and inversions are marked red.

Figure S17. Phylogenies inferred from the (A) Giant1, (B) Giant2, (C) Giant3, (D) Giant4 and (E) Giant 5 proteins in *A. kunkeei*. The taxa descriptions include *A. kunkeei* strain designations and locus tags. Strain names are color-coded according to phylogroup. The numbers at the nodes of the tree show the statistical support as indicated by 1000 ultrafast bootstraps and 1000 SH-like pseudoreplicates.

Figure S18. Rectangular phylograms comparing the (A) Giant1 to Giant2, (B) Giant1 to Giant3, (C) Giant1 to Giant4, (D) Giant2 to Giant3, (E) Giant2 to Giant4 and (F) Giant3 to Giant4. The taxa descriptions include *A. kunkeei* strain designations and locus tags. Strain names are color-coded according to phylogroup. Circles at nodes refer to the statistical support inferred from 1000 ultrafast bootstraps and 1000 SH-like pseudoreplicates. Nodes with ≥ 95% support from both methods are highlighted with grey circles and nodes with 100% support from both methods with black circles.

Figure S19. Schematic view of recombination events exported from RDP5, accepted events are in color with red frame and rejected events are in grey. Events are colored according to P-value. The positions of the giant1-5 genes within the 200 kb genomic segment are marked with vertical segments colored in purple, red, yellow, green and orange, respectively.

Figure S20. RDP5 sliding window analysis of breakpoint distributions, displayed (A) along the entire 200 kb genomic segment, as well as close ups of the (B) giant1, (C) giant2, (D) giant3, (E) giant4 and (F) giant5 regions, where domain information from the AlphaFold3 predictions is included (bottom) and possible recombination hot spots are marked (red arrows).

Figure S21. Nucleotide sequence alignment of the intergenic region between the giant3 and giant4 genes in *A. kunkeei* strains.

SUPPLEMENTARY TABLE LEGENDS

Table S1. Results from SignalP, DeepTMHMM, and Interpro predictions in strain A0901 Giant1-5 proteins.

Table S2. Amino acid utilization analysis in strain A0901 Giant1-5 proteins, listing (A) the amino acid content for each complete giant protein, (B) the average amino acid content for all proteins in strain A0901, (C) the amino acid content in the AlphaFold3 predicted domains in Giant1-5 and (D) the amino acid content in the L. reuteri strain 53608 SRRP protein. (E) Sequence information for the L. reuteri strain 53608 SRRP protein, listing genbank accession, strain name, genbank download date, assigned gene name, locus tag, protein accession number, location, orientation, assigned ID, and comments.

Table S3. (A) Residue positions included in sequence fragments submitted for AlphaFold3 prediction. (B) Average pLDDT for each domain or repeat region predicted by AlphaFold3.

Table S4. (A) Information on the Foldseek PDB search hits, using the β-solenoid domain as query sequence, namely the PDB structures of the L. reuteri SRRP β-solenoid domain: PDB ID, species, host, strain name, reference and the assigned ID. (B) Lists blastp hits with E-value ≤1e-10 with the following information: blastp protein ID, strain, species, host/isolation source, assembly, genbank accession, location, orientation, sequencing technology, genome size, assembly level/number of chromosomes or contigs, publication (PMID), protein length, blastp date, query sequence, query coverage (%), query identity (%), E-value, assigned name and notes. (C) Lists information on the 16S rRNA sequences used, including: genbank accession, species, strain name, sequence download date, locus tag, pairwise identity to AKUA0901_00790 (%), assigned name in the supplementary figures and files, assigned name in the figure and sequencing technology. (D) Lists filtered blastp hits from Table S4A, filtering out: all strains but one per species, sequences <1000 amino acids, sequences clustering with or basal to the Giant4 sequences and species with >99.5% 16S rRNA identities. Also includes column with query identity (%) between full length sequences. (E) Lists the blastp hits using the complete Giant5 protein as query, including: blastp protein ID, locus tag, strain, species, host/isolation source, genbank accession, chromosome or contig, location, orientation, protein length, blastp date, query sequence, query coverage (%), query identity (%), E-value, assigned name, cluster organization, AlphaFold3 models and notes.

Table S5. (A) Lists information about the 104 genome assemblies used, including genbank accession number, strain name, genbank download date and comments. (B) Information

regarding the phylogroup A-C and (C) E-F giant sequences is listed, including: strain name, assigned gene name, locus tag, protein accession number, location, orientation, assigned ID and comments. The 33 reference strains included in the analysis are marked under the comments column and displayed in bold.

Table S6. PCR primer information, including assigned primer name, direction, primer sequence, length (bp), amplicon length (bp), Tm (°C), GC (%), target, annealing temperature and Mix2Seq kit ID.

Table S7. Pairwise protein identities listed for all giant sequences (which are named according to "Assigned ID" column in Table S5B-C), organized according to (A) phylogroup A-C, (B) phylogroup E-F and (C) the H1B1-04J_02160 sequence comparisons. All sequences are color-coded according to phylogroup.

Table S8. Matrices of codeML pairwise substitution frequencies. (A) Lists the mean and median dN and dS for Giant1-5. (B-J) The dS and dN values estimated for all pairwise strain comparisons of the giant1-5 genes.

Table S9. (A) Raw output from the RDP5 preliminary scan and (B) accepted events according to the following criteria: the event was supported by >3 methods, it was longer than 1000 nt and had determined breakpoints.

Table S10. RDP5 sliding window breakpoint distribution plot data.

REFERENCES

Cifuente JO et al. 2023. A multi-enzyme machine polymerizes the Haemophilus influenzae type b capsule. Nat Chem Biol. 19:865–877. doi: 10.1038/s41589-023-01324-3.

Sequeira S et al. 2018. Structural basis for the role of serine-rich repeat proteins from Lactobacillus reuteri in gut microbe–host interactions. Proc Natl Acad Sci U S A. 115:E2706–E2715. doi: 10.1073/pnas.1715016115.

Figure S1

A

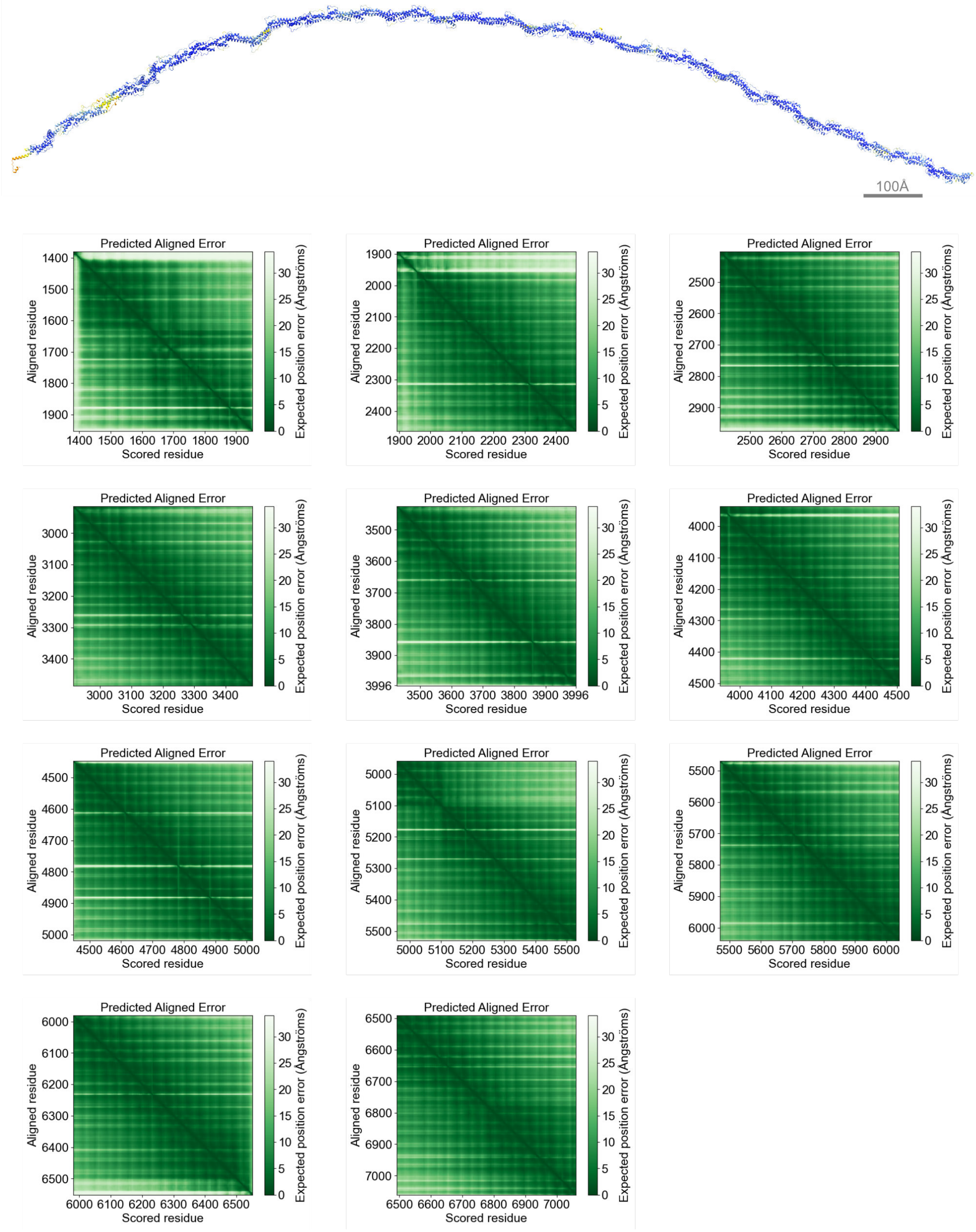
100Å
Predicted Aligned Error
Aligned residue
Scored residue
Expected position error (Ångströms)

Figure S1

B

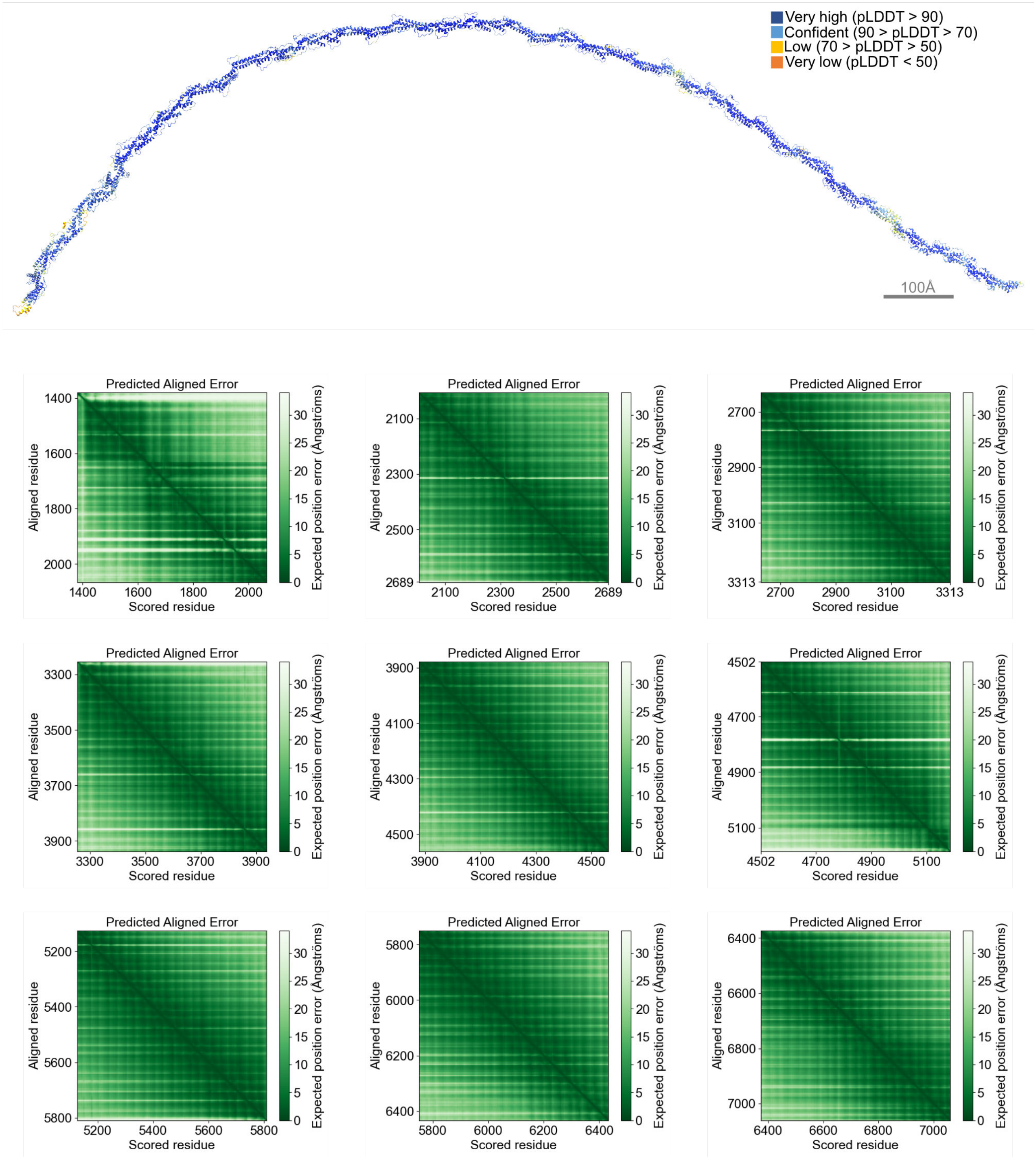

Very high (pLDDT > 90)
Confident (90 > pLDDT > 70)
Low (70 > pLDDT > 50)
Very low (pLDDT < 50)
100Å
Predicted Aligned Error
Aligned residue
Scored residue
Expected position error (Ångströms)
0
5
10
15
20
25
30
1400
1600
1800
2000
2100
2300
2500
2689
2700
2900
3100
3313
3300
3500
3700
3900
4100
4300
4500
4502
4700
4900
5100
5200
5400
5600
5800
6000
6200
6400
6600
6800
7000

Figure S1

C

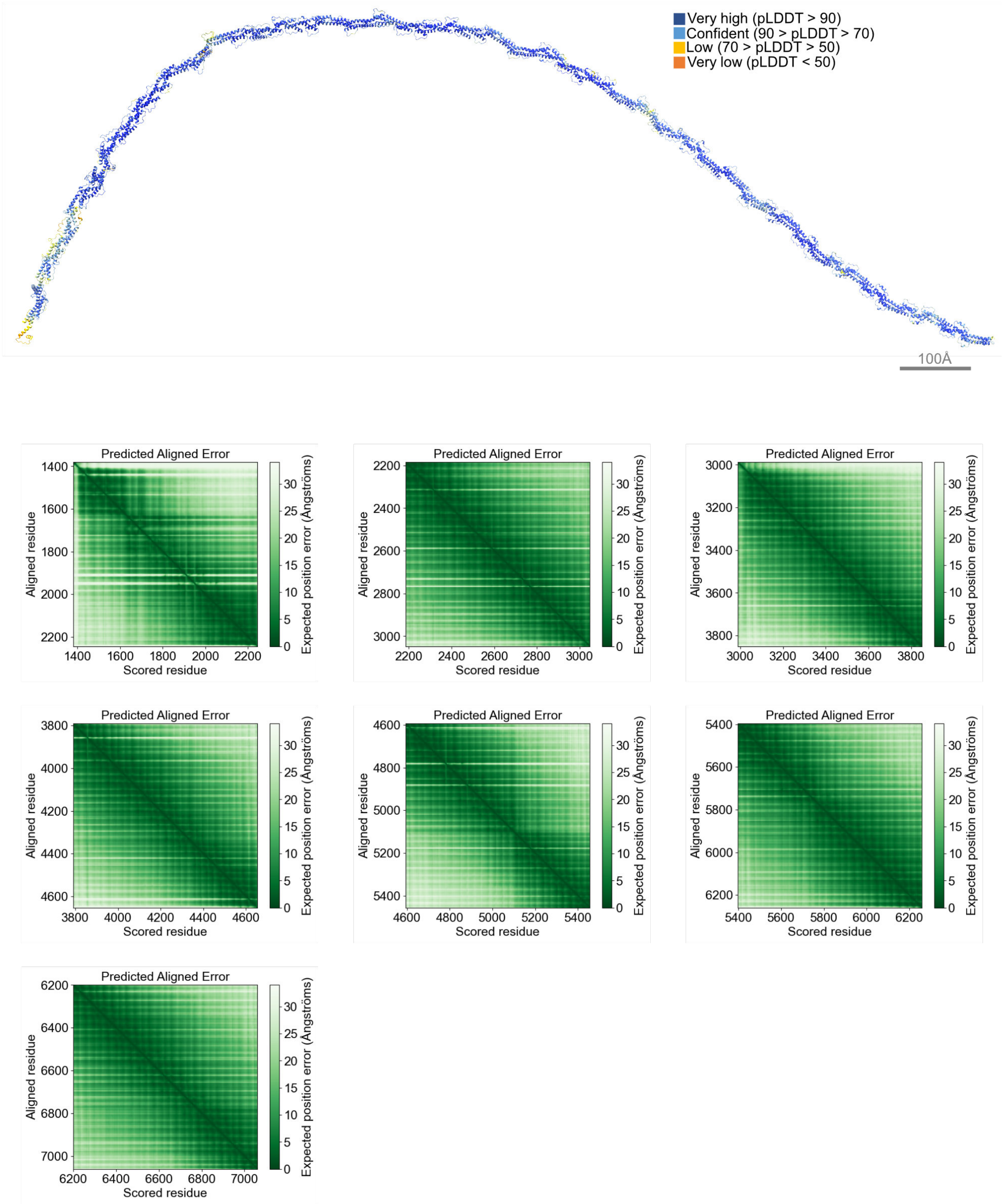
Very high (pLDDT > 90)
Confident (90 > pLDDT > 70)
Low (70 > pLDDT > 50)
Very low (pLDDT < 50)
100Å
Predicted Aligned Error
Aligned residue
Scored residue
Expected position error (Ångströms)

Figure S1

D

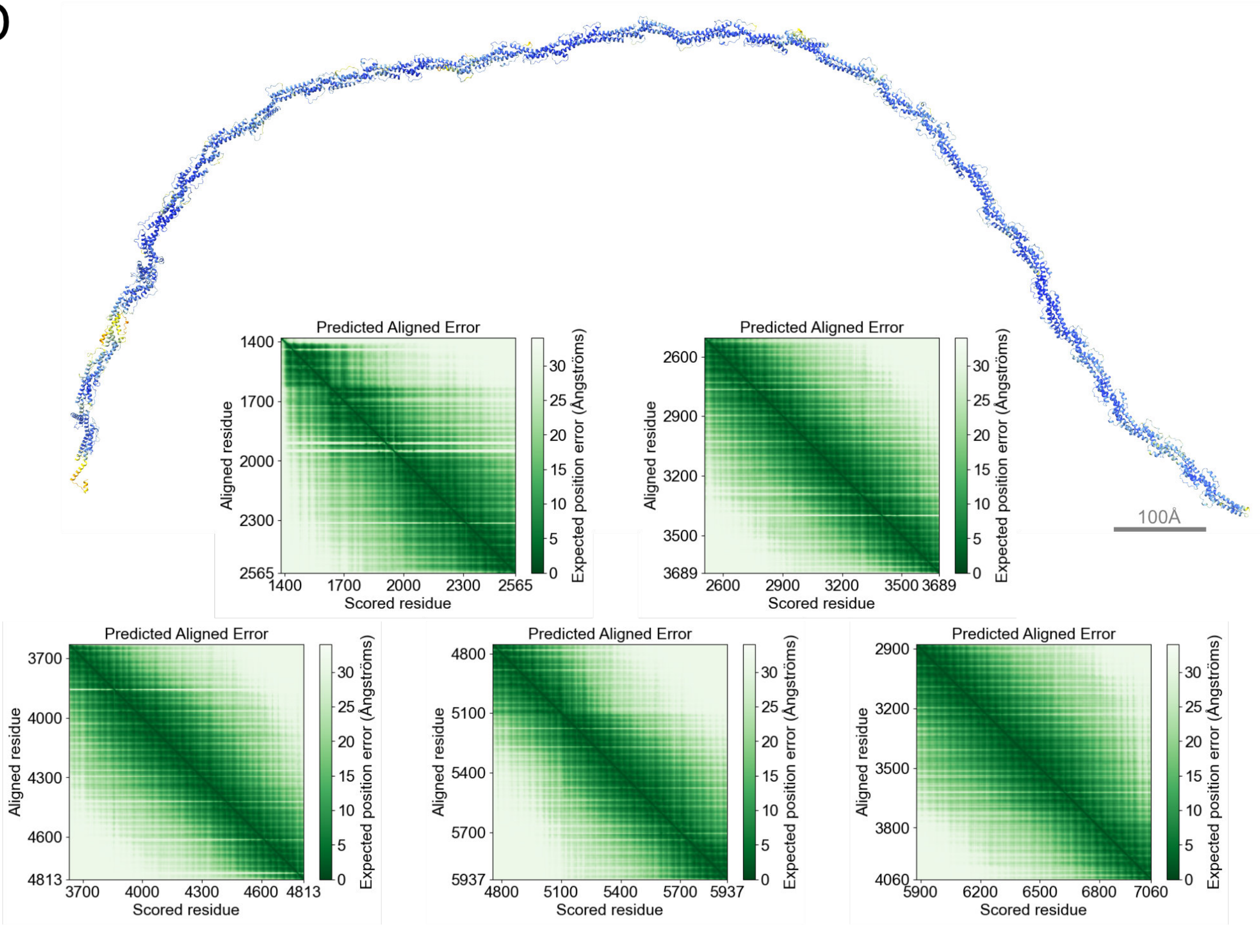

100Å
Predicted Aligned Error
Aligned residue
Scored residue
Expected position error (Ångströms)
1400 1700 2000 2300 2565
2600 2900 3200 3500 3689
3700 4000 4300 4600 4813
4800 5100 5400 5700 5937
2900 3200 3500 3800 4060
5900 6200 6500 6800 7060
0 5 10 15 20 25 30

E

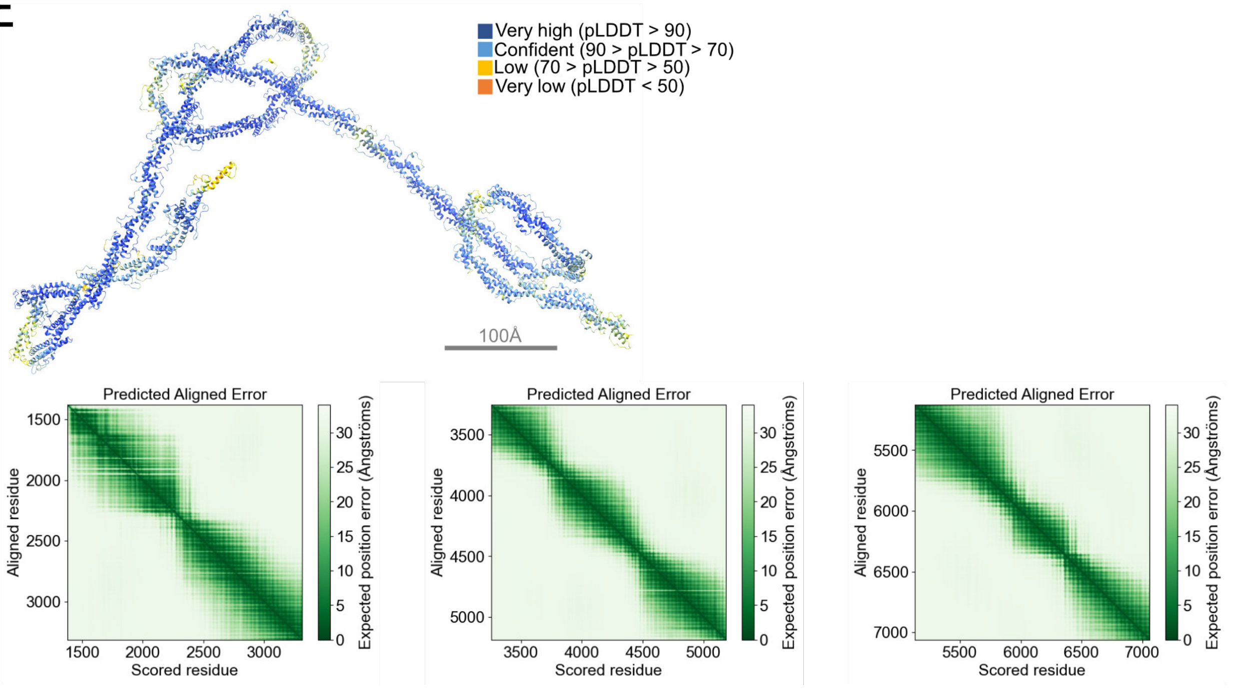

Very high (pLDDT > 90)
Confident (90 > pLDDT > 70)
Low (70 > pLDDT > 50)
Very low (pLDDT < 50)
100Å
Predicted Aligned Error
Aligned residue
Scored residue
Expected position error (Ångströms)
1500 2000 2500 3000
3500 4000 4500 5000
5500 6000 6500 7000
0 5 10 15 20 25 30

Figure S2

A

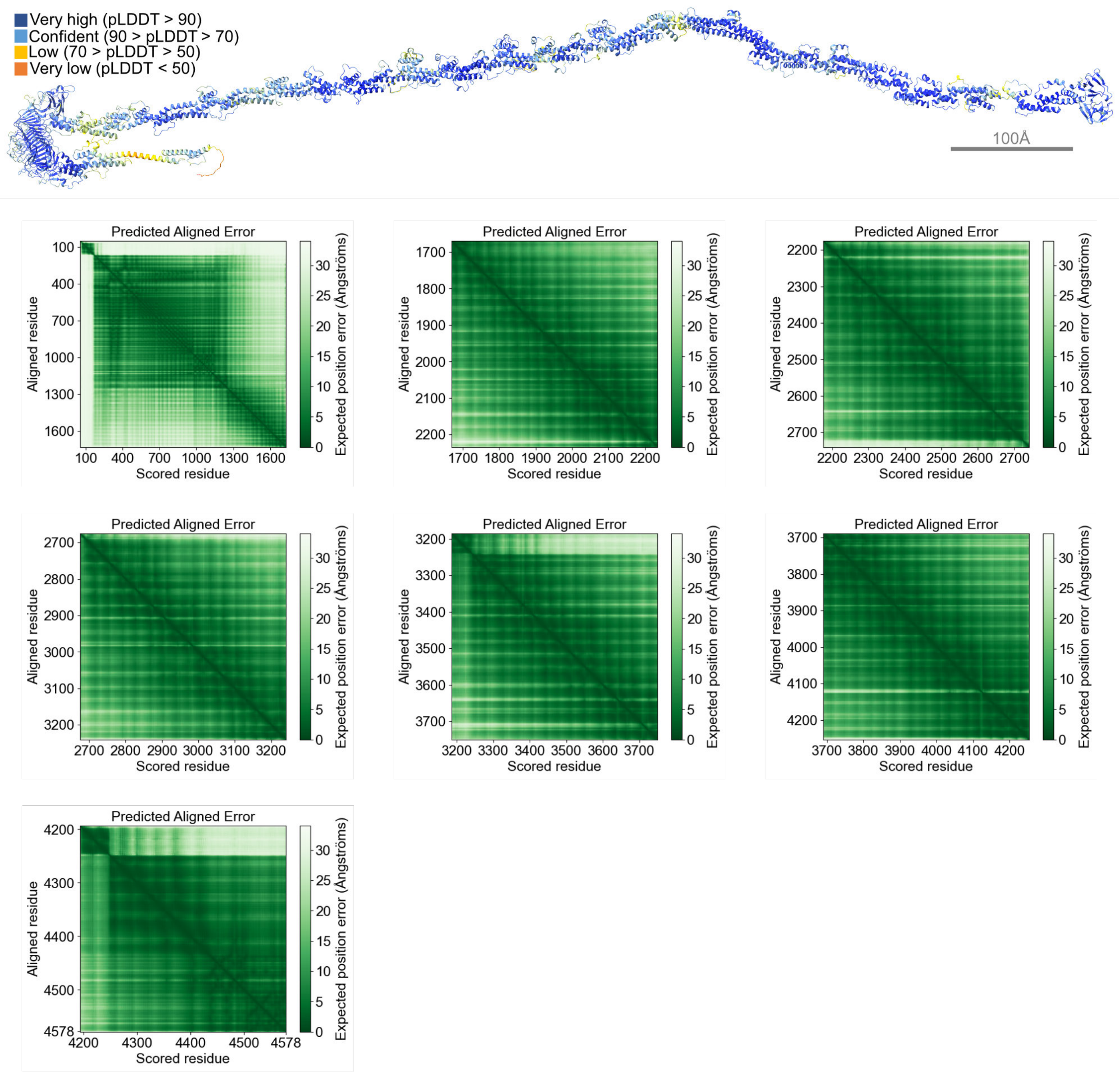
Very high (pLDDT > 90)
Confident (90 > pLDDT > 70)
Low (70 > pLDDT > 50)
Very low (pLDDT < 50)
100Å
Predicted Aligned Error
Aligned residue
Scored residue
Expected position error (Ångströms)
100 400 700 1000 1300 1600
1700 1800 1900 2000 2100 2200
2200 2300 2400 2500 2600 2700
2700 2800 2900 3000 3100 3200
3200 3300 3400 3500 3600 3700
3700 3800 3900 4000 4100 4200
4200 4300 4400 4500 4578
0 5 10 15 20 25 30

B

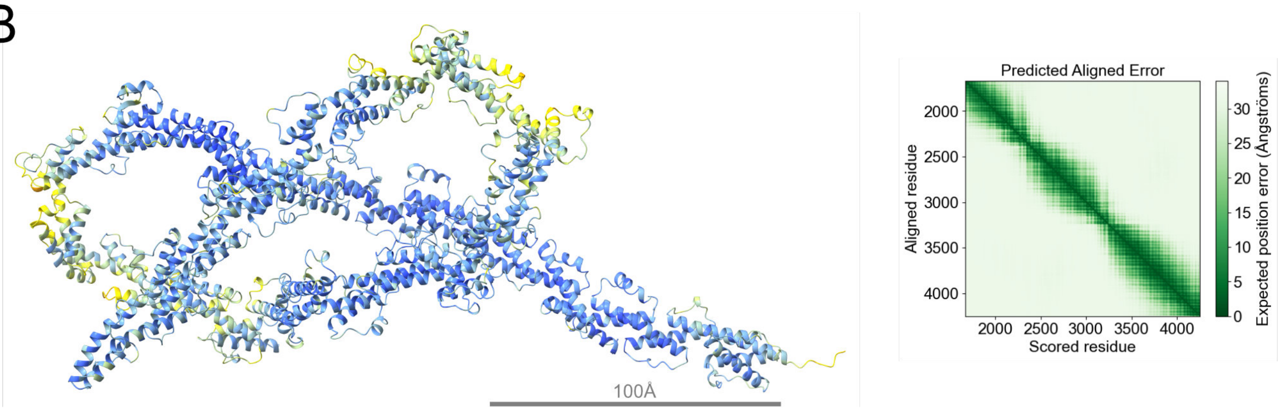
100Å
Predicted Aligned Error
Aligned residue
Scored residue
2000 2500 3000 3500 4000
Expected position error (Ångströms)
0 5 10 15 20 25 30

Figure S3

A

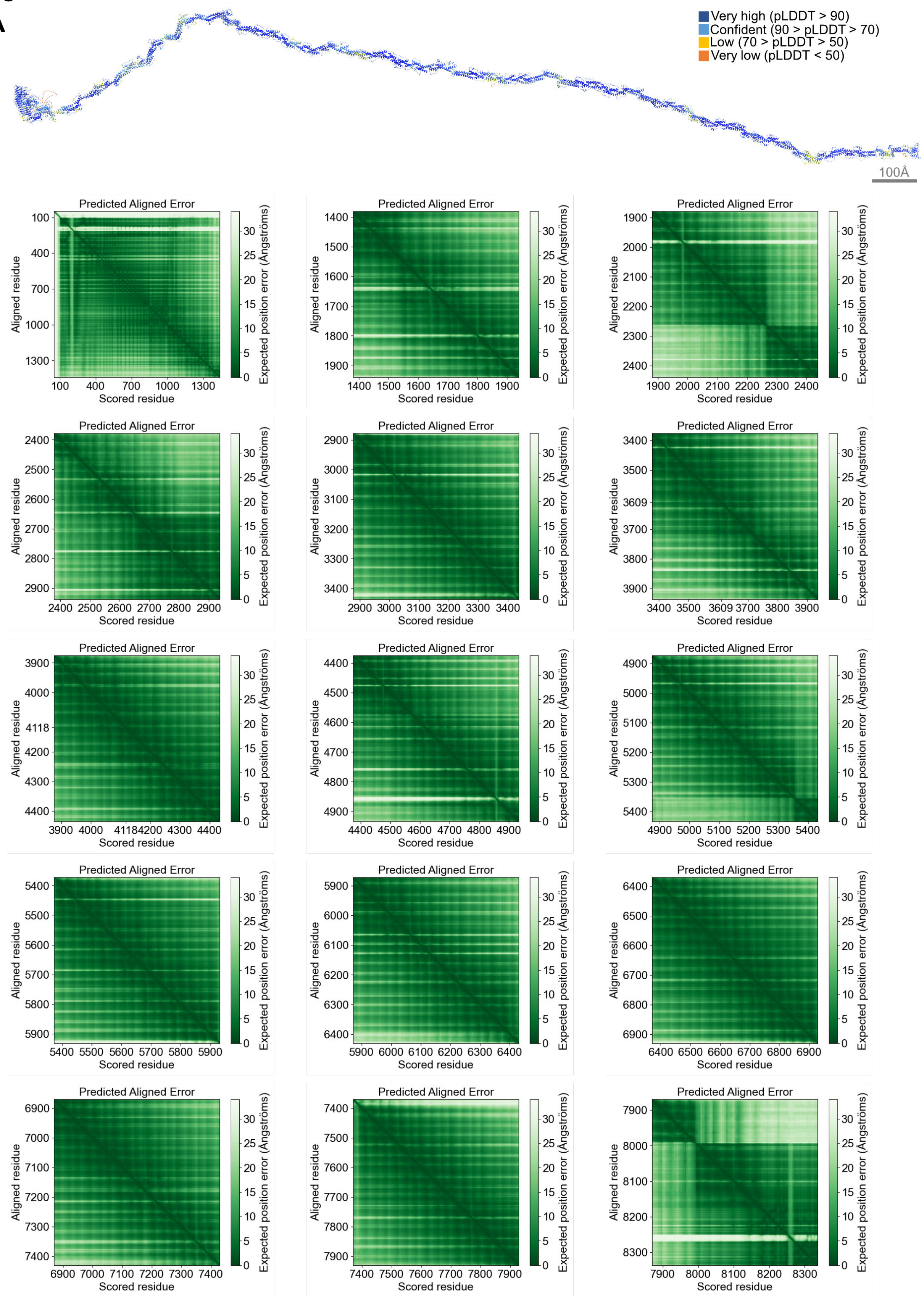
Very high (pLDDT > 90)
Confident (90 > pLDDT > 70)
Low (70 > pLDDT > 50)
Very low (pLDDT < 50)
100Å
Predicted Aligned Error
Aligned residue
Scored residue
Expected position error (Ångströms)
0 5 10 15 20 25 30
100 400 700 1000 1300
1400 1500 1600 1700 1800 1900
1900 2000 2100 2200 2300 2400
2400 2500 2600 2700 2800 2900
2900 3000 3100 3200 3300 3400
3400 3500 3609 3700 3800 3900
3900 4000 4118 4200 4300 4400
4400 4500 4600 4700 4800 4900
4900 5000 5100 5200 5300 5400
5400 5500 5600 5700 5800 5900
5900 6000 6100 6200 6300 6400
6400 6500 6600 6700 6800 6900
6900 7000 7100 7200 7300 7400
7400 7500 7600 7700 7800 7900
7900 8000 8100 8200 8300

Figure S3

B

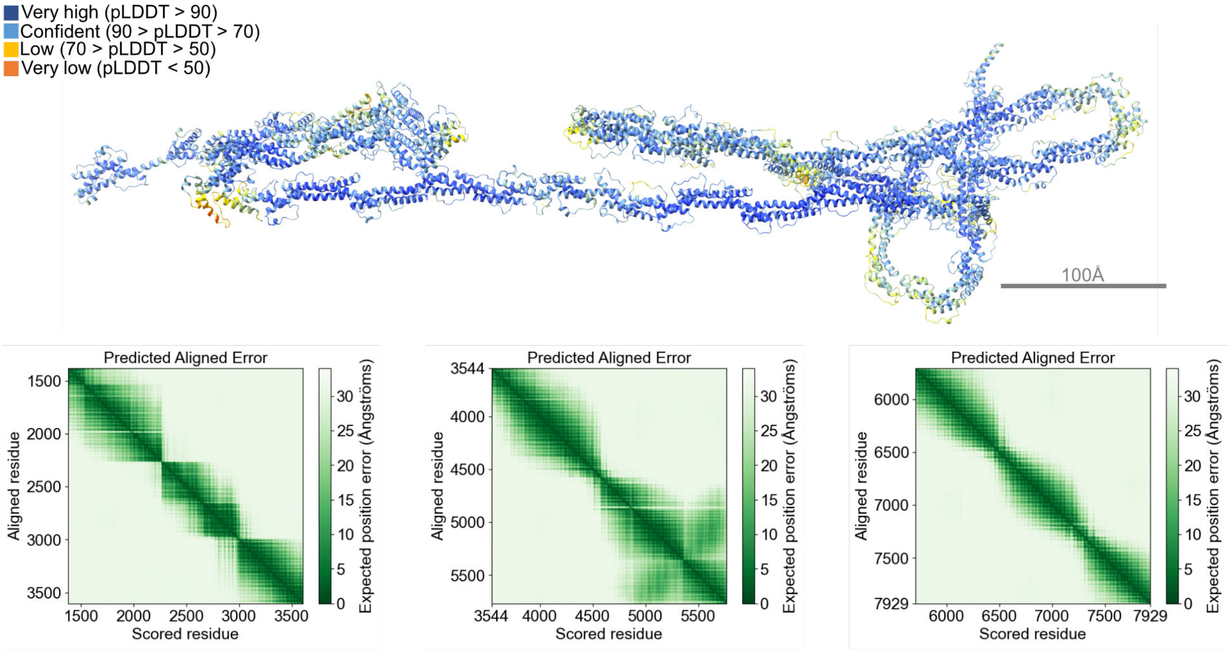

C

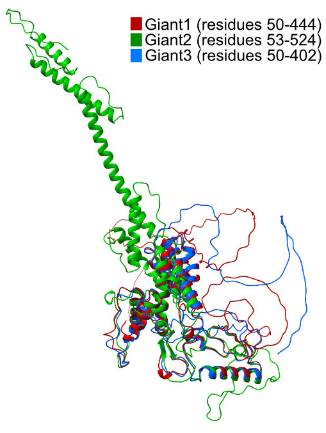

D

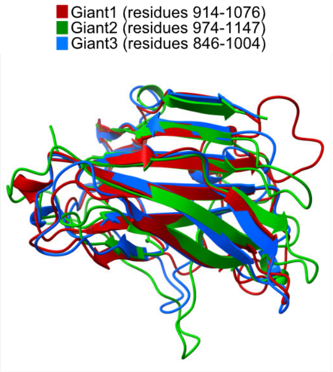

Figure S4

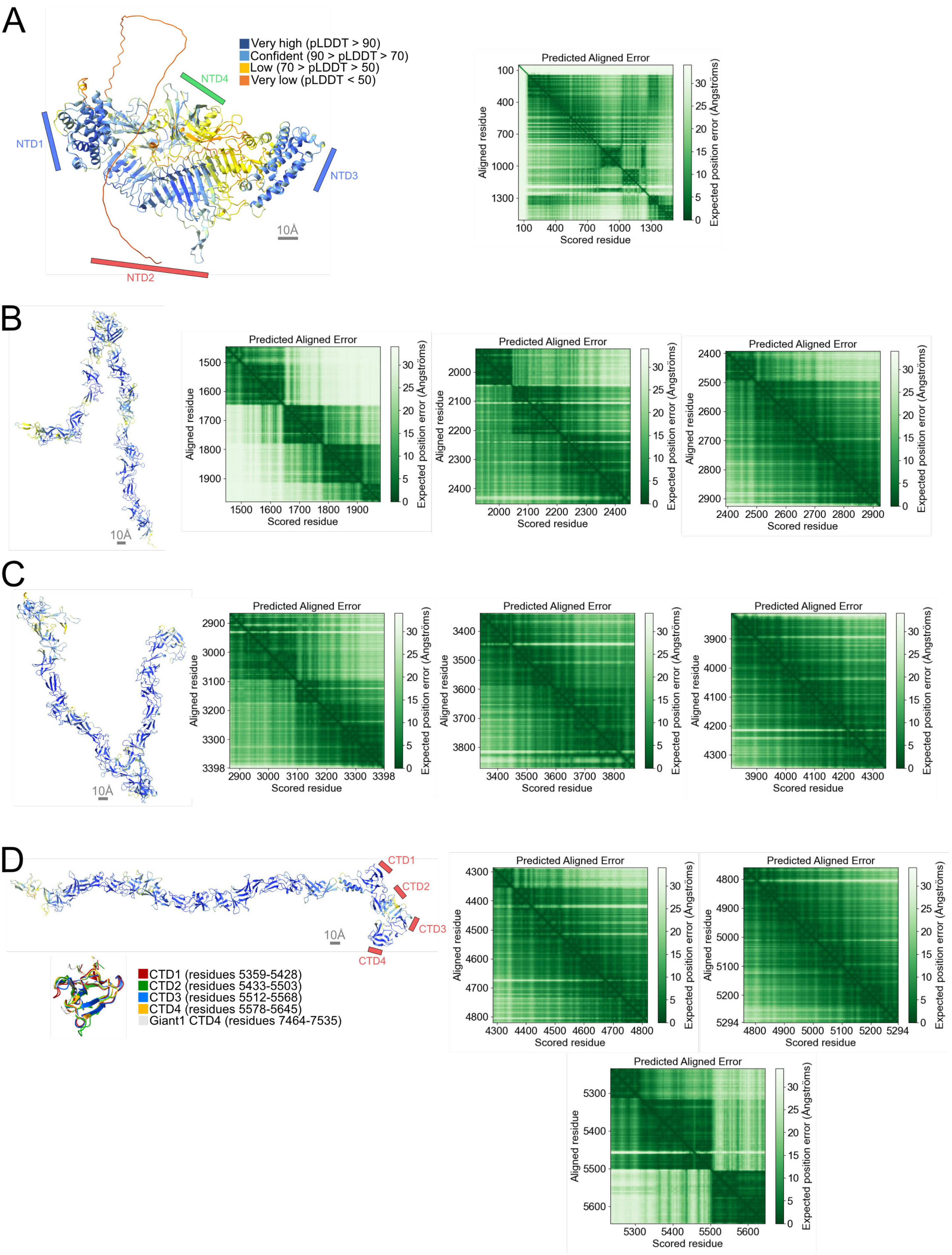
A
Very high (pLDDT > 90)
Confident (90 > pLDDT > 70)
Low (70 > pLDDT > 50)
Very low (pLDDT < 50)
NTD1
NTD2
NTD3
NTD4
10Å
Predicted Aligned Error
Aligned residue
Scored residue
Expected position error (Ångströms)
B
C
D
CTD1
CTD2
CTD3
CTD4
CTD1 (residues 5359-5428)
CTD2 (residues 5433-5503)
CTD3 (residues 5512-5568)
CTD4 (residues 5578-5645)
Giant1 CTD4 (residues 7464-7535)

Figure S4

E

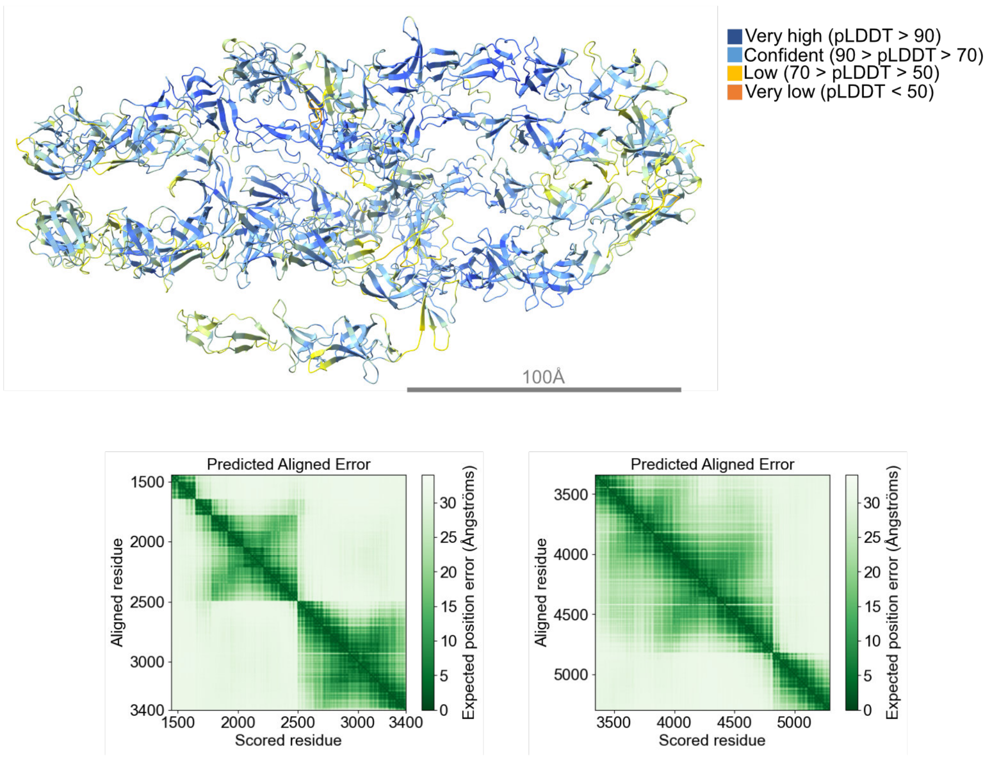
Very high (pLDDT > 90)
Confident (90 > pLDDT > 70)
Low (70 > pLDDT > 50)
Very low (pLDDT < 50)
100Å
Predicted Aligned Error
Aligned residue
Scored residue
Expected position error (Ångströms)
1500
2000
2500
3000
3400
0
5
10
15
20
25
30
Predicted Aligned Error
Aligned residue
Scored residue
Expected position error (Ångströms)
3500
4000
4500
5000

Figure S5

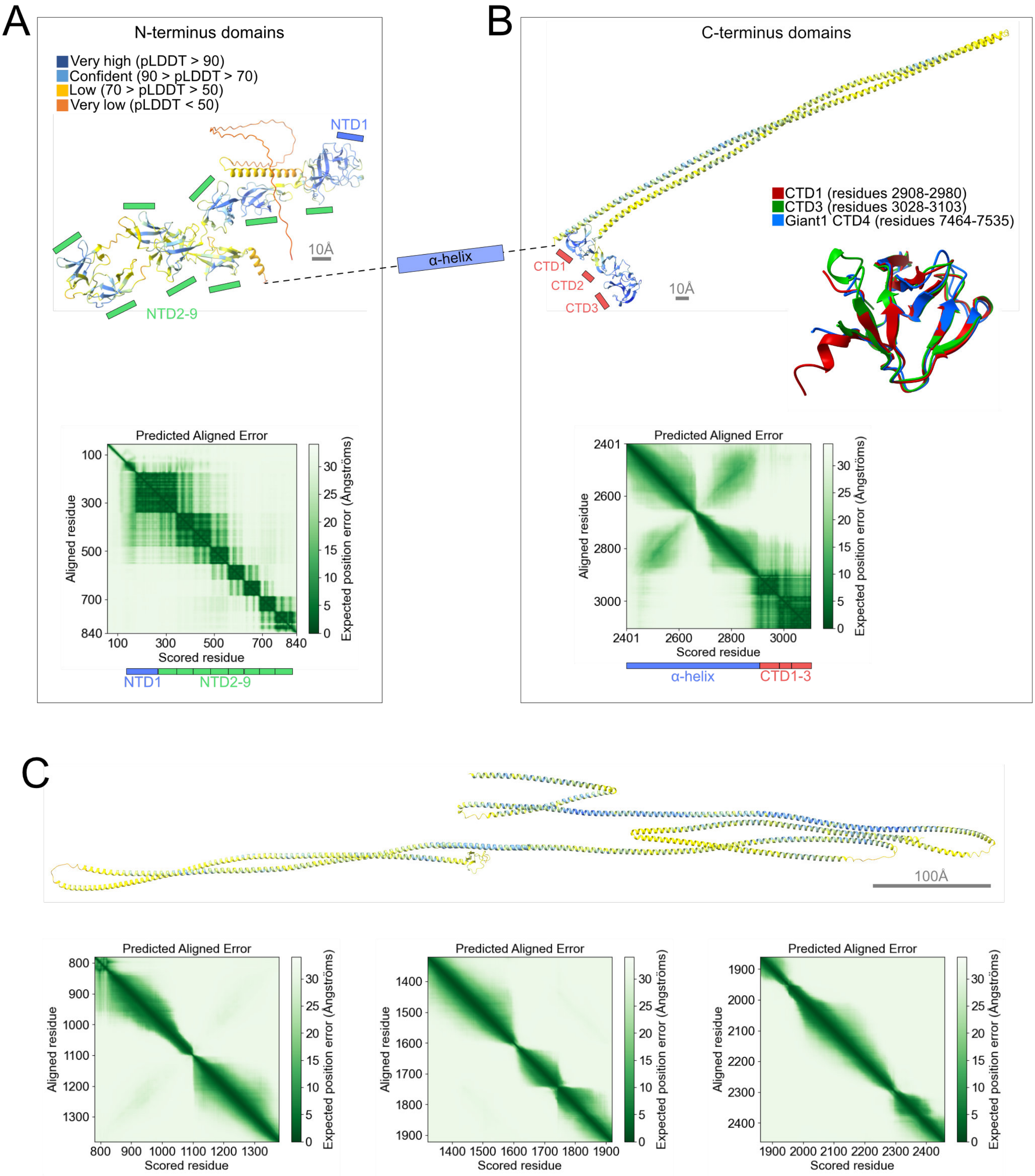

A
N-terminus domains
Very high (pLDDT > 90)
Confident (90 > pLDDT > 70)
Low (70 > pLDDT > 50)
Very low (pLDDT < 50)
NTD1
10Å
NTD2-9
α-helix
Predicted Aligned Error
Aligned residue
Scored residue
Expected position error (Ångströms)
NTD1
NTD2-9
B
C-terminus domains
CTD1 (residues 2908-2980)
CTD3 (residues 3028-3103)
Giant1 CTD4 (residues 7464-7535)
CTD1
CTD2
CTD3
10Å
Predicted Aligned Error
Aligned residue
Scored residue
Expected position error (Ångströms)
α-helix
CTD1-3
C
100Å
Predicted Aligned Error
Aligned residue
Scored residue
Expected position error (Ångströms)

Figure S5

D

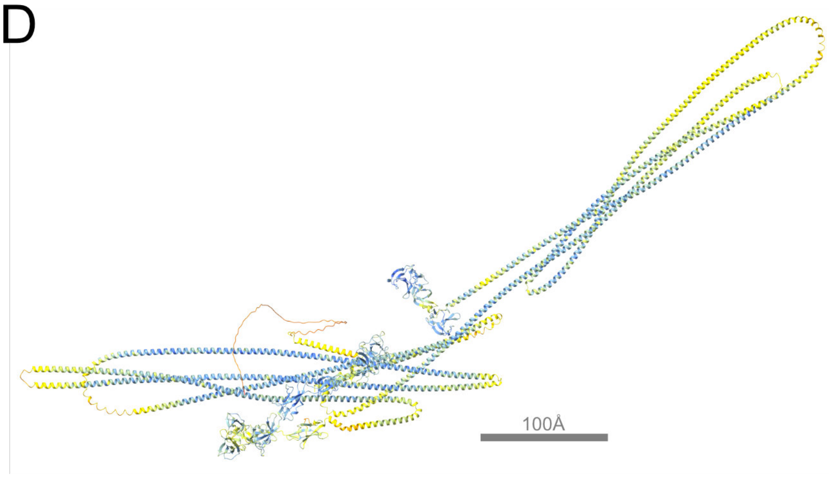
100Å

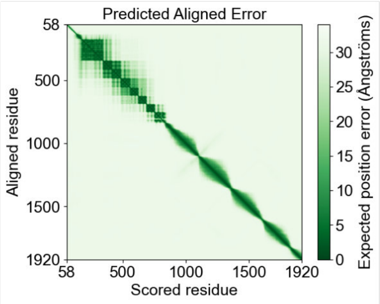
Predicted Aligned Error
Aligned residue
Scored residue
Expected position error (Ångströms)
58
500
1000
1500
1920

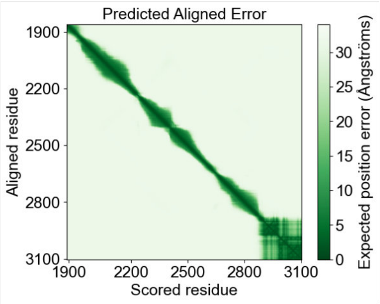
Predicted Aligned Error
Aligned residue
Scored residue
Expected position error (Ångströms)
1900
2200
2500
2800
3100

Figure S6

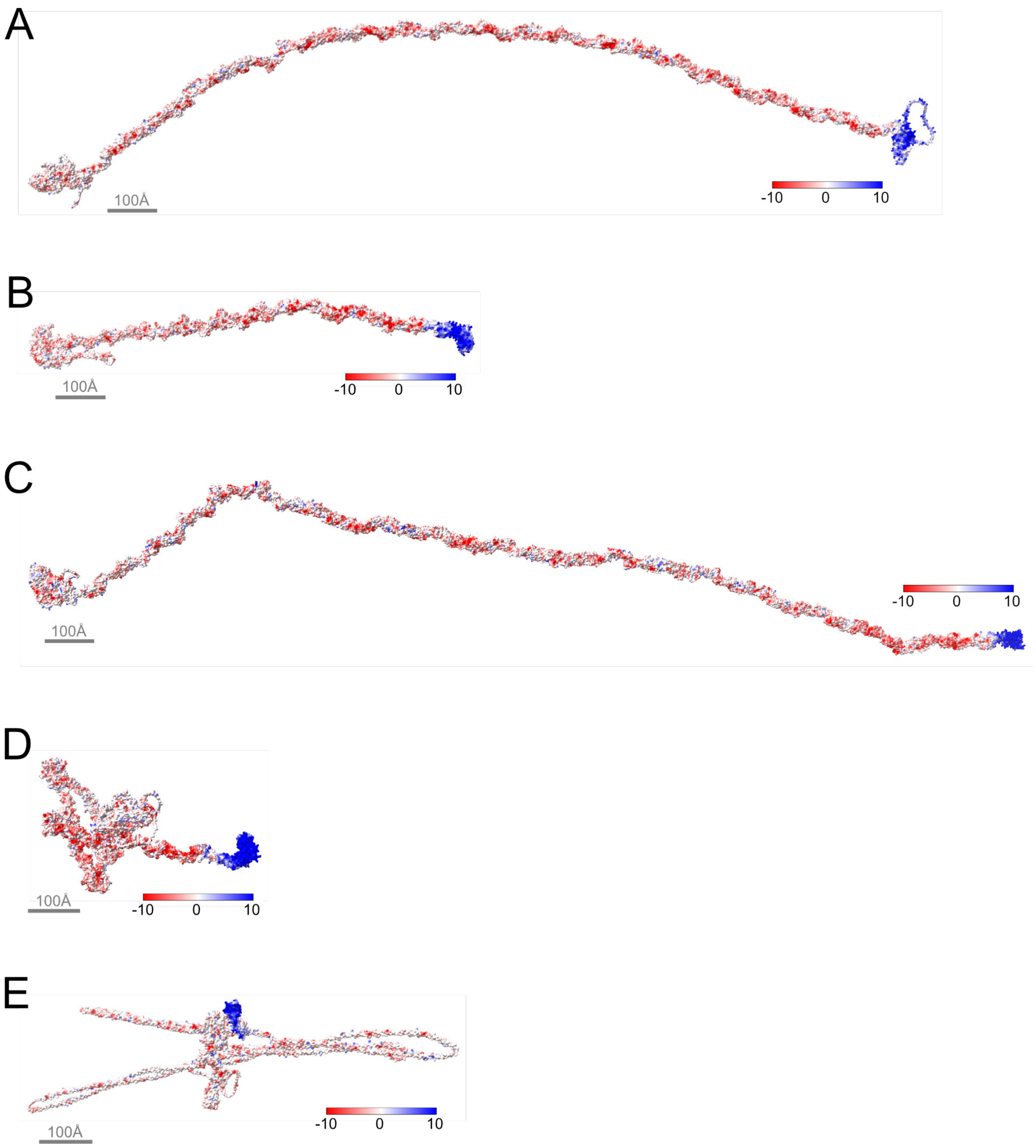
A
100Å
-10
0
10
B
100Å
-10
0
10
C
100Å
-10
0
10
D
100Å
-10
0
10
E
100Å
-10
0
10

Figure S7

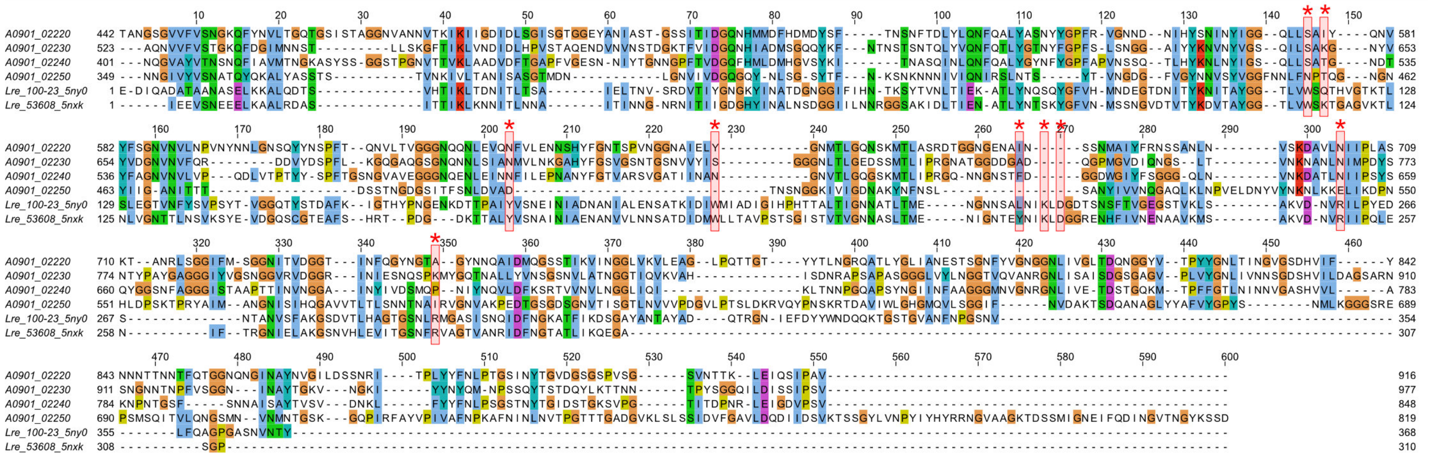
A0901_02220
A0901_02230
A0901_02240
A0901_02250
Lre_100-23_5ny0
Lre_53608_5nxk

Figure S8

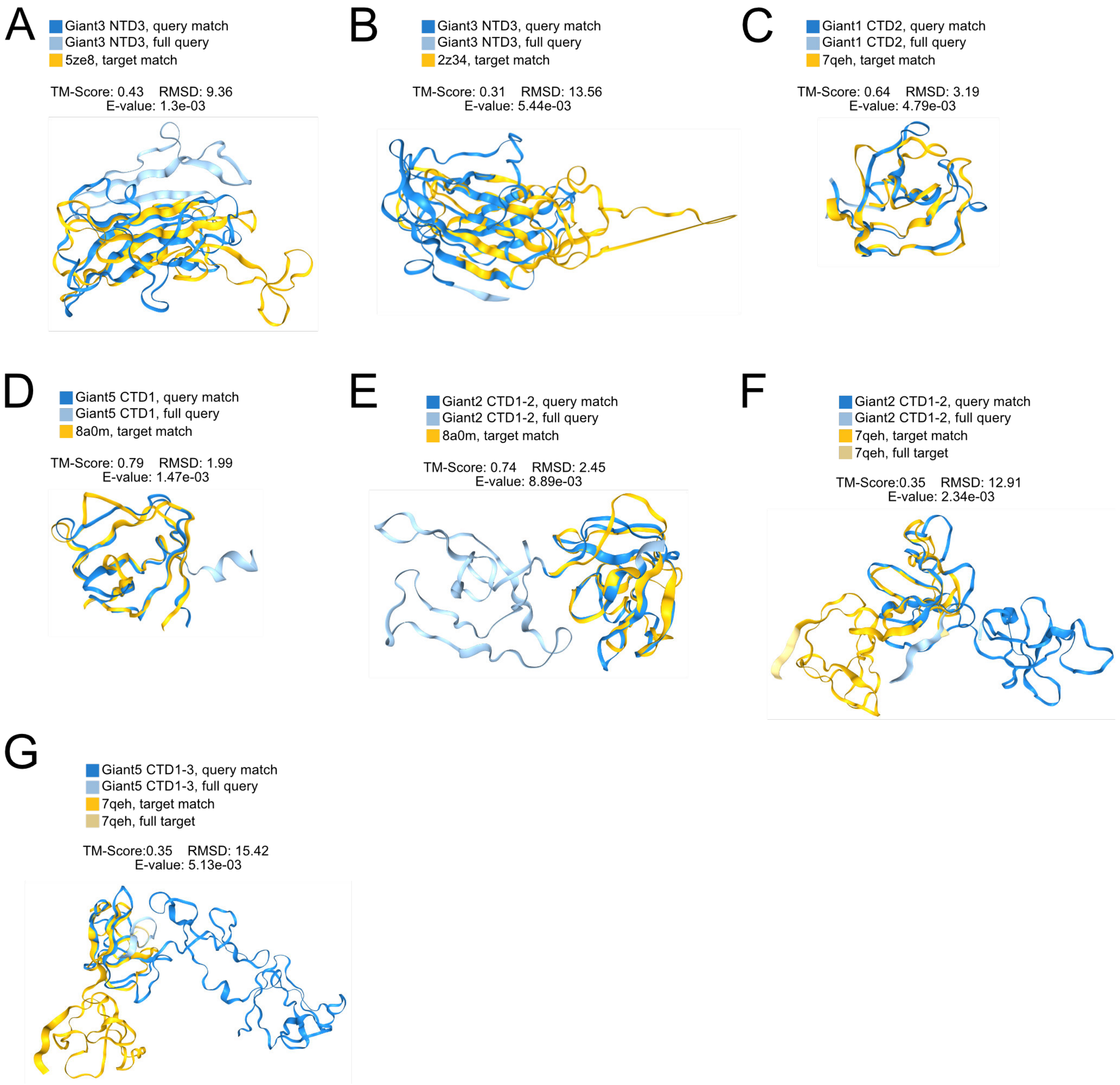
A
Giant3 NTD3, query match
Giant3 NTD3, full query
5ze8, target match
TM-Score: 0.43 RMSD: 9.36
E-value: 1.3e-03
B
Giant3 NTD3, query match
Giant3 NTD3, full query
2z34, target match
TM-Score: 0.31 RMSD: 13.56
E-value: 5.44e-03
C
Giant1 CTD2, query match
Giant1 CTD2, full query
7qeh, target match
TM-Score: 0.64 RMSD: 3.19
E-value: 4.79e-03
D
Giant5 CTD1, query match
Giant5 CTD1, full query
8a0m, target match
TM-Score: 0.79 RMSD: 1.99
E-value: 1.47e-03
E
Giant2 CTD1-2, query match
Giant2 CTD1-2, full query
8a0m, target match
TM-Score: 0.74 RMSD: 2.45
E-value: 8.89e-03
F
Giant2 CTD1-2, query match
Giant2 CTD1-2, full query
7qeh, target match
7qeh, full target
TM-Score:0.35 RMSD: 12.91
E-value: 2.34e-03
G
Giant5 CTD1-3, query match
Giant5 CTD1-3, full query
7qeh, target match
7qeh, full target
TM-Score:0.35 RMSD: 15.42
E-value: 5.13e-03

Figure S9

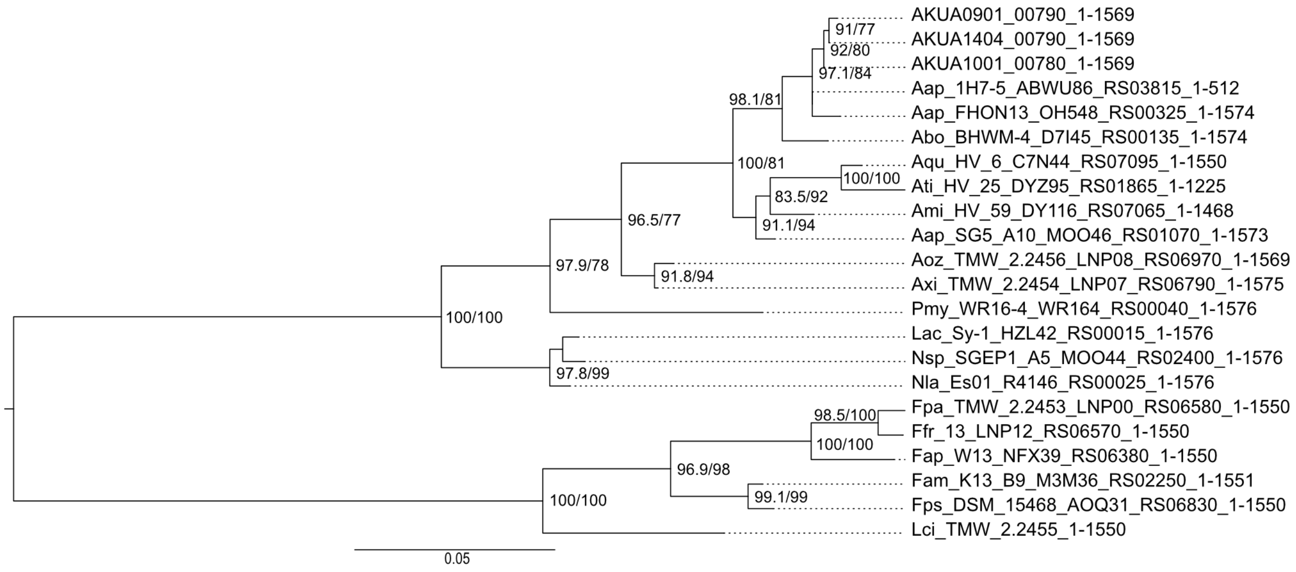

AKUA0901_00790_1-1569
AKUA1404_00790_1-1569
AKUA1001_00780_1-1569
Aap_1H7-5_ABWU86_RS03815_1-512
Aap_FHON13_OH548_RS00325_1-1574
Abo_BHWM-4_D7I45_RS00135_1-1574
Aqu_HV_6_C7N44_RS07095_1-1550
Ati_HV_25_DYZ95_RS01865_1-1225
Ami_HV_59_DY116_RS07065_1-1468
Aap_SG5_A10_MOO46_RS01070_1-1573
Aoz_TMW_2.2456_LNP08_RS06970_1-1569
Axi_TMW_2.2454_LNP07_RS06790_1-1575
Pmy_WR16-4_WR164_RS00040_1-1576
Lac_Sy-1_HZL42_RS00015_1-1576
Nsp_SGEP1_A5_MOO44_RS02400_1-1576
Nla_Es01_R4146_RS00025_1-1576
Fpa_TMW_2.2453_LNP00_RS06580_1-1550
Ffr_13_LNP12_RS06570_1-1550
Fap_W13_NFX39_RS06380_1-1550
Fam_K13_B9_M3M36_RS02250_1-1551
Fps_DSM_15468_AOQ31_RS06830_1-1550
Lci_TMW_2.2455_1-1550
91/77
92/80
97.1/84
98.1/81
100/81
100/100
83.5/92
91.1/94
96.5/77
97.9/78
91.8/94
100/100
97.8/99
98.5/100
100/100
96.9/98
99.1/99
100/100
0.05

Figure S10

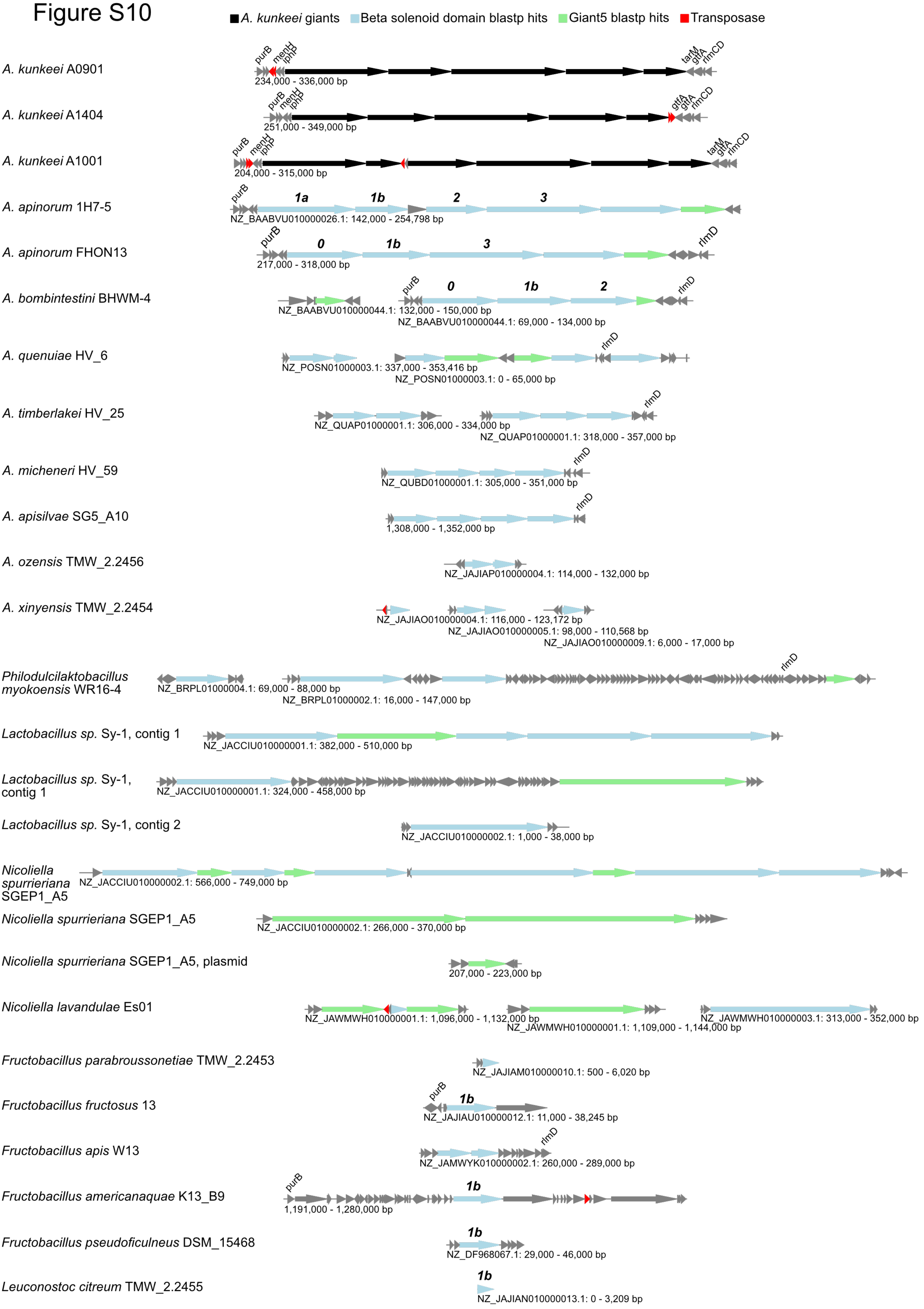
A. kunkeei giants
Beta solenoid domain blastp hits
Giant5 blastp hits
Transposase
A. kunkeei A0901
purB
menH
iphP
tarM
gtfA
rlmCD
234,000 - 336,000 bp
A. kunkeei A1404
251,000 - 349,000 bp
A. kunkeei A1001
204,000 - 315,000 bp
A. apinorum 1H7-5
1a
1b
2
3
NZ_BAABVU010000026.1: 142,000 - 254,798 bp
A. apinorum FHON13
0
rlmD
217,000 - 318,000 bp
A. bombintestini BHWM-4
NZ_BAABVU010000044.1: 132,000 - 150,000 bp
NZ_BAABVU010000044.1: 69,000 - 134,000 bp
A. quenuiae HV_6
NZ_POSN01000003.1: 337,000 - 353,416 bp
NZ_POSN01000003.1: 0 - 65,000 bp
A. timberlakei HV_25
NZ_QUAP01000001.1: 306,000 - 334,000 bp
NZ_QUAP01000001.1: 318,000 - 357,000 bp
A. micheneri HV_59
NZ_QUBD01000001.1: 305,000 - 351,000 bp
A. apisilvae SG5_A10
1,308,000 - 1,352,000 bp
A. ozensis TMW_2.2456
NZ_JAJIAP010000004.1: 114,000 - 132,000 bp
A. xinyensis TMW_2.2454
NZ_JAJIAO010000004.1: 116,000 - 123,172 bp
NZ_JAJIAO010000005.1: 98,000 - 110,568 bp
NZ_JAJIAO010000009.1: 6,000 - 17,000 bp
Philodulcilaktobacillus myokoensis WR16-4
NZ_BRPL01000004.1: 69,000 - 88,000 bp
NZ_BRPL01000002.1: 16,000 - 147,000 bp
Lactobacillus sp. Sy-1, contig 1
NZ_JACCIU010000001.1: 382,000 - 510,000 bp
Lactobacillus sp. Sy-1, contig 1
NZ_JACCIU010000001.1: 324,000 - 458,000 bp
Lactobacillus sp. Sy-1, contig 2
NZ_JACCIU010000002.1: 1,000 - 38,000 bp
Nicoliella spurrieriana SGEP1_A5
NZ_JACCIU010000002.1: 566,000 - 749,000 bp
Nicoliella spurrieriana SGEP1_A5
NZ_JACCIU010000002.1: 266,000 - 370,000 bp
Nicoliella spurrieriana SGEP1_A5, plasmid
207,000 - 223,000 bp
Nicoliella lavandulae Es01
NZ_JAWMWH010000001.1: 1,096,000 - 1,132,000 bp
NZ_JAWMWH010000001.1: 1,109,000 - 1,144,000 bp
NZ_JAWMWH010000003.1: 313,000 - 352,000 bp
Fructobacillus parabroussonetiae TMW_2.2453
NZ_JAJIAM010000010.1: 500 - 6,020 bp
Fructobacillus fructosus 13
NZ_JAJIAU010000012.1: 11,000 - 38,245 bp
Fructobacillus apis W13
NZ_JAMWYK010000002.1: 260,000 - 289,000 bp
Fructobacillus americanaquae K13_B9
1,191,000 - 1,280,000 bp
Fructobacillus pseudoficulneus DSM_15468
NZ_DF968067.1: 29,000 - 46,000 bp
Leuconostoc citreum TMW_2.2455
NZ_JAJIAN010000013.1: 0 - 3,209 bp

Figure S11

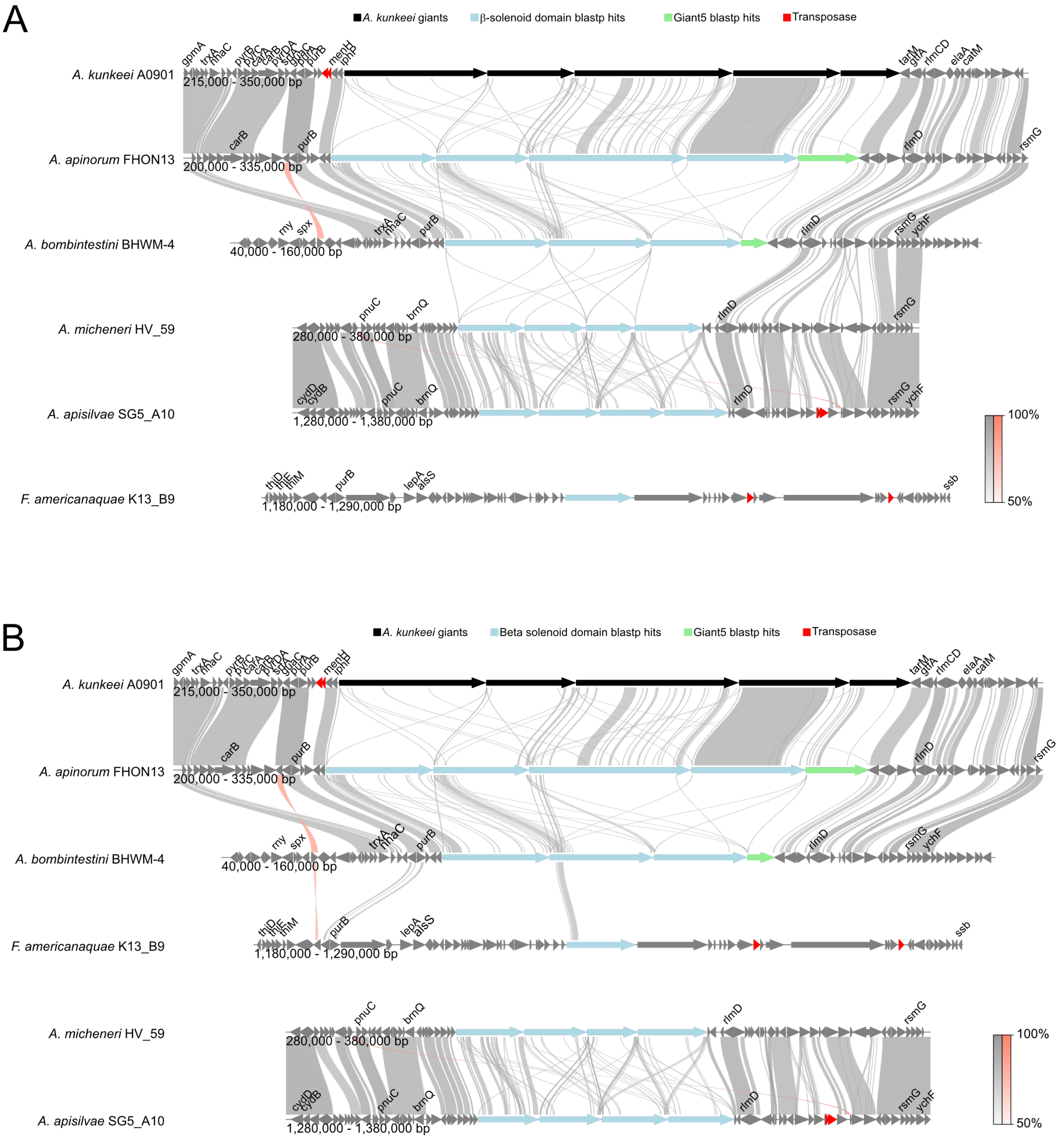
A
A. kunkeei giants
β-solenoid domain blastp hits
Giant5 blastp hits
Transposase
A. kunkeei A0901
215,000 - 350,000 bp
A. apinorum FHON13
200,000 - 335,000 bp
A. bombintestini BHWM-4
40,000 - 160,000 bp
A. micheneri HV_59
280,000 - 380,000 bp
A. apisilvae SG5_A10
1,280,000 - 1,380,000 bp
F. americanaquae K13_B9
1,180,000 - 1,290,000 bp
100%
50%
B
A. kunkeei giants
Beta solenoid domain blastp hits
Giant5 blastp hits
Transposase
A. kunkeei A0901
215,000 - 350,000 bp
A. apinorum FHON13
200,000 - 335,000 bp
A. bombintestini BHWM-4
40,000 - 160,000 bp
F. americanaquae K13_B9
1,180,000 - 1,290,000 bp
A. micheneri HV_59
280,000 - 380,000 bp
A. apisilvae SG5_A10
1,280,000 - 1,380,000 bp
100%
50%

Figure S11C

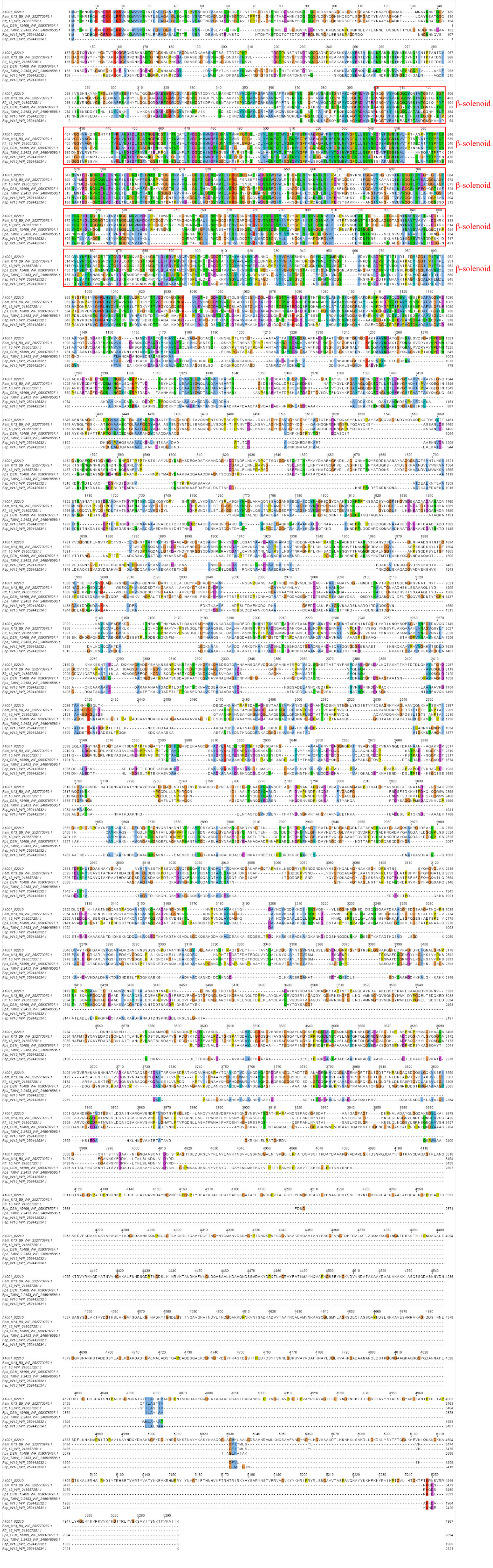

Figure S12

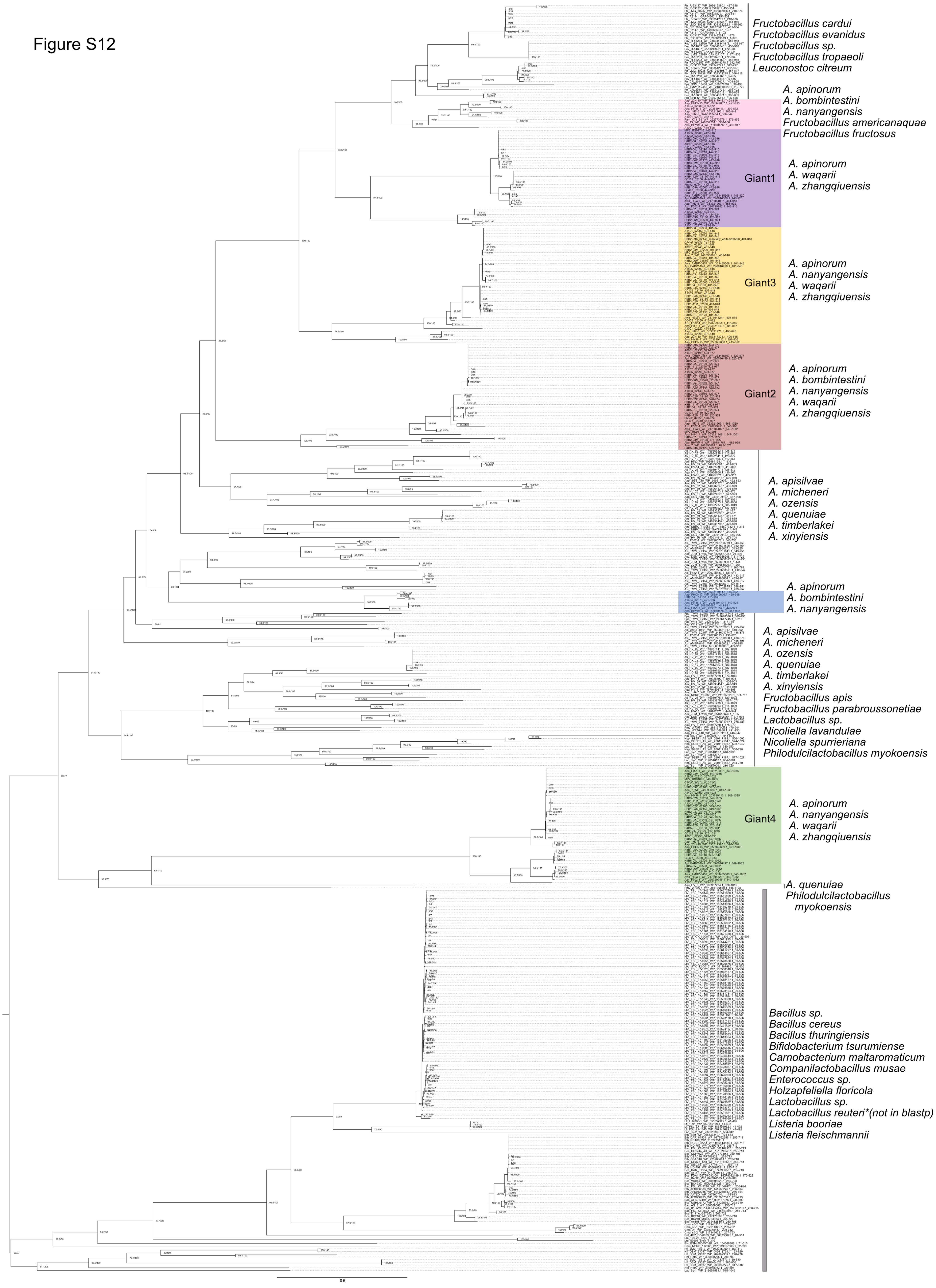

Fructobacillus cardui
Fructobacillus evanidus
Fructobacillus sp.
Fructobacillus tropaeoli
Leuconostoc citreum
A. apinorum
A. bombintestini
A. nanyangensis
Fructobacillus americanaquae
Fructobacillus fructosus
Giant1
A. apinorum
A. waqarii
A. zhangqiuensis
Giant3
A. apinorum
A. nanyangensis
A. waqarii
A. zhangqiuensis
Giant2
A. apinorum
A. bombintestini
A. nanyangensis
A. waqarii
A. zhangqiuensis
A. apisilvae
A. micheneri
A. ozensis
A. quenuiae
A. timberlakei
A. xinyiensis
A. apinorum
A. bombintestini
A. nanyangensis
A. apisilvae
A. micheneri
A. ozensis
A. quenuiae
A. timberlakei
A. xinyiensis
Fructobacillus apis
Fructobacillus parabroussonetiae
Lactobacillus sp.
Nicoliella lavandulae
Nicoliella spurrieriana
Philodulcilactobacillus myokoensis
Giant4
A. apinorum
A. nanyangensis
A. waqarii
A. zhangqiuensis
A. quenuiae
Philodulcilactobacillus myokoensis
Bacillus sp.
Bacillus cereus
Bacillus thuringiensis
Bifidobacterium tsurumiense
Carnobacterium maltaromaticum
Companilactobacillus musae
Enterococcus sp.
Holzapfeliella floricola
Lactobacillus sp.
Lactobacillus reuteri*(not in blastp)
Listeria booriae
Listeria fleischmannii
0.6

Figure S13

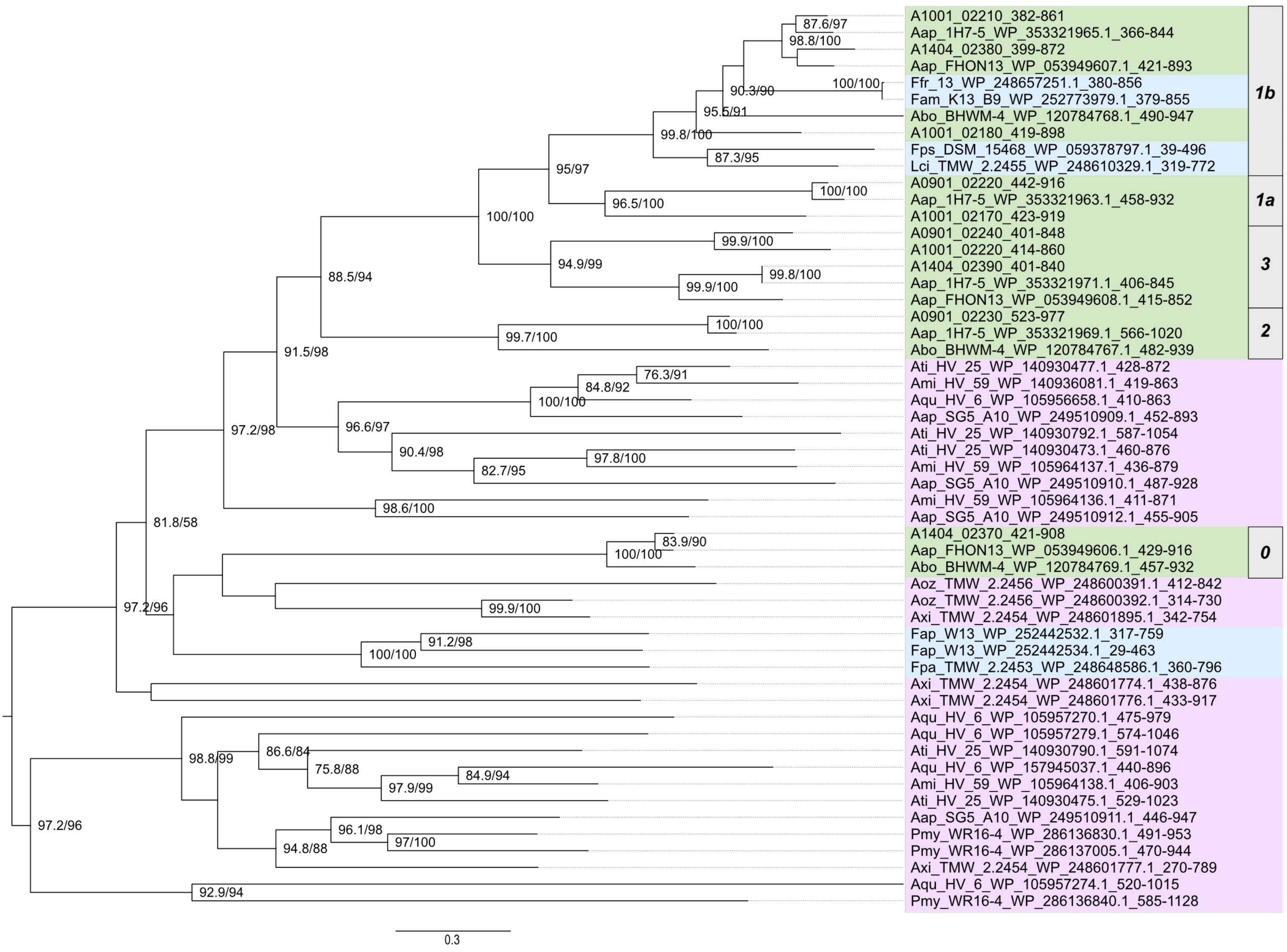

A1001_02210_382-861
Aap_1H7-5_WP_353321965.1_366-844
A1404_02380_399-872
Aap_FHON13_WP_053949607.1_421-893
Ffr_13_WP_248657251.1_380-856
Fam_K13_B9_WP_252773979.1_379-855
Abo_BHWM-4_WP_120784768.1_490-947
A1001_02180_419-898
Fps_DSM_15468_WP_059378797.1_39-496
Lci_TMW_2.2455_WP_248610329.1_319-772
A0901_02220_442-916
Aap_1H7-5_WP_353321963.1_458-932
A1001_02170_423-919
A0901_02240_401-848
A1001_02220_414-860
A1404_02390_401-840
Aap_1H7-5_WP_353321971.1_406-845
Aap_FHON13_WP_053949608.1_415-852
A0901_02230_523-977
Aap_1H7-5_WP_353321969.1_566-1020
Abo_BHWM-4_WP_120784767.1_482-939
Ati_HV_25_WP_140930477.1_428-872
Ami_HV_59_WP_140936081.1_419-863
Aqu_HV_6_WP_105956658.1_410-863
Aap_SG5_A10_WP_249510909.1_452-893
Ati_HV_25_WP_140930792.1_587-1054
Ati_HV_25_WP_140930473.1_460-876
Ami_HV_59_WP_105964137.1_436-879
Aap_SG5_A10_WP_249510910.1_487-928
Ami_HV_59_WP_105964136.1_411-871
Aap_SG5_A10_WP_249510912.1_455-905
A1404_02370_421-908
Aap_FHON13_WP_053949606.1_429-916
Abo_BHWM-4_WP_120784769.1_457-932
Aoz_TMW_2.2456_WP_248600391.1_412-842
Aoz_TMW_2.2456_WP_248600392.1_314-730
Axi_TMW_2.2454_WP_248601895.1_342-754
Fap_W13_WP_252442532.1_317-759
Fap_W13_WP_252442534.1_29-463
Fpa_TMW_2.2453_WP_248648586.1_360-796
Axi_TMW_2.2454_WP_248601774.1_438-876
Axi_TMW_2.2454_WP_248601776.1_433-917
Aqu_HV_6_WP_105957270.1_475-979
Aqu_HV_6_WP_105957279.1_574-1046
Ati_HV_25_WP_140930790.1_591-1074
Aqu_HV_6_WP_157945037.1_440-896
Ami_HV_59_WP_105964138.1_406-903
Ati_HV_25_WP_140930475.1_529-1023
Aap_SG5_A10_WP_249510911.1_446-947
Pmy_WR16-4_WP_286136830.1_491-953
Pmy_WR16-4_WP_286137005.1_470-944
Axi_TMW_2.2454_WP_248601777.1_270-789
Aqu_HV_6_WP_105957274.1_520-1015
Pmy_WR16-4_WP_286136840.1_585-1128
1b
1a
3
2
0
87.6/97
98.8/100
100/100
90.3/90
95.5/91
99.8/100
87.3/95
95/97
100/100
96.5/100
100/100
99.9/100
94.9/99
99.8/100
99.9/100
88.5/94
100/100
99.7/100
91.5/98
76.3/91
84.8/92
100/100
97.2/98
96.6/97
90.4/98
97.8/100
82.7/95
98.6/100
81.8/58
83.9/90
100/100
97.2/96
99.9/100
91.2/98
100/100
98.8/99
86.6/84
75.8/88
84.9/94
97.9/99
96.1/98
97/100
94.8/88
97.2/96
92.9/94
0.3

Figure S14

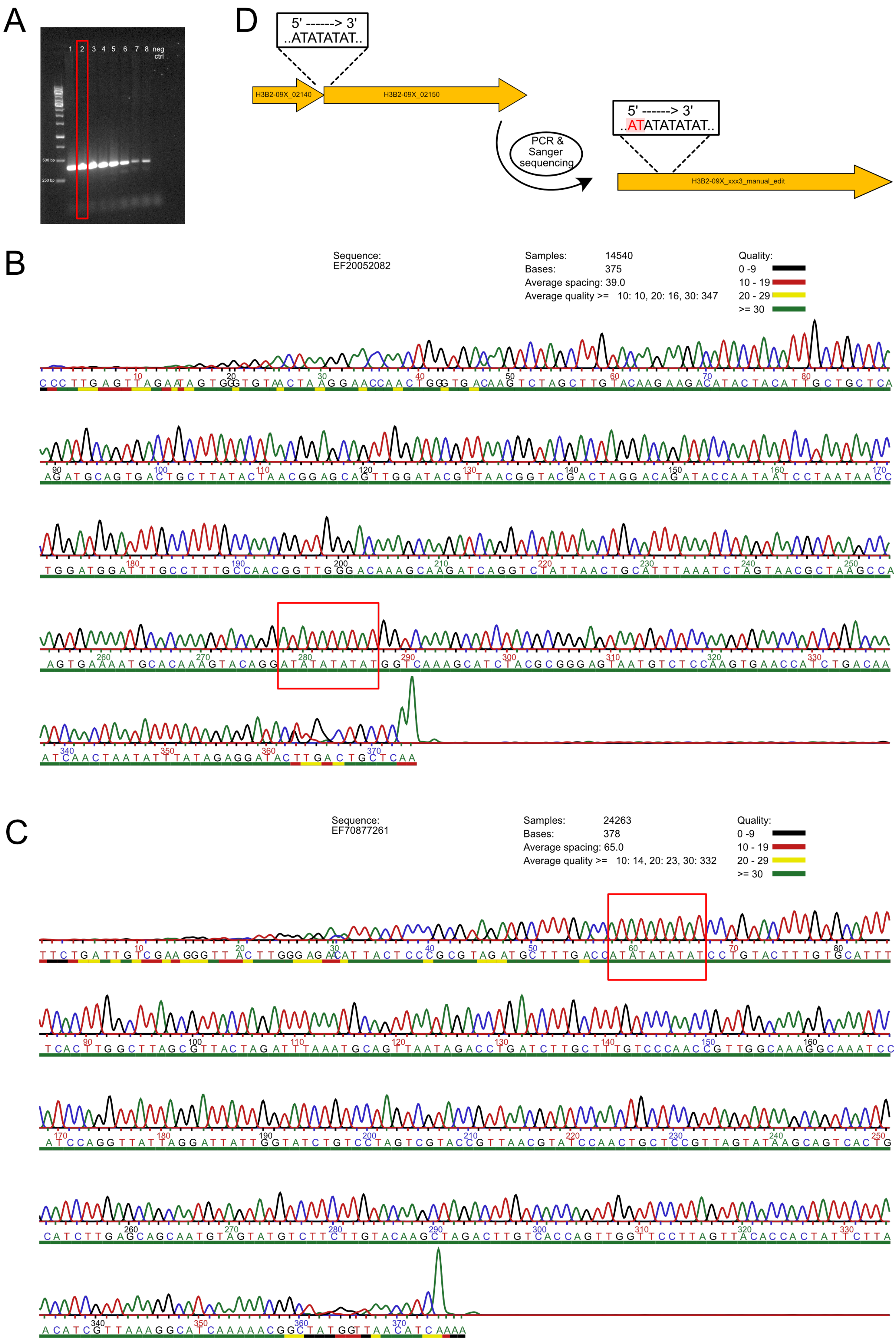
A
1 2 3 4 5 6 7 8 neg ctrl
500 bp
250 bp
D
5' ------> 3'
..ATATATAT..
H3B2-09X_02140
H3B2-09X_02150
PCR &
Sanger
sequencing
5' ------> 3'
..ATATATATAT..
H3B2-09X_xxx3_manual_edit
B
Sequence:
EF20052082
Samples: 14540
Bases: 375
Average spacing: 39.0
Average quality >= 10: 10, 20: 16, 30: 347
Quality:
0 -9
10 - 19
20 - 29
>= 30
C
Sequence:
EF70877261
Samples: 24263
Bases: 378
Average spacing: 65.0
Average quality >= 10: 14, 20: 23, 30: 332
Quality:
0 -9
10 - 19
20 - 29
>= 30

Figure S15

A

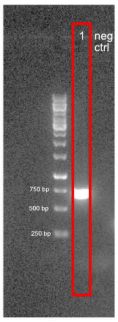

B

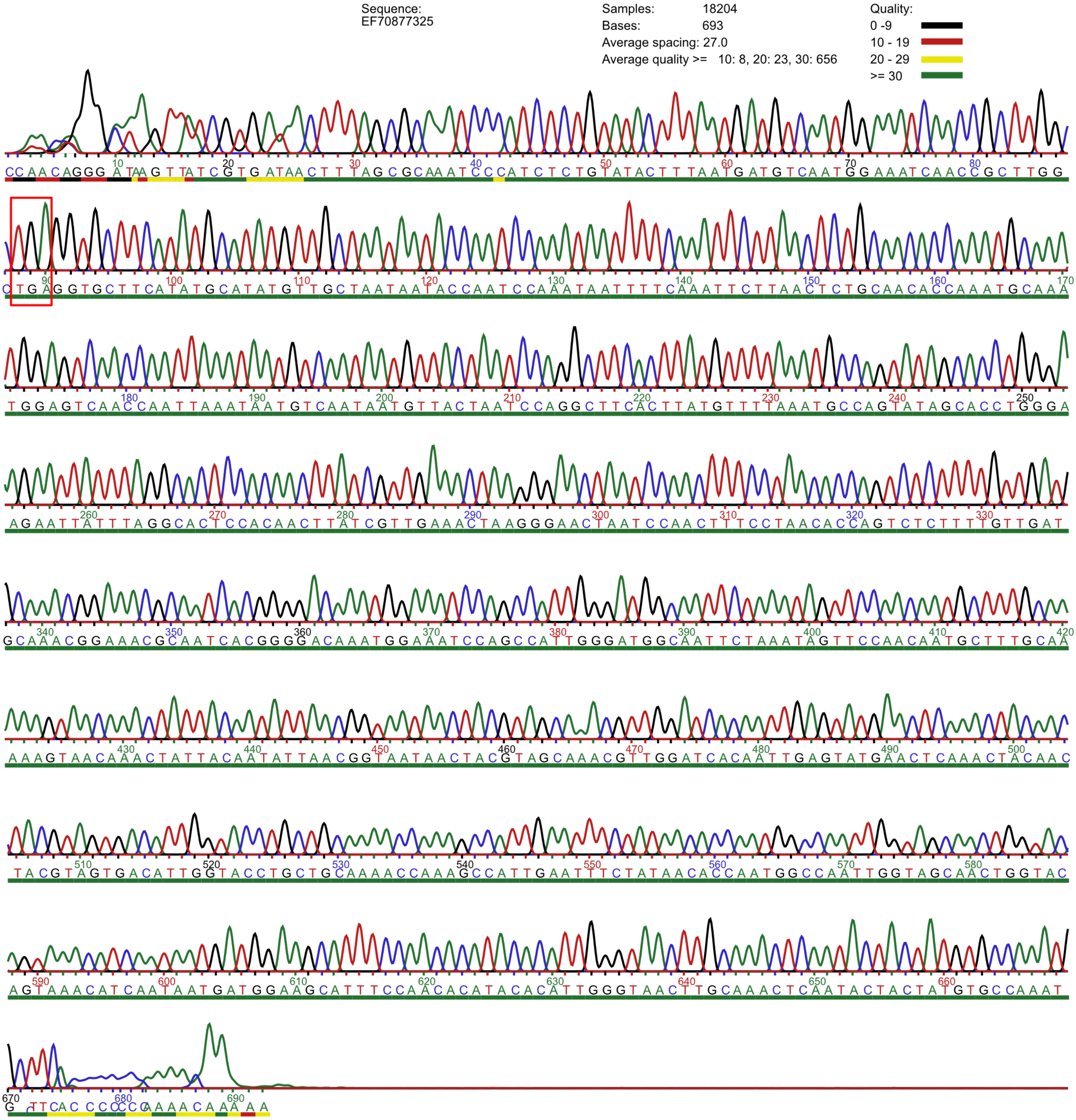

Figure S15

C

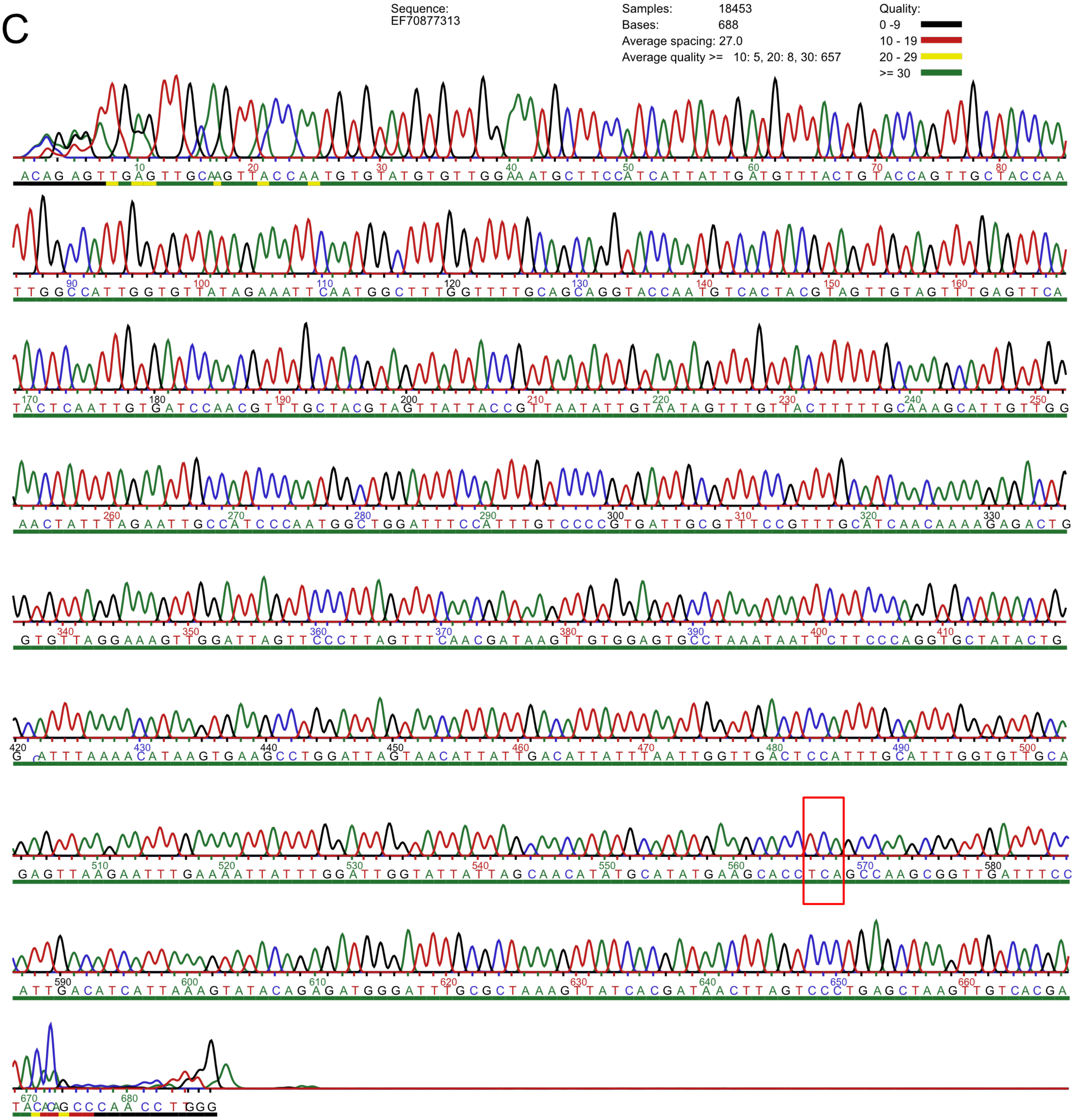

D

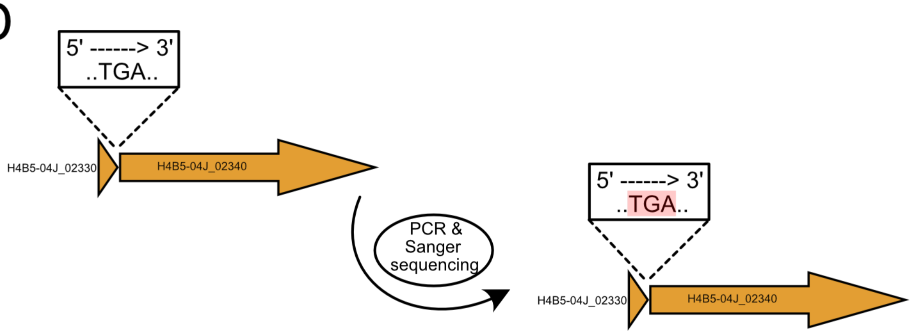

Figure S16

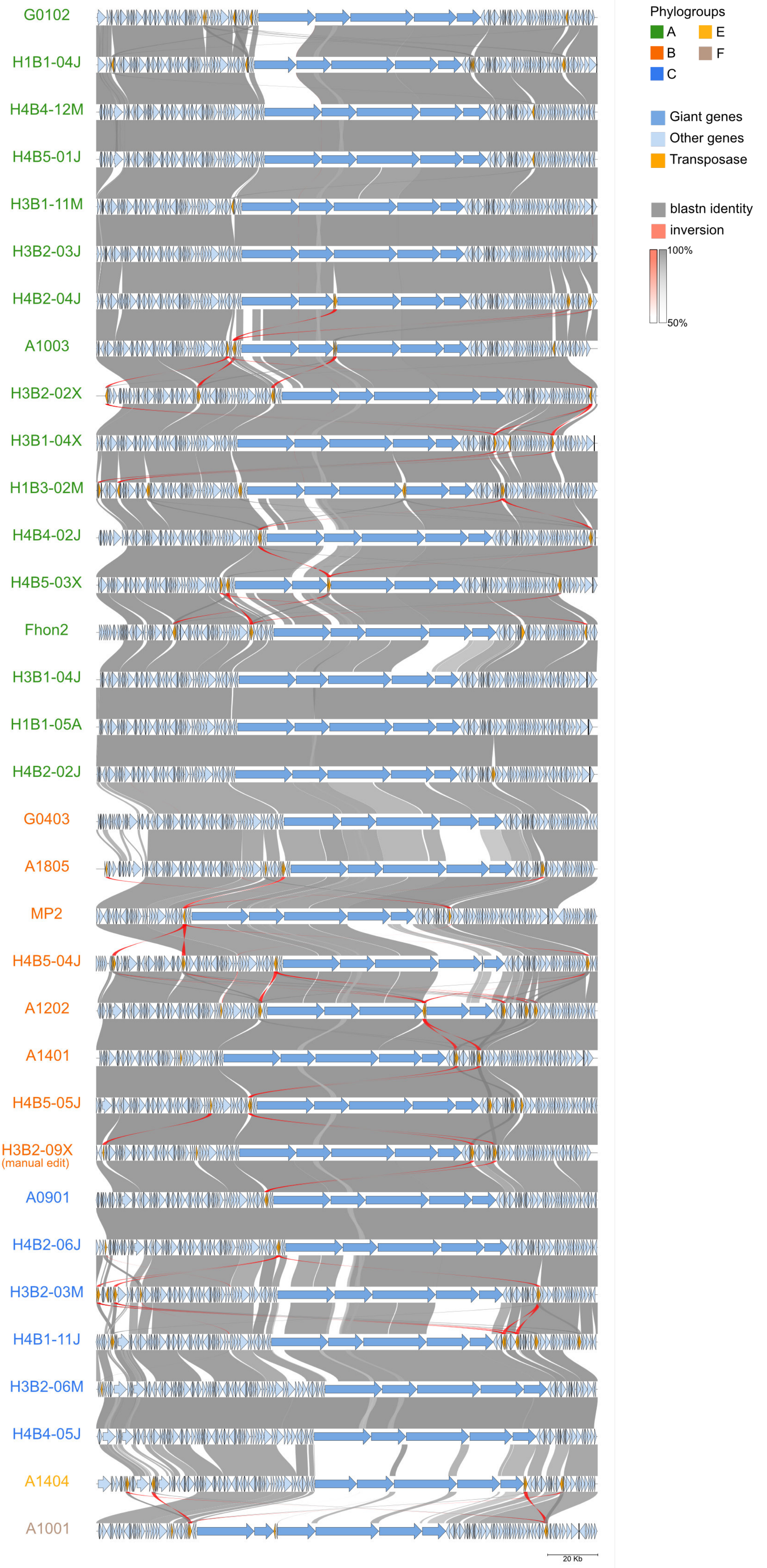
G0102
H1B1-04J
H4B4-12M
H4B5-01J
H3B1-11M
H3B2-03J
H4B2-04J
A1003
H3B2-02X
H3B1-04X
H1B3-02M
H4B4-02J
H4B5-03X
Fhon2
H3B1-04J
H1B1-05A
H4B2-02J
G0403
A1805
MP2
H4B5-04J
A1202
A1401
H4B5-05J
H3B2-09X
(manual edit)
A0901
H4B2-06J
H3B2-03M
H4B1-11J
H3B2-06M
H4B4-05J
A1404
A1001
20 Kb
Phylogroups
A
B
C
E
F
Giant genes
Other genes
Transposase
blastn identity
inversion
100%
50%

Figure S17

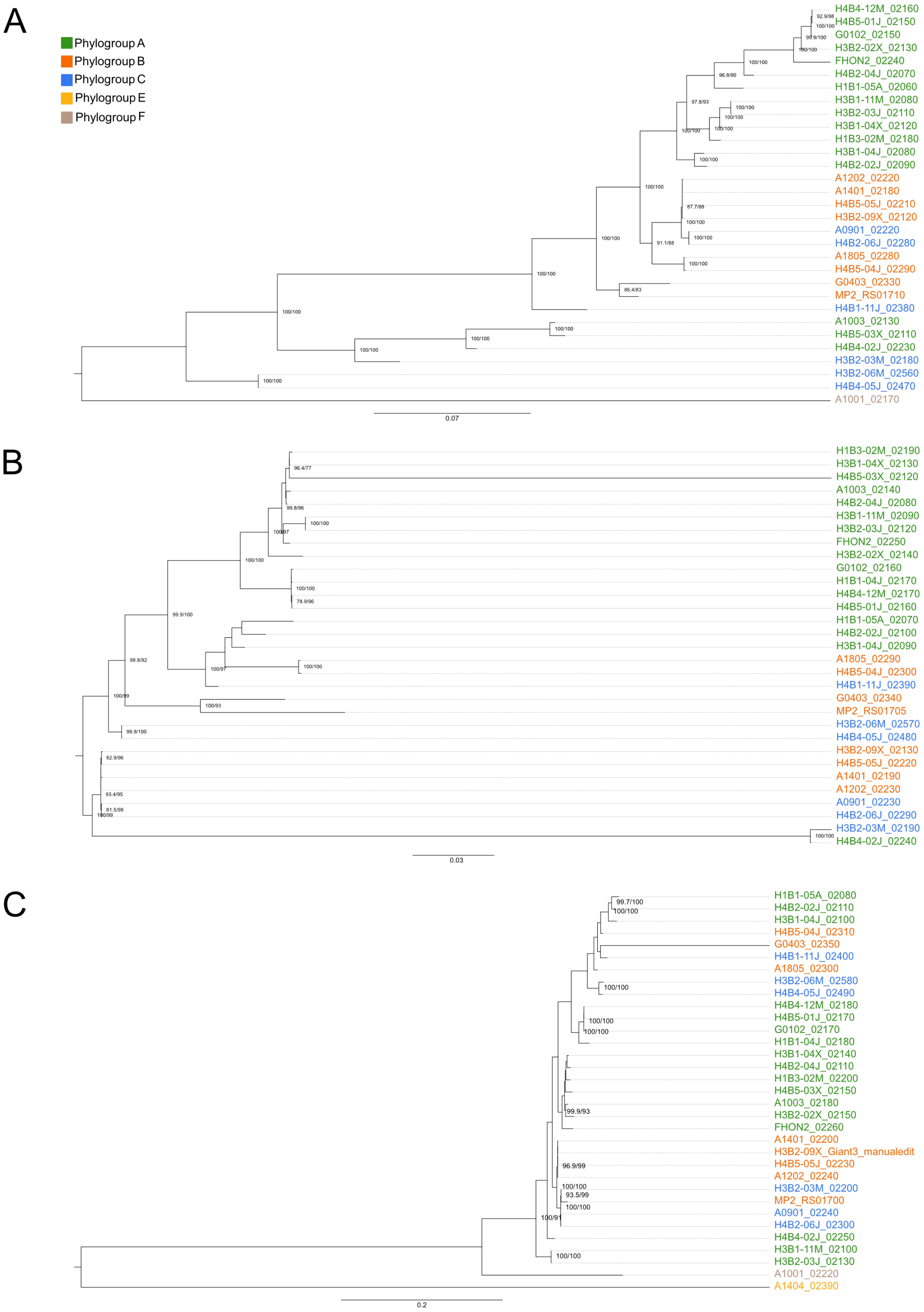

A
Phylogroup A
Phylogroup B
Phylogroup C
Phylogroup E
Phylogroup F
H4B4-12M_02160
H4B5-01J_02150
G0102_02150
H3B2-02X_02130
FHON2_02240
H4B2-04J_02070
H1B1-05A_02060
H3B1-11M_02080
H3B2-03J_02110
H3B1-04X_02120
H1B3-02M_02180
H3B1-04J_02080
H4B2-02J_02090
A1202_02220
A1401_02180
H4B5-05J_02210
H3B2-09X_02120
A0901_02220
H4B2-06J_02280
A1805_02280
H4B5-04J_02290
G0403_02330
MP2_RS01710
H4B1-11J_02380
A1003_02130
H4B5-03X_02110
H4B4-02J_02230
H3B2-03M_02180
H3B2-06M_02560
H4B4-05J_02470
A1001_02170
0.07
B
H1B3-02M_02190
H3B1-04X_02130
H4B5-03X_02120
A1003_02140
H4B2-04J_02080
H3B1-11M_02090
H3B2-03J_02120
FHON2_02250
H3B2-02X_02140
G0102_02160
H1B1-04J_02170
H4B4-12M_02170
H4B5-01J_02160
H1B1-05A_02070
H4B2-02J_02100
H3B1-04J_02090
A1805_02290
H4B5-04J_02300
H4B1-11J_02390
G0403_02340
MP2_RS01705
H3B2-06M_02570
H4B4-05J_02480
H3B2-09X_02130
H4B5-05J_02220
A1401_02190
A1202_02230
A0901_02230
H4B2-06J_02290
H3B2-03M_02190
H4B4-02J_02240
0.03
C
H1B1-05A_02080
H4B2-02J_02110
H3B1-04J_02100
H4B5-04J_02310
G0403_02350
H4B1-11J_02400
A1805_02300
H3B2-06M_02580
H4B4-05J_02490
H4B4-12M_02180
H4B5-01J_02170
G0102_02170
H1B1-04J_02180
H3B1-04X_02140
H4B2-04J_02110
H1B3-02M_02200
H4B5-03X_02150
A1003_02180
H3B2-02X_02150
FHON2_02260
A1401_02200
H3B2-09X_Giant3_manualedit
H4B5-05J_02230
A1202_02240
H3B2-03M_02200
MP2_RS01700
A0901_02240
H4B2-06J_02300
H4B4-02J_02250
H3B1-11M_02100
H3B2-03J_02130
A1001_02220
A1404_02390
0.2

Figure S17

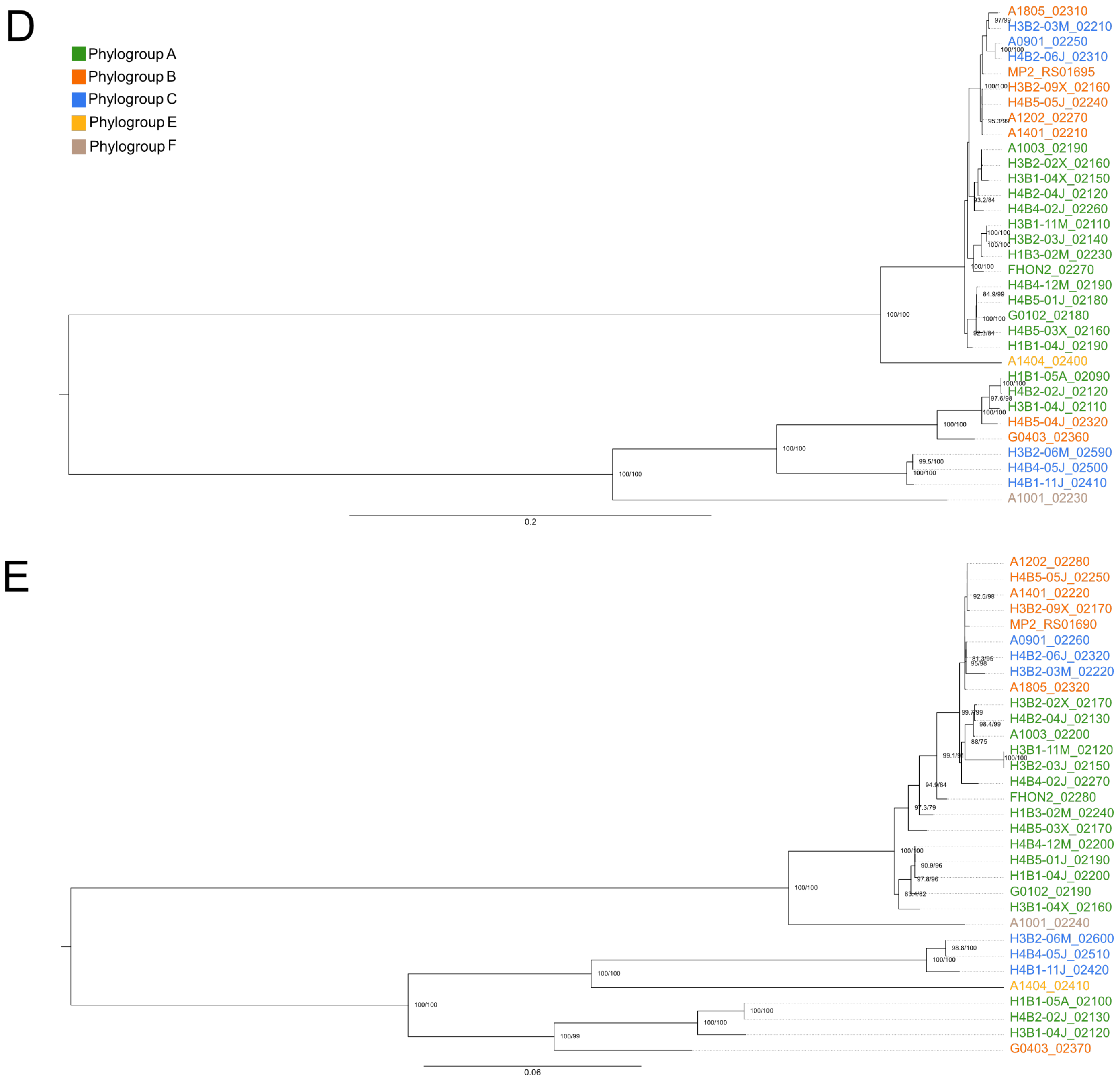
D
Phylogroup A
Phylogroup B
Phylogroup C
Phylogroup E
Phylogroup F
A1805_02310
H3B2-03M_02210
A0901_02250
H4B2-06J_02310
MP2_RS01695
H3B2-09X_02160
H4B5-05J_02240
A1202_02270
A1401_02210
A1003_02190
H3B2-02X_02160
H3B1-04X_02150
H4B2-04J_02120
H4B4-02J_02260
H3B1-11M_02110
H3B2-03J_02140
H1B3-02M_02230
FHON2_02270
H4B4-12M_02190
H4B5-01J_02180
G0102_02180
H4B5-03X_02160
H1B1-04J_02190
A1404_02400
H1B1-05A_02090
H4B2-02J_02120
H3B1-04J_02110
H4B5-04J_02320
G0403_02360
H3B2-06M_02590
H4B4-05J_02500
H4B1-11J_02410
A1001_02230
0.2
E
A1202_02280
H4B5-05J_02250
A1401_02220
H3B2-09X_02170
MP2_RS01690
A0901_02260
H4B2-06J_02320
H3B2-03M_02220
A1805_02320
H3B2-02X_02170
H4B2-04J_02130
A1003_02200
H3B1-11M_02120
H3B2-03J_02150
H4B4-02J_02270
FHON2_02280
H1B3-02M_02240
H4B5-03X_02170
H4B4-12M_02200
H4B5-01J_02190
H1B1-04J_02200
G0102_02190
H3B1-04X_02160
A1001_02240
H3B2-06M_02600
H4B4-05J_02510
H4B1-11J_02420
A1404_02410
H1B1-05A_02100
H4B2-02J_02130
H3B1-04J_02120
G0403_02370
0.06

Figure S18

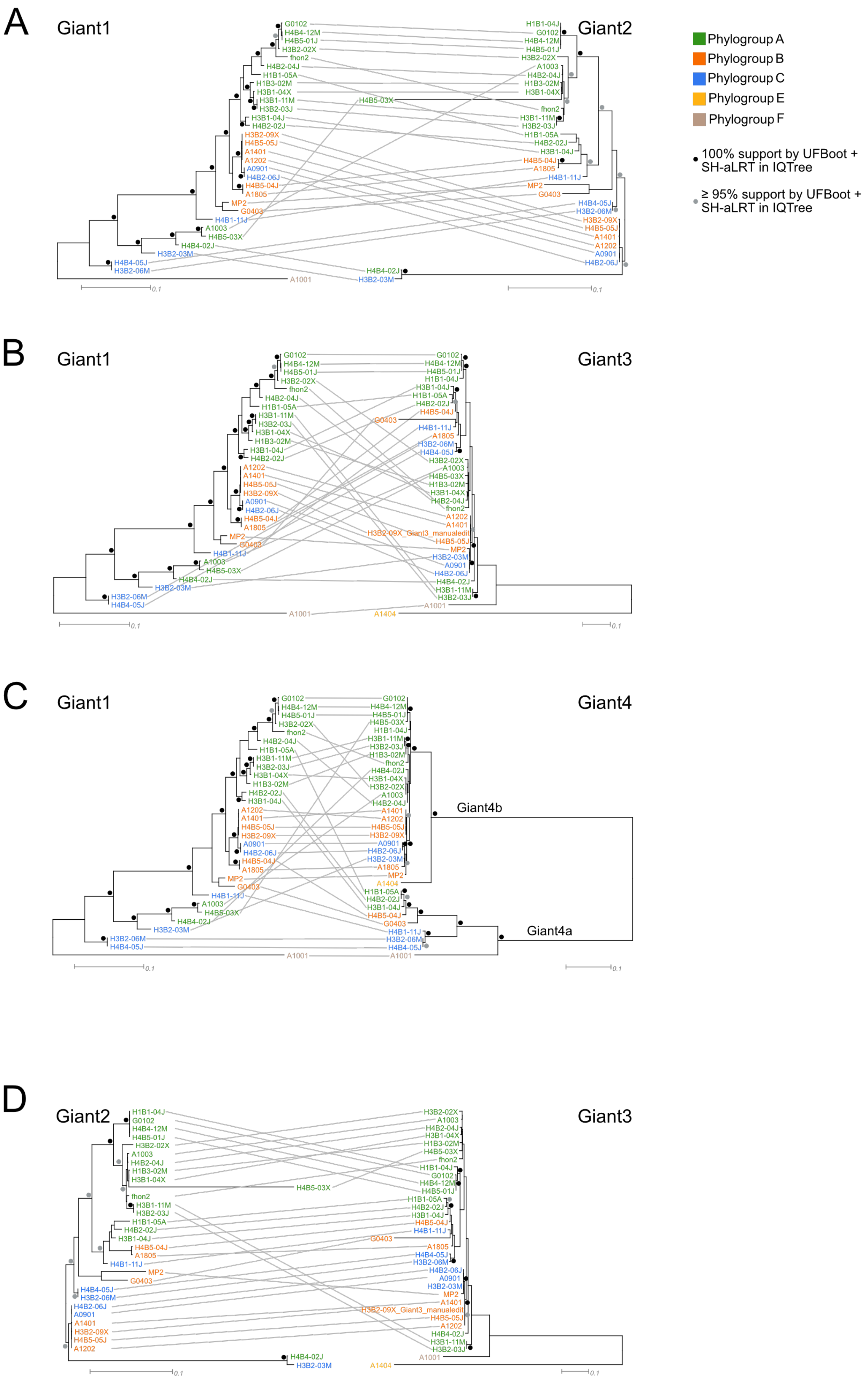
A
Giant1
Giant2
B
Giant1
Giant3
C
Giant1
Giant4
Giant4b
Giant4a
D
Giant2
Giant3
Phylogroup A
Phylogroup B
Phylogroup C
Phylogroup E
Phylogroup F
100% support by UFBoot + SH-aLRT in IQTree
≥ 95% support by UFBoot + SH-aLRT in IQTree
0.1

Figure S18

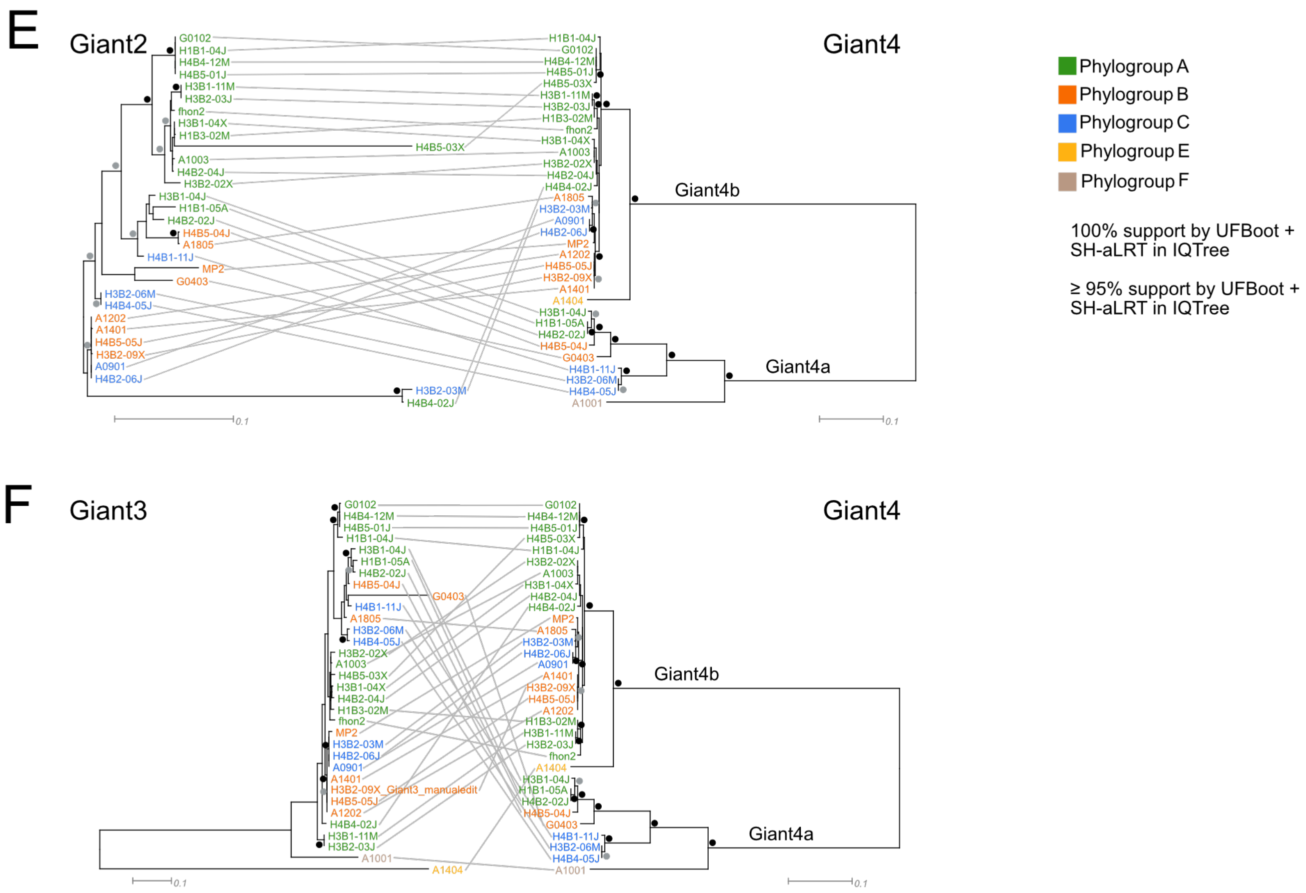
E
Giant2
Giant4
Giant4b
Giant4a
F
Giant3
Giant4
Giant4b
Giant4a
Phylogroup A
Phylogroup B
Phylogroup C
Phylogroup E
Phylogroup F
100% support by UFBoot + SH-aLRT in IQTree
≥ 95% support by UFBoot + SH-aLRT in IQTree

Figure S19

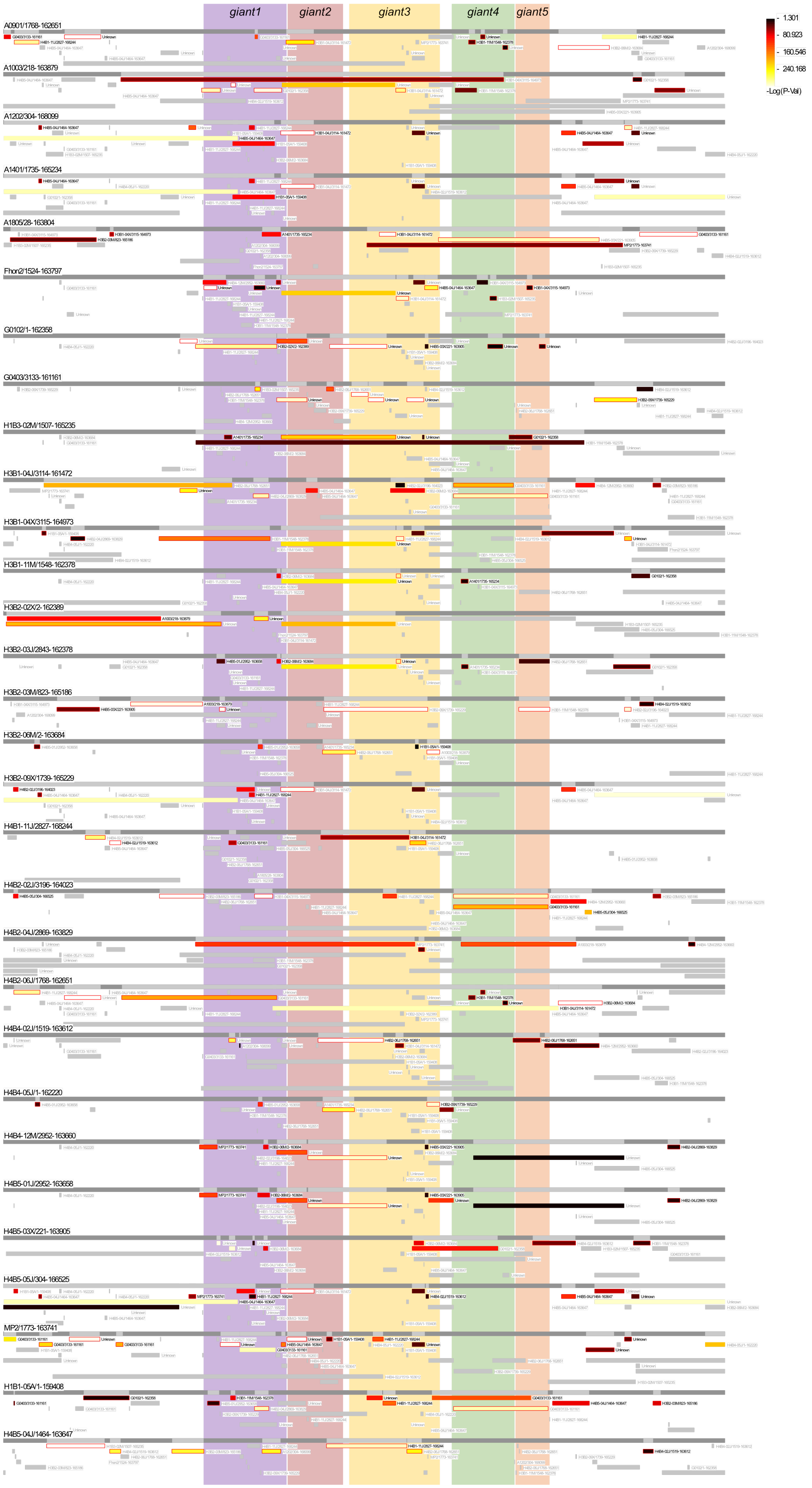
giant1
giant2
giant3
giant4
giant5
1.301
80.923
160.546
240.168
-Log(P-Val)

Figure S20

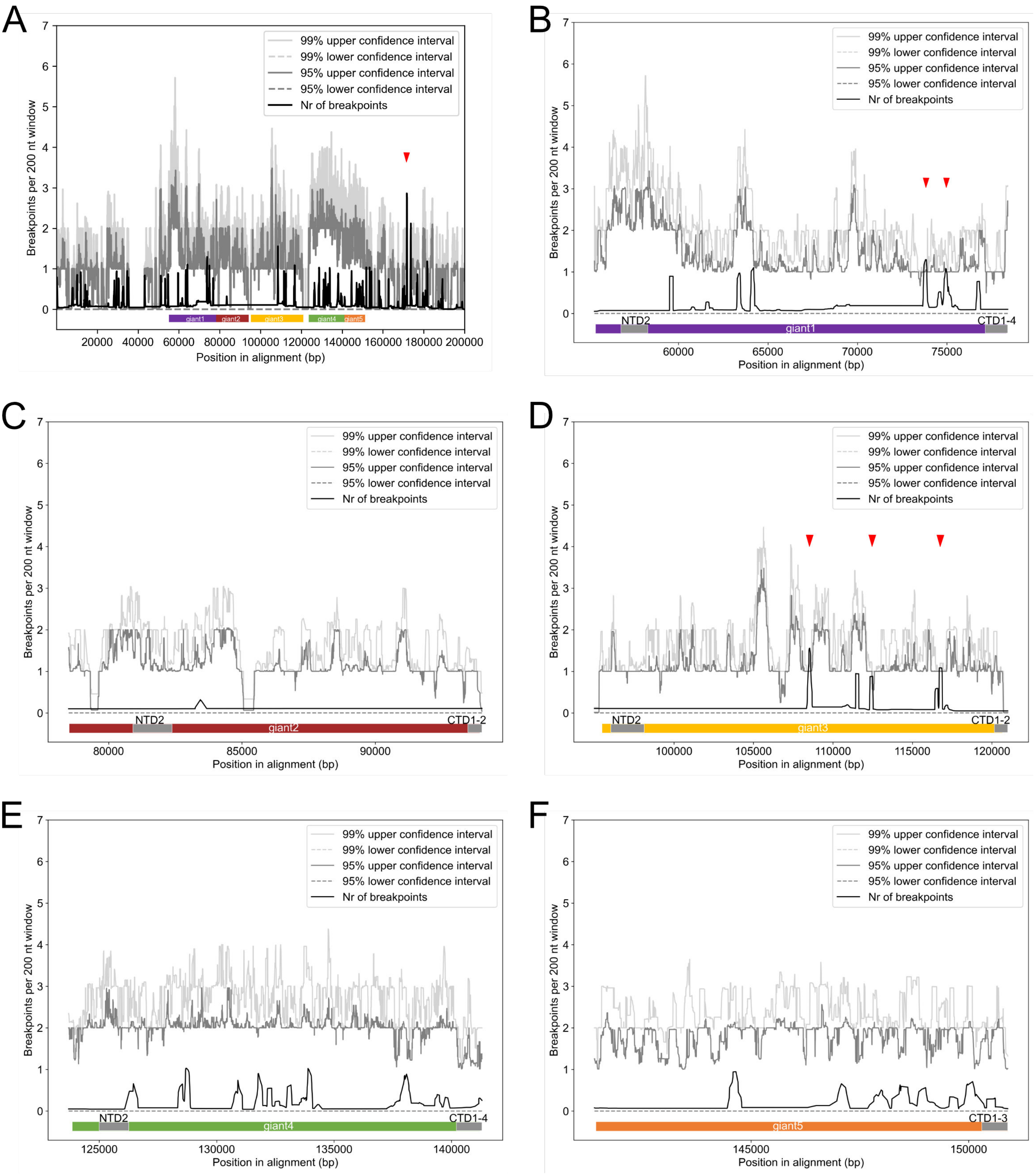
A
B
C
D
E
F
99% upper confidence interval
99% lower confidence interval
95% upper confidence interval
95% lower confidence interval
Nr of breakpoints
Breakpoints per 200 nt window
Position in alignment (bp)
giant1
giant2
giant3
giant4
giant5
NTD2
CTD1-4
CTD1-2
CTD1-3
20000 40000 60000 80000 100000 120000 140000 160000 180000 200000
60000 65000 70000 75000
80000 85000 90000
100000 105000 110000 115000 120000
125000 130000 135000 140000
145000 150000

Figure S21

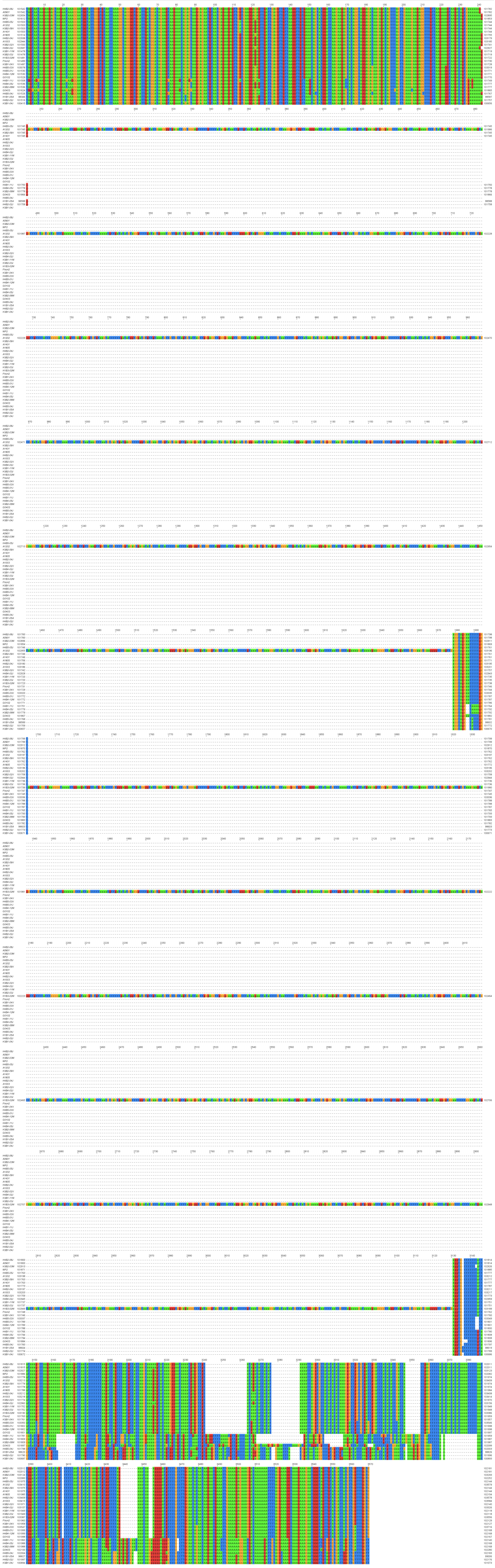

Supplement: evag011_Supplementary_Data [file evag011_supplementary_data.zip › SI.Figures_S1-S21.pdf]
